# The Effect of Behavioral Sleep Interventions on Measures of Obesity, Dietary Intake and Physical Activity in Adults With Poor Sleep Health: A Systematic Review, Meta‐Analysis and Meta‐Regression Analysis

**DOI:** 10.1111/obr.70139

**Published:** 2026-04-27

**Authors:** Samiul A. Mostafa, Pushpa Singh, Feaz Babwah, Wasim Hanif, George Balanos, Krishnarajah Nirantharakumar, Jason G. Ellis, Abd A. Tahrani

**Affiliations:** ^1^ Department of Metabolism and Systems Science University of Birmingham Birmingham UK; ^2^ Department of Diabetes University Hospitals Birmingham NHS Foundation Trust Birmingham UK; ^3^ Centre of Endocrinology, Diabetes and Metabolism (CEDAM), Birmingham Health Partner Birmingham UK; ^4^ Institute of Applied Health Research University of Birmingham Birmingham UK; ^5^ Sportex University of Birmingham Birmingham UK; ^6^ Midlands Health Data Research UK Birmingham UK; ^7^ Department of Psychology University of Northumbria Northumbria UK

**Keywords:** activity, diet, intervention, meta‐analysis, obesity, sleep disorder

## Abstract

**Background:**

As poor sleep negatively impacts on adiposity, there is interest in examining whether improving sleep can improve adiposity markers. We reviewed studies aimed at improving sleep using (1) cognitive behavioral therapy for insomnia (CBT‐I) and/or sleep hygiene and (2) sleep extension on adiposity markers, dietary intake, and physical activity.

**Methods:**

Literature searches were performed on MEDLINE, Embase, CINAHL, and Cochrane Library. We included studies featuring adults ≥ 18 years without OSA, a sleep intervention as well as preintervention and postintervention measures.

**Results:**

From a total of 27 studies, 25 were used in the meta‐analyses. Using CBT‐I and/or sleep hygiene in eight studies (*n* = 1384) led to significant BMI reductions averaging 0.64 kg/m^2^ (0.28, 1.01, *p* = 0.0006, *I* ^2^ = 16%) but the reductions were not significant in five studies (*n* = 138) of sleep extension, 0.15 kg/m^2^ (−0.40, 0.11, *p* = 0.26). In obesity (*n* = 78, 3 studies) and in type 2 diabetes (*n* = 1121, 3 studies) BMI reductions averaged 1.53 (0.49, 2.56, *p* = 0.004, *I* ^2^ = 0%) and 0.64 kg/m^2^ (0.37, 0.92, *p* < 0.00001, *I* ^2^ = 0%), respectively. For body weight, using sleep interventions led to significant reductions in the obese (*n* = 72, 3 studies) and overweight ranges (*n* = 337, 6 studies), averaging 5.55 (4.13, 6.97, *p* < 0.00001, *I* ^2^ = 0%) and 0.83 kg (0.32, 1.35, *p* = 0.002, *I* ^2^ = 0%), respectively. Using sleep interventions reduced daily energy intake (*n* = 223, 5 studies) by 147.5 cal/day (5.74, 289.41, *p* = 0.04, *I* ^2^ = 53%), but was higher in the obese/overweight range (*n* = 122, 3 studies) at 238.0 cal/day (138.86, 337.22, *p* < 0.00001, *I* ^2^ = 0%); sugar consumption reduced (*p* = 0.05), and protein consumption increased (*p* = 0.001). There were no changes in physical activity or sedentary behavior levels. On quality assessment, 12 and 10 studies were of low and some concerns, respectively.

**Conclusion:**

Addressing poor sleep health through using sleep interventions has the potential to be integrated into obesity management programs, alongside current lifestyle strategies.

**PROSPERO Identification Number:**

CRD42025644060

## Graphical abstract

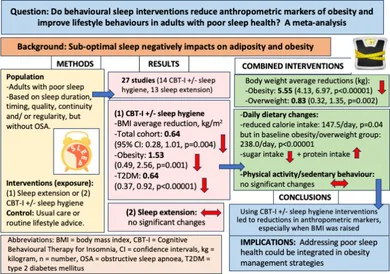

**Abbreviations**
95% CI: 95% confidence intervals
*τ* ^2^
: tau‐squared value
BMI: body mass index
CBT‐I: Cognitive Behavioral Therapy for Insomnia
cm: centimeter
CPAP: continuous positive airway pressure
CVD: cardiovascular disease
GLP‐1RA: Glucagon‐like peptide‐1 receptors agonist
GRADE: Grading of recommendations, assessments, development and evaluation
HbA1c: glycated hemoglobin
*I* ^2^
: heterogeneity test
IQR: interquartile range
ISI: Insomnia Severity Index
IV: inverse variance
kg: kilogram
mmHg: millimeters of mercury
MVPA: moderate to vigorous physical activity
*n*
: number
OSA: obstructive sleep apnea
pp: percentage point
PSQI: Pittsburgh Sleep Quality Index
RCT: randomized clinical trial
ROB‐2: Cochrane Risk‐of‐Bias Tool for Randomized Trials Version 2
SD: standard deviation
SLD: sleep duration
SSD: short sleep duration
TIB: time in bed
TST: Total sleep time
T2DM: type 2 diabetes mellitus

## 1 Introduction

Obesity is a public health concern, as an estimated 2.1 billion adults globally are classified as living with obesity or are overweight, with future projections suggesting this figure will increase [1]. As obesity is associated with multiple health complications and mortality, there is a need for novel strategies to prevent and reduce obesity that complement existing approaches [2]. This gap in the current obesity management strategies could be fulfilled through exploring the role of addressing poor sleep health.

In line with increasing rates of obesity, the emergence of another epidemic, poor or suboptimal sleep health, affects up to 30%–40% of adults [3, 4]. Such individuals exhibit subclinical dimensions of sleep health related to sleep timing, quality, duration, continuity and/or regularity, which can occur within or without a diagnosed sleep disorder [5]. The dimensions tend to overlap as they can trigger or exacerbate one another (e.g., comorbid short sleep duration and insomnia). Poor sleep is implicated in multiple cardio‐metabolic diseases including obesity, type 2 diabetes mellitus (T2DM), hypertension and cardiovascular disease (CVD) [6, 7, 8, 9]. Therefore, there is interest in examining the potential benefits of targeting sleep improvement on obesity and adiposity.

Behavioral sleep interventions have emerged as a potential solution to target sleep improvement. They are usually centered on interventions of (1) sleep extension, the intentional practice of increasing total sleep duration, often through setting earlier bedtimes or later wake times, to increase temporal factors such as time‐in‐bed (TIB) for a short sleep duration; (2) cognitive behavior therapy for insomnia (CBT‐I), a multicomponent treatment targeting difficulties with initiating and/or maintaining sleep by restructuring thoughts and behaviors related to insomnia and (3) sleep hygiene/education/counseling, a set of behavioral and environmental recommendations intended to promote healthy sleep. There is often some cross‐over in the contents of these interventions so they can address more than one subclinical sleep dimension. For example, modern CBT‐I interventions contain a module on sleep hygiene advice. In addition, as insomnia and short sleep duration can co‐exist, sleep extension can be added to CBT‐I [10].

Behavioral sleep interventions are known to improve sleep health but additionally demonstrate clinically meaningful/significant benefits on CVD risk factors, including glycaemia and blood pressure [11, 12]. That said, less is known about their impact on markers of adiposity, such as body mass index (BMI) and body weight. Two meta‐analyses briefly attempted to investigate this, however, one meta‐analysis examined sleep intervention as part of multifactorial lifestyle interventions, while the second combined various types of adiposity measures into the same outcome analysis, possibly due to the low number of studies available at the time [13, 14]. To date, no review has specifically evaluated whether improving sleep alone, through behavioral sleep interventions, affects individual adiposity outcomes (e.g., BMI). As an increasing number of published studies are available in this field, we wished to investigate this further.

Furthermore, we wished to examine if sleep interventions impact lifestyle factors related to weight including dietary and energy intake, physical activity, and sedentary behavior. Finally, we investigated how sleep interventions impact hormones regulating appetite or affecting weight and markers of inflammation, which may help to explain any findings from this analysis. These have not been investigated to date in meta‐analyses.

Sleep restriction to less than 5.5 h per day increases daily energy intake by 204 to 385 cal and alters dietary composition by increasing daily fat intake, but opposing results were obtained for the impact on daily protein and carbohydrate intake [15, 16]. Simultaneously, poor sleep health is thought to have a bi‐directional relationship with physical activity and sedentary time, but less is known on whether behavioral sleep interventions improve activity levels [17, 18].

The aim of this systematic review and meta‐analysis was to assess the impact of behavioral sleep interventions on anthropometric markers of adiposity and lifestyle measures of dietary intake and composition, physical activity, and sedentary behavior. Secondly, we wished to investigate whether the outcome results varied between different types of sleep interventions and also, according to baseline adiposity category, where sufficient data existed. We hypothesized that greater reductions in outcome measures would be observed with higher levels of adiposity at baseline (i.e., in the obesity range), which would follow the same differential impact of results observed for glycaemia and blood pressure [11, 12].

## 2 Materials and Methods

This systematic review was registered on the International Prospective Registry of Systematic Reviews (PROSPERO Identification Number: CRD42025644060) database and meets the requirements of the Preferred Reporting Items for Systematic Reviews and Meta‐Analyses statement [19].

### 2.1 Eligibility Criteria

We included intervention studies of adults ≥ 18 years old at baseline that were (a) randomized controlled trials (RCTs) or nonrandomized trials, quasi experimental, laboratory or single‐arm cross‐over (“pre‐post”) intervention studies, (b) consisted of a behavioral sleep intervention as the exposure (sleep extension, CBT‐I, and/or sleep hygiene/education/counseling), and (c) measured the outcome of interest at both baseline and follow‐up, Appendix B.

The exclusion criteria consisted of studies that (1) contained people with obstructive sleep apnea (OSA), (2) did not feature a sleep intervention, (3) consisted of interventions of CPAP/mandibular devices, bright/dim light exposure, pharmaceutical therapies, or laboratory‐based sleep restriction/deprivation as the exposure, (4) did not report measures of the same outcome marker at both baseline and follow‐up, and (5) interventions of weight loss pharmacotherapies, bariatric surgery or chrononutrition.

### 2.2 Information and Data Sources

We used the following bibliographic databases: MEDLINE, Embase, CINAHL, and Cochrane Library.

### 2.3 Search Strategy

The search strategy was developed by S.M. in collaboration with an experienced local hospital health sciences librarian. Databases were searched using a combination of appropriate subject headings for indexed articles, tailored to individual bibliographic databases, where required, and free‐text terms relevant to the framed question of this review, which were combined using Boolean operators. The full electronic search strategies used for each database are included in Appendix A. The literature search was performed from inception up to December 16, 2024; a second search was performed up to April 14, 2025.

After conducting the electronic database searches, using RefWorks reference management software duplicates were removed and two investigators (S.M. and P.S.) independently screened and assessed titles and abstracts of identified studies for eligibility. The full text of these papers was retrieved and independently assessed for inclusion by the same two reviewers. Discrepancies were resolved through additional investigators (F.B. and W.H.) where necessary until a consensus was reached. The reference lists of review articles and of included original publications were also screened for potentially relevant studies. For any relevant trial protocols derived from the database searches, the corresponding publication was searched for separately. We included studies of any language published as full‐text papers.

### 2.4 Study Selection and Outcomes

The study inclusion and exclusion criteria are listed above. The sleep variables considered included the Insomnia Score Index (ISI) score, Pittsburgh Sleep Quality Index (PSQI) global score, TIB, and sleep duration [20]. The outcomes for anthropometric markers of adiposity included BMI, body weight, percentage body weight change, waist circumference, hip circumference, combination ratio measurements (e.g., waist:hip, waist:height ratio), percentage body fat or fat‐free mass. These outcomes could be assessed through human measurement or using equipment (e.g., bioimpedance devices or dual‐energy x‐ray absorptiometry scans). If the outcome included studies of self‐reported measures, we conducted a sensitivity analysis of the principal outcomes without these studies. The dietary outcomes considered were daily measures for calorie intake and total energy expenditure, which could be assessed using validated diaries or questionnaires, indirect calorimetry or doubly labeled water methods, but not self‐monitoring food applications, which may not validated for research purposes. Additionally, we analyzed for composition of diet (e.g., sugar and salt) as quantity per day or percentage of total daily intake. The outcomes for activity were light activity, moderate or vigorous physical activity (MVPA), total activity as metabolic equivalents per week, daily step count, sitting time, and sedentary behavior. These measures could be assessed using validated questionnaires, pedometers, smart devices, or tri‐axial accelerometers. Finally, we performed a secondary analysis for the effect of sleep interventions on hormones and inflammatory markers that may regulate or affect appetite and weight.

If data in a study were either missing or unclear (e.g., BMI was presented but not body weight, or if data were presented on a graph but not numerically), we contacted the lead author for further information. Otherwise, the study could not be included for the specific outcome.

In the case of a two‐arm study, the comparator was considered as either a control group (i.e., no intervention), a lifestyle program/advice session consisting of diet and exercise modifications (considered as standard care) or certain other lifestyle interventions (e.g., Tai Chi). If the sleep intervention was part of a multifactorial lifestyle intervention, the study was included only if control arm consisted of the same lifestyle intervention without the sleep component. For this reason, in the case of any three‐arm studies, we only included data from two arms.

### 2.5 Data Extraction

One investigator (S.M.) extracted the data and the second investigator (PS) independently checked for consistency. Data were extracted on the following: first author's surname, year of publication, country of origin of the population studied, type of study (e.g., randomized trial), sample size of intervention and control arms, population demographic characteristics (e.g., age, sex), sleep variable(s) under analysis (subjective and/or objective measures), type of sleep intervention, length of follow‐up, measure(s) used to assess change in sleep variable, type of outcome marker(s) measured, and levels of the sleep and outcome marker(s) at baseline and follow‐up.

Sleep interventions were placed into two categories. Firstly, we used (a) CBT‐I and/or sleep hygiene, which have a common focus on improving outcomes of sleep quality or insomnia symptoms (e.g., PSQI global and ISI scores, 20). In addition, sleep hygiene is routinely incorporated into modern CBT‐I interventions. Secondly, we used (b) sleep extension, which focuses on increasing sleep duration (SLD), total sleep time (TST), and time in bed (TIB). These two categories follow those used in previous systematic reviews [11, 12, 21].

### 2.6 Study Risk of Bias Assessment

Study quality assessment was performed independently by two reviewers (either F.B., P.S., or S.M.) who met to compare results and reach consensus. Discrepancies were resolved through a fourth investigator (W.H.) where necessary until a consensus was reached. The Cochrane risk‐of‐bias tool for randomized trials version 2 (ROB‐2) tool was used for RCTs, which includes five assessments of possible bias including randomization process, deviation from proposed interventions, missing outcome data, outcome measurement, and result reporting [22]. Additionally, the Risk‐of‐Bias in Nonrandomized Studies of Interventions' (ROBINS‐I) tool was used for nonrandomized or single‐arm cross over (“pre‐post”) studies [23]. ROBINS‐I has seven assessments of potential bias of confounding, selection, intervention classification, deviation of interventions, missing data, outcome measurement, and result reporting. Studies were judged to have low, moderate, or high risk of bias. The results of the risk of bias assessments were converted into traffic‐light and weighted summary plots using Robvis software [24].

#### 2.6.1 Statistical Analysis

Where sufficient information existed, we analyzed data quantitatively using meta‐analysis. For all the main outcomes, the threshold for inclusion was three studies, but simultaneously we accepted that for some less commonly measured secondary outcomes (e.g., total energy expenditure) only two studies may be available. Initially we analyzed all studies together (i.e., RCTs and non‐RCTs combined) before analyzing these separately. The non‐RCTs were nonrandomized trials or “pre‐post” single‐arm cross‐over studies where the same population of participants underwent the first phase with no intervention (considered control), then undertook the sleep intervention (considered intervention), followed by final measurements.

The pooled results from all sleep interventions were analyzed firstly together and then separately as studies of [1] sleep extension or [2] CBT‐I and/or sleep hygiene. Our preference was to use intention‐to‐treat analyses to minimize the impact of bias. Hence, in cases where studies reported data as both intention‐to‐treat and per protocol analyses, we selected intention‐to‐treat analyses. Secondly, we performed three subgroup analyses of the main pooled results. Firstly, according to the baseline (preintervention) BMI categories of individual studies, classified using ethnic specific cut‐points according to the baseline mean cohort BMI level [2]. The second subgroup analysis was conducted according to the baseline sleep‐problem phenotype within individual studies of (a) Insomnia, based on ISI score > 10 or fulfilling criteria according to International Classification of Sleep Disorders [25]; (b) circadian misalignment for an evening chronotype detected on a recognized questionnaire; (c) short sleep if objectively measured sleep duration < 6.5 h (or time‐in‐bed/total sleep time < 7 h); or (d) clinically significant sleep disorder but without qualification of exact sleep problem (e.g., PSQI global score > 5). The final subgroup analysis was performed in people with T2DM if three or more studies were available.

The outcome variables were reported as mean (standard deviation [SD]). When the original study reported outcomes as median (interquartile range [IQR]) or mean (95% confidence intervals [95% CI] or standard error) these were converted to mean (SD) using accepted methods [26]. We estimated the pooled mean differences (95% CI) of the variables of interest, considered as the effect size, for outcomes of adiposity, dietary intake, and activity measures. Pooled differences were calculated using the inverse variance method and through using a random‐effects model, as we anticipated a degree of study‐level variability in the exact interventions used. For the hormone and biomarker analysis, the units of each hormone were uniformed across studies. Additionally, because of the possible variations in the hormone assays used, we calculated standardized mean differences instead. Heterogeneity among studies was quantified by *I* ^2^ statistic. We considered low, moderate, and high *I* ^2^ values to be 25%, 50%, and 75%, respectively [27]. For outcomes that had significant differences between intervention and control, we also reported the Tau‐squared (*τ* ^2^) value.

#### 2.6.2 Sensitivity Analysis

We performed sensitivity analyses to check for robustness of data for the main outcomes of the meta‐analysis, where a sufficient number of studies was available. Firstly, the risk of publication bias was assessed through visual inspection of funnel plots for asymmetry and by calculating the Egger's regression–based test before and after removal of smaller studies. A small study was defined as having less than 60 participants [28]. Secondly, a “leave‐one‐out” analysis was conducted for the meta‐analysis results by removing one study at a time from the dataset and before rerunning the meta‐analysis to see how the overall results changed. Thirdly, to assess for the overall impact of risk of bias on the results, studies within the meta‐analyses were categorized according to allocated ranking (low risk, some concern, or high risk) before rerunning the meta‐analysis results for these categories if they contained three or more studies. Finally, for dietary and daily energy outcomes, we conducted a sensitivity analysis according to the nutritional assessment method used. Once these analyses were completed, a Grading of Recommendations, Assessments, Development, and Evaluation (GRADE) table was made [29].

#### 2.6.3 Meta‐Regression Analysis

Where the pooled prevalence of outcomes had more than 10 studies with a moderate or high heterogeneity on meta‐analysis, meta‐regression models were produced to identify any confounding variables, which could have influenced the effect of the interventions on the outcome. A total of 13 univariate meta‐regression analyses were conducted using random effects model. Covariates considered were age, sex, geographical location (Europe vs. other), study design (RCT vs. non‐RCT), intervention type, intervention delivery (face‐to‐face vs. remote/digital), length of study follow‐up, year of publication, baseline BMI level, baseline BMI category, baseline PSQI global score, and history of T2DM. Statistical analyses were performed with Review Manager (RevMan) software Version 5.4.1 (Copenhagen, Cochrane Collaboration) for the meta‐analyses and with IBM SPSS Statistics for Macintosh, Version 30.0 (IBM Corp, Armonk, NY, USA) for meta‐regression analysis and risk of publication bias assessments. All tests were two‐sided with a *p* level of *p* < 0.05 considered as statistically significant.

## 3 Results

### 3.1 Identification of studies

There were 511 abstracts derived from the search strategy, of which 55 met inclusion criteria for full‐text analysis; of these, 27 studies were included in the systematic review (Figure 1; [30, 31, 32, 33, 34, 35, 36, 37, 38, 39, 40, 41, 42, 43, 44, 45, 46, 47, 48, 49, 50, 51, 52, 53, 54, 55, 56]). The reasons for excluding the 28 studies consisted of insufficient data/no full‐text paper available (*n* = 16), incorrect intervention (*n* = 2), incorrect population (*n* = 2), incorrect outcome measure (*n* = 2), incorrect comparator (*n* = 5), and when further clarifications about data were not provided (*n* = 1).

**FIGURE 1:**
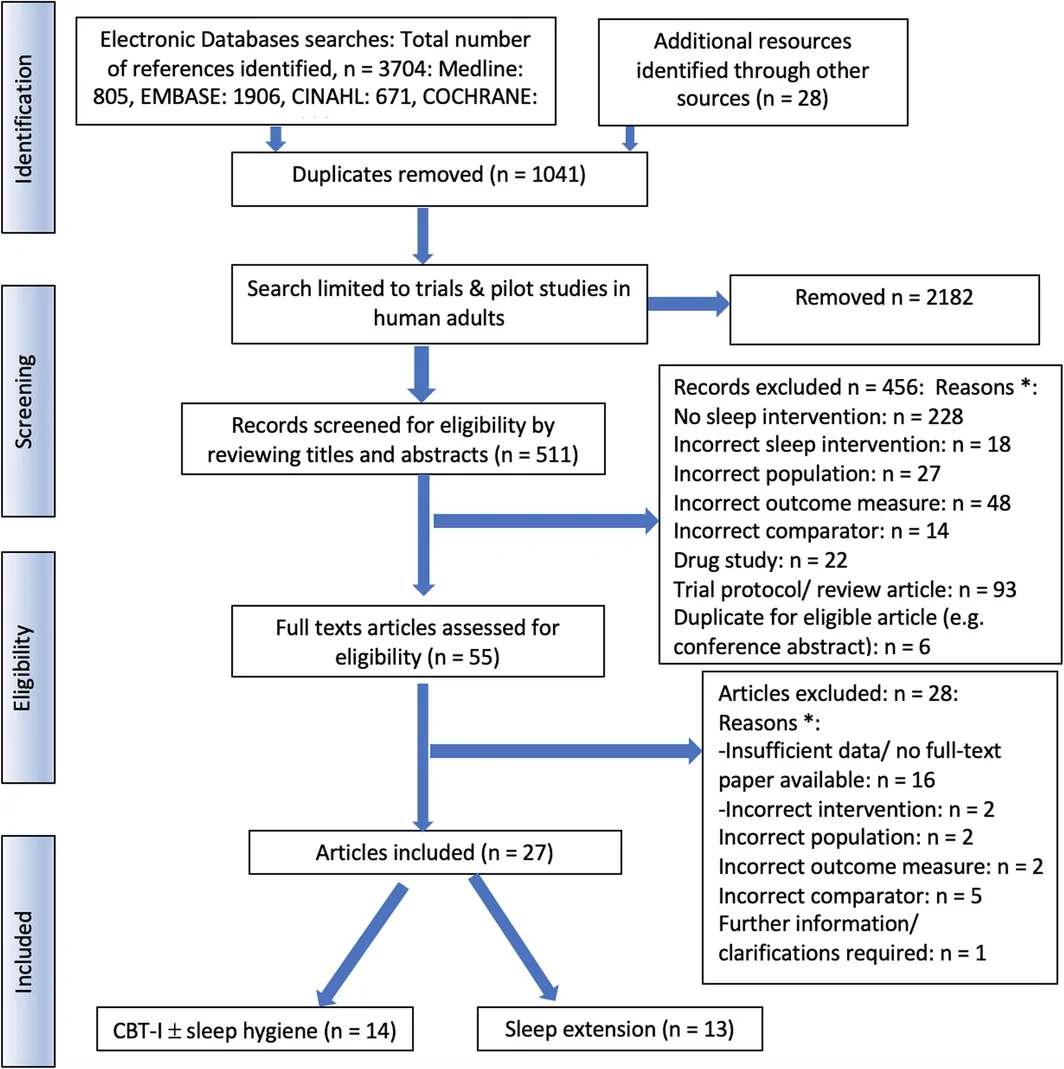
Flow chart to demonstrate the process of study selection. CBT‐I = cognitive behavior therapy for insomnia, *n* = number. *For reasons excluded, there may have been more than one reason, however the allocated category represents the first obvious reason for exclusion.

### 3.2 Description of the Included Studies and Interventions

From 27 studies, which were published between 2011 and 2024, 21 were RCTs, five were “pre‐post” studies and the remaining study was a nonrandomized feasibility study. Fourteen studies used a CBT‐I and/or sleep hygiene intervention and 13 used a sleep extension intervention. Table 1 describes the baseline characteristics of the studies. The studies originated from the Americas (11 studies from USA), Europe (2 from the UK and one each from the Germany, Holland, Turkey and Belgium), Asia (2 from China and one each from Korea, Taiwan, Japan and Thailand), and Oceania (3 from Australia and 1 from New Zealand). The length of follow‐up ranged from 3 to 12 months for CBT‐I and/or sleep hygiene and from 2 weeks to 2.7 months for sleep extension.

**TABLE 1:** Baseline characteristics of the included studies.

					Intervention				Control			
First author, year, country	Population	Study type	Intervention (vs. control)	Study length	*n*	Age, years	Male %	Baseline measurements of outcomes + sleep measurements^a^	*n*	Age, years	Male %	Baseline measurements of outcomes + sleep measurements^a^
**(a) Cognitive behavior therapy for insomnia (CBT‐I) and/or sleep hygiene studies**												
Chen, 2011, Taiwan	Dialysis patients with sleep disturbance	RCT (used in hormonal analysis only)	CBT‐I three times per week vs. control (basic sleep advice)	6 weeks	37	58 (10)	45.9	PSQI = 13.4 (3.5), SLD = 5.0	35	59 (11)	37.1	PSQI = 12.2 (3.0) SLD = 5.7
Chesla, 2020, USA	Females with MetS or PDM and sleep duration < 6.5 h (TIB < 7)	Non‐randomized 2‐arm trial	Lifestyle + sleep vs. Lifestyle (8 sessions over 3 months + telephone support)	6 months	12	52.9 (11.3)	0	TST = 6.3 (0.9), BMI = 31.1 (3.7)	14	53.8 (12.7)	0	TST = 6.5 (0.7) BMI = 32.0 (3.8)
Duncan (Move, Eat, Sleep), 2020, Australia	Obese + overweight adults	3‐arm RCT, only 2 arms compared: enhanced vs. traditional	Sleep + lifestyle vs. lifestyle	12 months	14	47.2 (9.4)	17.3	ISI = 9.9 (4.6) PSQI = 7.3 (2.8) SLD = 384 (54) BMI = 31.9 (4.0) WC = 99.5 (12.5) Sitting time = 667.4 (194.5) Sedentary time = 606 (143.5) MVPA = 56.6 (34.0) LPA = 199.8 (74.3)	23	45.4 (10.2)	26.8	ISI = 8.5 (5.2) PSQI = 7.0 (3.1) SLD 384 (54) BMI = 31.4 (3.8). WC = 99.6 (9.0) Sitting time = 736.8 (212.2) Sedentary time = 629.7 (98.4) MVPA= 56.3 (33.0) LPA = 196.1 (79.1)
Duncan (REFRESH), 2020, Australia, study	Overweight adults with poor sleep quality	3‐arm RCT, Only 2 arms compared: Sleep only vs. control	Sleep intervention (smartphone “app”) vs. control	6 months	110	52.2 (7.0)	16.4	PSQI = 10.4 (3.11) BMI = 28.5 (4.4) Sitting time = 656.58 min/day (184.71) MVPA = 105 min/week (60–180)	55	52.4 (7.3)	15.5	PSQI = 10.67 (3.05) BMI = 28.3 (4.1) Sitting time = 652.86 min/day (176.47) MVPA = 180 min/week (60–320)
Erim, 2024, Turkey	Obese + evening chronotype	RCT	Sleep hygiene (1 session + website) + nutritionist plan vs. nutritionist plan only	12 weeks	19	40.8 (9.9)	15.8	PSQI = 8.2 (3.6) Weight = 88.5 (10.6) BMI = 32.5 (1.8) WC = 99.0 (9.5)	19	41.0 (10.0)	15.8	PSQI = 7.6 (4.4) Weight = 86.2 (6.8) BMI = 32.2 (1.6) WC = 100.0 (6.1)
Groeneveld, 2024, Holland	T2DM with insomnia symptoms	RCT	CBT‐I (vs. control)	6 months	29	67.7 (8.4)	75.9	ISI = 15.3 (3.7) TIB = 8.4 (1.2) SLD (diary) = 354 SLD (actigraph) = 402 Efficiency = 84.7% BMI = 30.0 (7.0)	28	71.8 (7.3)	57.1	ISI = 13.1 (4.1) TIB = 8.8 (1.4) SLD (diary) = 378 SLD (actigraph) = 444 Efficiency = 86.9% BMI = 29.9 (4.7)
Ham, 2020, Korea	Insomnia with poor sleep quality	RCT	CBT‐I sessions x4 + sleep hygiene (vs. 1 session of group education)	12 months	24	53.83 (6.64)	0	ISI = 11.29 (4.96). PSQI = 8.46 (2.72) BMI = 24.42 (2.81)	20	55.45 (4.43)	0	ISI = 12.85 (6.13) PSQI = 8.60 (3.22) BMI = 23.91 (2.69)
Irwin, 2014/15, USA	Older adults with insomnia	3‐arm RCT, only 2 arms compared: CBT‐I vs. Tai Chi (biomarker analysis only)	Weekly sessions over 4 months	16 months	50	64.4 (6.1)	22.0	PSQI = 10.4 (2.9) BMI = 25.4 (3.3) TST = 370 (52.2)	48	66.3 (7.4)	35.4	10.7 (3.1) BMI = 26.4 (4.0) TST 378.9 (41.2)
Irwin, 2024, USA	Insomnia in breast cancer survivors	CBT‐I vs. Tai Chi (biomarker analysis only)	Weekly sessions over 4 months	15 months	45	60.0 (9.3)	0	BMI = 26.2 (5.9)	45	59.6 (7.9)	0	BMI = 25.6 (4.5)
Ito‐Masui, 2023, Japan	Shift workers with PSQI global > 5	Prestudy and poststudy	CBT‐I, online for 4 weeks	4 weeks	61	32.9 (8.3)	18	PSQI = 9.10 (4.1) SLD =332.4 (81.6) Steps/day =7678 (2922)				
Johann, 2020, Germany	Adults with insomnia disorder	RCT (used in the biomarker subanalysis only)	CBT‐I, face‐to‐face, 50 min session, once weekly × 8 weeks (vs. waiting list control)	8 weeks	23	40.8 (14.0)	39.1	ISI = 15.4 (4.3)	23	41.2 (15.1)	34.8	ISI = 14.5 (5.0)
Li, 2018, China	T2DM with HbA1c > 8% and SSD < 6 h	RCT	2 × 30‐min sleep education + T2DM education (vs. T2DM education)	3 months	16	53.7 (17.2)	63	PSQI = 7.6 (3.7) BMI = 25.8 (3.2)	15	51.6 (12.2)	60	PSQI = 7.3 (4.1) BMI = 26.1 (3.1)
Logue, 2012, USA	Obese or overweight adults with poor sleep	RCT	Weekly sleep hygiene + lifestyle sessions vs. lifestyle only	12 weeks	23	39.1%: 40–64 years old	8.7	BMI = 25–29: 21.7% BMI = 30–34: 39.1% BMI = 35–39: 39.1%	23	60.9%: 40–64 years old	17.4	BMI = 25–29: 21.7% BMI = 30–34: 30.4% BMI = 35–39: 47.8%
Zhang, 2021, China	T2DM + poor sleep quality	Cluster RCT	CBT‐I: 6 sessions + lectures over 6 weeks (Vs usual care)	12 months	568	61.6 (9.2)	34.9	PSQI = 10.07 (2.38) BMI = 24.8 (4.2)	574	61.8 (8.6)	33.3	PSQI = 10.04 (2.37) BMI = 25.2 (4.9)
**(b) Sleep extension studies**												
Al‐Khatib, 2018, UK	SSD 5 to < 7 h h/night	RCT	45‐min sleep consultation session on sleep extension/hygiene	4 weeks	21	24.0 (21.5–29.0)	14.3	PSQI = 6.8 TIB 423 SLD = 328 Efficiency = 86.2% BMI = 22.5 (21.3, 29.0) ^$^	21	25.0 (22.5–30.5)	19.0	PSQI = 5.2 TIB = 429 SLD 353 Efficiency 82.0% BMI = 21.8 (20.9, 22.8) ^$^
Cizza, 2014, USA	Obese (BMI = 30–55), SSD < 6.5 h/night	RCT but only pre randomization data considered, as a single arm	Only preintervention data analyzed—RCT ended early before any intervention started	Median 2.7 months	125	40.6 (6.9)	24	SLD (actigraph) = 344.4 Efficiency = 79.5% BMI = 38.4 (6.1)	—	—	—	—
Gonzalez, 2024, USA	Short sleep duration	Prestudy and poststudy	Acute sleep extension	2 weeks	22	60 (15)	27.3	TST= 405 (45) Efficiency = 86% BMI = 25 (6) LPA = 868 (801, 916) MVPA = 23 (13, 40) Steps/day = 6437 (2920)	—	—	—	—
Haack, 2014, USA	SSD < 7 h/night + prehypertension/stage 1 hypertension	RCT	Advised to increase TIB by 1 h/night, sleep hygiene advice (vs. sleep hygiene)	6 weeks	13	46.9 (12.3)	46.1	SLD 378 min (12) BMI = 26.5 (1.2)	9	48.4 (12.3)	22.2	SLD 372 min (18) BMI = 26.0 (1.2)
Hartescu, 2021, UK	SSD < 6.5 h/night	RCT	Advised to increase TIB by 1 h/night, counseling sessions of CBT principles	6 weeks	10	39.9 (8.8)	100	PSQI = 10.8 SLD 344.2 TIB = 398.4 Efficiency = 85.8% BMI = 29.3 (1.6)	8	43.0 (10.1)	100	PSQI = 9.44 SLD 349 min TIB 431.6 Efficiency = 80.7% BMI = 29.8 (4.2)
Leproult, 2015, Belgium	Nonobese adults with SSD < 7 h/night	Single arm prospective trial (“pre‐post” study)	Advised to increase TIB by 1‐h/night	6 weeks	16	25.3 (3.6)	18.8	PSQI = 4.8 (2.5) TIB = 390 (348–408) BMI = 21.3 (3.5) Weight = 60 (9.6)	—	—	—	—
Killick, 2015, Australia	Short sleep duration < 6.5 h during the week	Randomized cross‐over study (2 arms compared for hormone subanalysis only)	Lab study: TIB of 6 or 10 h/night	3 nights	8	28.6 (2.0)	100	BMI = 26.0 (0.8) SLD midweek = 372 SLD Weekend = 510	—	—	—	—
Mathew, 2024, USA	College students > 18 years with TST < 7 h	Prestudy and poststudy	Advised to increase TIB by 1 h/night	1 week	11	20.3 (1.5)	17	TST = 6.93 (0.83) ESS = 8.08 (4.17) BMI = 24.5 (3.5) MVPA = 67.4 Sedentary time = 676.3	—	—	—	—
Reutrakul, 2022, USA	SSD < 7 h/night and hx of gestational diabetes	RCT	Sleep extension vs. healthy living information	6 weeks	9	42.0 (2.9)	0	PSQI = 7.8 SLD 369.6 Efficiency = 88.2% BMI = 32.7 (5.3) Weight = 85.7 (14.3)	5	38.7 (6.0)	0	PSQI = 6.4 SLD 408.9 Efficiency = 90.7% BMI = 32.6 (4.6) Weight 85.7 (13.5)
Reynold, 2014, USA	Sleep duration 6–9 h, mean age 31.7, 28.6% male, BMI < 30	RCT	Increase TIB by 3 h/night (vs. control)	1 week	8	—	—	TIB (acti) = 407 (35) ESS = 4.50 (3.42) PSQI = 3.88 (0.99) Steps/days = 6442.39 (1772.4)	6	—	—	TIB (acti) = 417 (21) ESS = 4.67 (2.73) PSQI = 3.0 (1.9) Steps/days = 5662.3 (1625.1)
So‐ngern, 2019, Thailand	SSD < 6 h/night	Randomized Cross‐over study (21‐day washout)	Advised to increase TIB by 1 h/night (vs. sleep hygiene advice)	2 weeks	21	33.6 (6.1)	10	SLD = 354 (41) Efficiency = 84.8% BMI = 22.6 (3.2)	—	—	—	—
Swinbourne, 2018, New Zealand	Reduced sleep quality in athletes	Prestudy and poststudy (hormone analysis only)	Advised to increase sleep to 10 h/night	6 weeks	25	25 (2.7)	100	Weight =105 kg (12.1) TIB =529.3 (38.2) TST = 422.4 (38.2) Salivary cortisol = 6.0 nM	—	—	—	—
Tasali, 2022, USA	SSD < 6.5 h/night	RCT	Sleep counseling session to extend TIB to 8.5 h vs. control	2 weeks	40	29.3 (4.7)	50	SLD = 354 (36) BMI = 28.1 (1.3)	40	30.3 (5.5)	52.5	SLD = 360 (30) BMI = 28.1 (1.5)

*Note:* Continuous variables represented as mean (SD) or median (IQR), except for ^$^ represents the geometric mean with 95% CI.

^a^ Units for variables: BMI in kg/m^2^, MVPA and LPA in minutes/day, SLD, TIB, and TST in minutes, waist circumference in centimeters, weight in kilograms.

Abbreviations: acti = actigraph derived results, BMI = body mass index, CBT‐I = cognitive behavior therapy for insomnia, GDM = gestational diabetes mellitus, IQR = interquartile range, ISI = insomnia severity index score, kg = kilograms, LPA = light physical activity, MetS = metabolic syndrome, min = minutes, MVPA = moderate to vigorous physical activity, *n* = number, PDM = prediabetes, PSQI = Pittsburgh Sleep Quality Index global score, RCT = randomized controlled trial, SD = standard deviation, SLD = sleep duration, SSD = short sleep duration, T2DM = type 2 diabetes mellitus, TIB = time in bed, TST = total sleep time, WC = waist circumference.

### 3.3 Description of Study Participant Characteristics

Considering the baseline cohort characteristics, the mean age was 54.3 years (SD = 14.2), mean BMI = 26.6 kg/m^2^ (4.1) and 26.2% were male. Where reported, the mean levels for PSQI global score (15 studies), ISI (5 studies), sleep duration (13 studies), and time‐in‐bed (6 studies) were 9.7 (2.4), 13.2 (2.3), 357.4 (34.0), and 461.5 min (48.5), respectively. Studies, which focused on sleep extension interventions usually, did not measure PSQI global or ISI scores and vice versa for studies of CBT‐I and/or sleep hygiene interventions.

### 3.4 Description of Assessment of Study Quality

From the 26 studies considered for study quality, 12 studies had low concern throughout all categories assessed, with other studies having at least some concern, as demonstrated in the traffic‐light plots, Appendix C. The overall risk of bias was assessed through summary plots, which demonstrated some concern for RCTs and high risk of bias for nonrandomized studies, respectively.

### 3.5 Description of Outcomes

#### 3.5.1 Effect of Sleep Interventions on Anthropometric Markers of Adiposity Outcomes

From 17 studies analyzing adiposity markers, of which 16 were RCTs, there were significant reductions observed in BMI in two studies (Erim and Li), body weight in two studies (Erim and Tasali), and waist circumference in one study. Two studies that were included in the systematic review, but not the meta‐analysis, reported relevant findings. The RCT of Cizza et al. was terminated early, as within the 2.7 months between baseline and screening, 125 participants significantly increased their sleep duration, on average by 31 min, without undertaking any intervention, which led to significant reductions in levels of waist circumference, from mean 114.6 cm (SD = 13.3) to 113.8 cm (13.1), respectively, *p* = 0.038, but not BMI or body weight change [45]. Secondly, Logue et al. conducted a 12‐week RCT comparing sleep hygiene and lifestyle advice and compared to lifestyle only in 46 participants with obesity or overweight [42]. The percentage weight loss in the two groups after 12 weeks was 5% and 2%, respectively, *p* = 0.04; exact values were not available.

#### 3.5.2 Meta‐Analysis

Data from 25 of the 27 studies were extracted to conduct meta‐analyses comparing use of a sleep intervention to control in all cases (Forrest plots presented in Figures 2, 3, 4 and Appendices C–L; graphical abstract).

**FIGURE 2:**
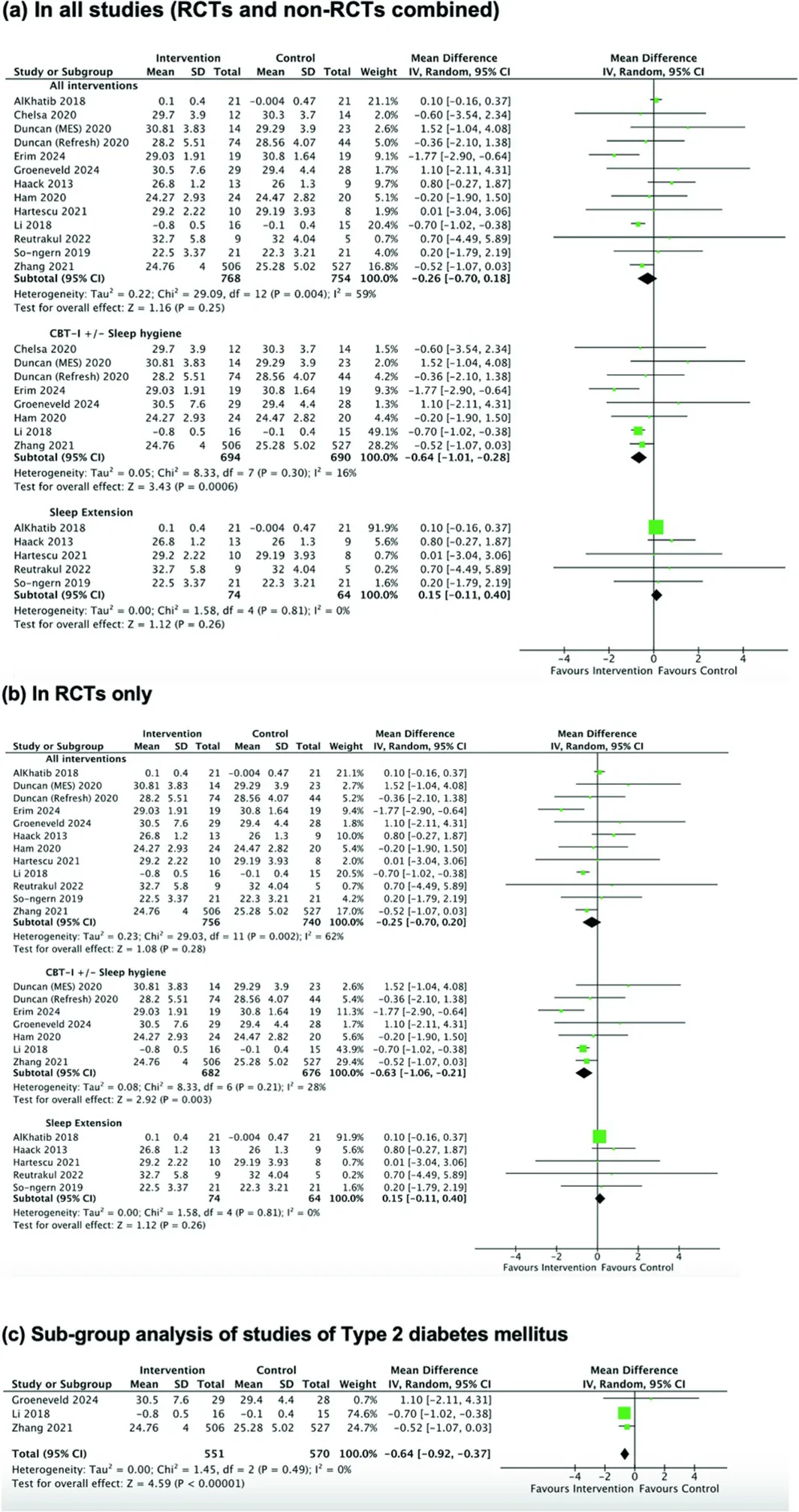
Forrest plots for the results of the meta‐analysis assessing the effect of sleep interventions on body mass index (a) in all studies, (b) in RCTs only, and (c) in type 2 diabetes mellitus. BMI measured in kg/m^2^. BMI = body mass index, CI = confidence interval, CBT‐I = cognitive behavioral therapy for insomnia, *I* ^2^ = heterogeneity test, IV = inverse variance, kg = kilogram, RCT = randomized controlled trial, SD = standard deviation.

**FIGURE 3:**
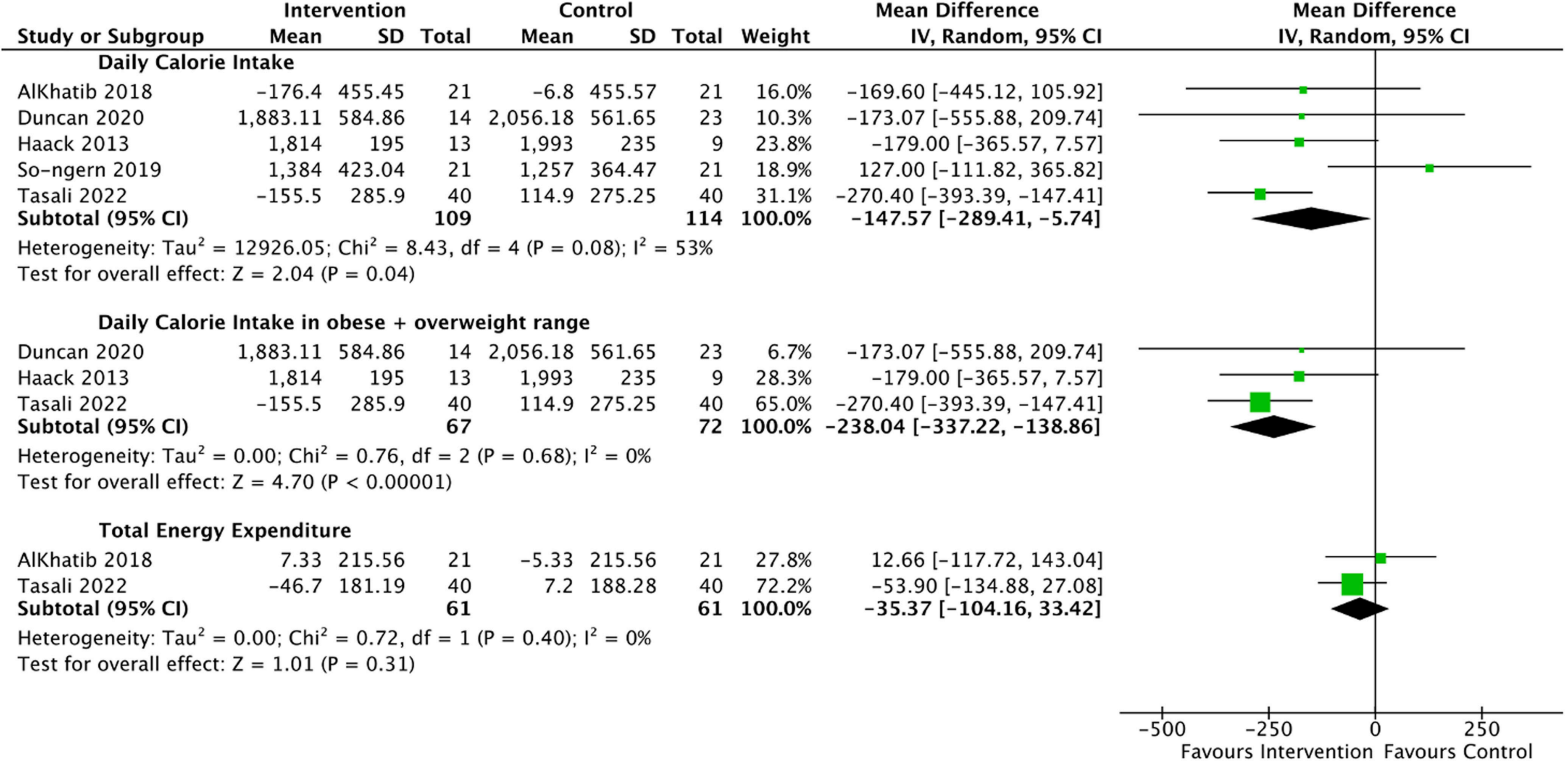
Forrest plots for the results of the meta‐analysis assessing the effect of sleep interventions on daily energy intake in (a) all studies and (b) in obese or overweight range and (c) total energy expenditure. CI = confidence interval, *I* ^2^ = heterogeneity test, IV = inverse variance, SD = standard deviation.

**FIGURE 4:**
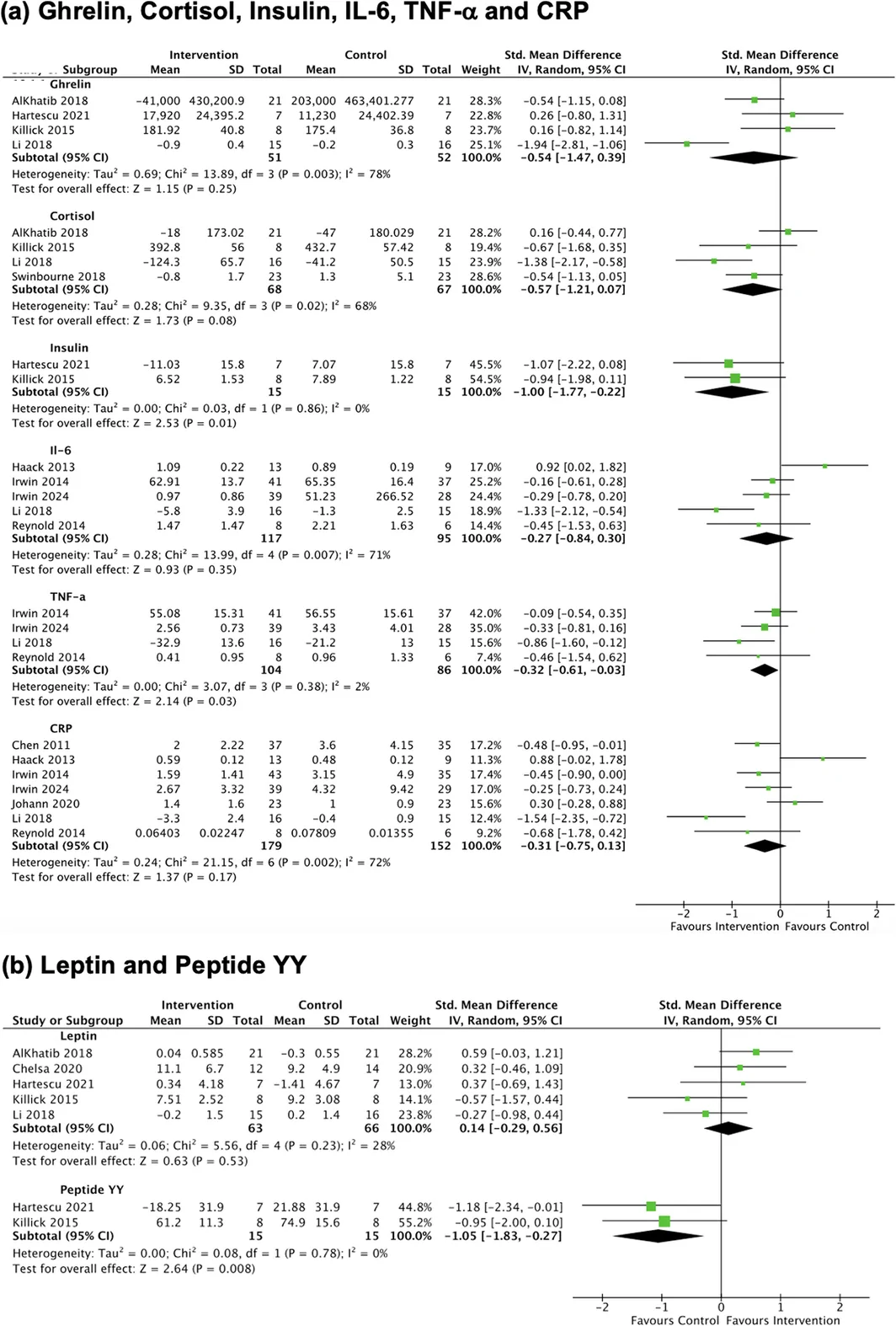
Forrest plots for the results of the meta‐analysis assessing the effect of sleep interventions on hormones regulating or effecting appetite and inflammatory markers using standardized mean differences. Results were calculated using standardized mean differences. CI = confidence interval, CRP = C‐reactive protein, *I* ^2^ = heterogeneity test, IL‐6 = interleukin‐6, IV = inverse variance, SD = standard deviation, TNF‐α = tumor necrosis factor alpha.

Use of sleep interventions improved sleep variables, with reductions in ISI and PSQI global scores averaging 3.02 points (95% CI = −0.05, 6.09, *p* = 0.05, *I* ^2^ for heterogeneity = 81%, *T* ^2^ = 7.92) and 1.47 (0.99, 1.95, *p* < 0.00001, *I* ^2^ = 75%, *T* ^2^ = 0.46), respectively, Appendix D. Improvements in sleep duration and TIB averaged 40.94 min (15.01, 66.88, *p* = 0.002, *I* ^2^ = 97%, *T* ^2^ = 1393.6) and 62.86 min (8.98, 116.75, *p* = 0.02, *I* ^2^ = 96%, *T* ^2^ = 5014.2), respectively.

##### 3.5.2.1 Body Mass Index

When non‐RCTs (*n* = 1) and RCTs (*n* = 12) were combined, considering all sleep interventions, within 13 studies (*n* = 1522, mean baseline BMI = 27.7 kg/m^2^, mean follow‐up = 21.8 weeks), use of the intervention was associated with a nonsignificant reduction in BMI averaging 0.26 kg/m^2^ (−0.70, 0.18, *p* = 0.25, *I* ^2^ = 59%), compared to control (Figure 2). However, on intervention specific analysis, use of CBT‐I and/or sleep hygiene in eight studies (*n* = 1384, mean baseline BMI = 28.4 kg/m^2^, mean follow‐up = 32.4 weeks) led to a statistically significant reduction averaging 0.64 kg/m^2^ (0.28, 1.01, *p* = 0.0006, *I* ^2^ = 16%, *T* ^2^ = 0.05) compared to control. There were no significant changes in five studies (*n* = 138, mean BMI = 26.6 kg/m^2^, mean follow‐up = 4.8 weeks) of sleep extension 0.15 kg/m^2^ (−0.40, 0.11, *p* = 0.26, *I* ^2^ = 0%). The test for subgroup differences between sleep extension and CBT‐I and/or sleep hygiene studies was significant (*p* = 0.0005).

When analyzing BMI outcomes for the RCTs only (*n* = 12), after exclusion of one CBT‐I and/or sleep hygiene study, the same pattern of results were obtained for all studies (*p* = 0.28) and CBT‐I and/or sleep hygiene (*p* = 0.003) compared to control (Figure 2).

In three studies, which included people with T2DM only (*n* = 1121, mean BMI = 27.0 kg/m^2^, mean follow‐up = 30.3 weeks, all RCTs), use of the sleep intervention led to a significant reduction in BMI averaging 0.64 kg/m^2^ (0.37, 0.92, *p* < 0.00001, *I* ^2^ = 0%, *T* ^2^ = 0.0) compared to control (Figure 2).

##### 3.5.2.2 Body Weight Outcomes

When studies of RCTs (*n* = 9) and non‐RCTs (*n* = 2) were combined, use of a sleep intervention was associated with nonsignificant reductions across all interventions in 11 studies (*n* = 493, *p* = 0.23) and individually in CBT‐I and/or sleep hygiene in five studies (*n* = 265, *p* = 0.56) and sleep extension in six studies (*n* = 228, *p* = 0.46) compared to control, Appendix E. The test for subgroup differences between sleep extension and CBT‐I and/or sleep hygiene studies was not significant (*p* = 0.65).

When considering RCTs only (*n* = 9 studies), a similar pattern of results were obtained for all interventions (*p* = 0.23), CBT‐I and/or sleep hygiene (4 studies, *p* = 0.61) or for sleep extension interventions (5 studies, *p* = 0.54) compared to control, Appendix E.

For waist circumference outcomes, in seven studies (*n* = 262, mean length of follow‐up = 25.4 weeks), there were no significant changes from using sleep interventions in any group compared to control: all interventions (*p* = 0.54), CBT‐I and/or sleep hygiene (*p* = 0.55) and sleep extension studies (*p* = 0.81), Appendix E.

For percentage body fat and fat free mass, as only two studies available for each, we analyzed as all sleep interventions; these demonstrated no significant changes (*p* = 0.34 and 0.27, respectively) compared to control, Appendix E.

##### 3.5.2.3 Subgroup Analysis

From three studies (*n* = 78, mean baseline BMI = 32.2 kg/m^2^, mean follow‐up = 14.7 weeks) with baseline mean BMI levels in the obesity category, there was a statistically significant reduction in postintervention BMI levels with use of sleep interventions compared to control, averaging 1.53 kg/m^2^ (0.49, 2.56, *p* = 0.001, *I* ^2^ = 0%, *T* ^2^ = 0.0), Table 2. The results for same analysis for baseline mean BMI levels in either the overweight or healthy categories were not significant (*p* = 0.26 and 0.43, respectively). When body weight was used as the outcome, the postintervention average reductions were significant when the mean baseline BMI levels were in obesity category, averaging 5.55 kg (4.13, 6.97, *p* < 0.00001, *I* ^2^ = 0%, *T* ^2^ = 0.0) in three studies (*n* = 72), and also in overweight category, averaging 0.83 kg (0.32, 1.35, *p* = 0.002, *I* ^2^ = 0%, *T* ^2^ = 0.0) in six studies (*n* = 337), but not within the healthy BMI category (*p* = 0.38). The test for subgroup differences between baseline BMI categories were significant for outcomes of BMI (*p* = 0.007) and body weight outcomes (*p* < 0.00001). Overall, these results suggested a differential impact of sleep interventions according to baseline BMI category.

**TABLE 2:** Pooled changes for outcomes of body weight and BMI according to baseline BMI category of individual studies.

	BMI (kg/m^2^)					Weight (kg)				
BMI category	Number of studies	Number of people	Mean difference (95% CI)	*p*	*I* ^2^ (%)	Number of studies	Number of people	Mean difference (95% CI)	p	*I* ^2^ (%)
Normal healthy range	2	84	0.11 (−0.16, 0.37)	0.43	0	2	84	0.30 (−0.37, 0.97)	0.38	0
Overweight	8	1360	−0.27 (−0.20, 0.74)	0.26	35	6	337	−0.83 (−1.35, −0.32)	**0.001**	**0**
Obesity	3	78	−1.53 (−2.56, −0.49)	**0.001**	0	3	72	−5.55 (−6.97, −4.13)	**< 0.00001**	**0**
No obesity (healthy + overweight combined)	10	1444	−0.12 (−0.56, 0.31)	0.58	59	8	421	−0.29 (−0.99, 0.45)	0.46	24

*Note:* Studies were placed into the BMI categories based on the baseline mean BMI level in each study including ethnic specific cut‐points (reference no 2). Waist circumference was not analyzed here as only 7 studies were available.

Abbreviations: 95% CI = 95% confidence intervals, BMI = body mass index, kg = kilogram, *I* ^2^ = heterogeneity score, m = meter, *n* = number.

An additional subgroup analysis was conducted according to the baseline sleep‐problem phenotype of each study, where three more studies were available, Appendix H. Overall, there were no significant differences between short sleep and insomnia for BMI and body weight outcomes when considering the test for subgroup differences, *p* = 0.86 and 0.99, respectively.

The percentage reduction in BMI between intervention and control for all interventions, CBT‐I and/or sleep hygiene, in baseline obesity and in T2DM were the approximate equivalent of 0.69%, 1.45%, 2.32%, and 0.49%, respectively, but we were unable to test for significance due to the lack of SDs.

#### 3.5.3 Effect of Sleep Interventions on Dietary and Energy Intake Outcomes

Regarding the techniques used for nutritional assessment, three studies employed food record diaries, one study used a dietary recall method and a single study used an objective technique of doubly labeled water method, Appendix F. As only five studies were available in total, we analyzed this outcome as all sleep interventions combined. In the five studies (*n* = 223; 30.9% male, mean duration 13.2 weeks) reporting on daily energy intake, using sleep interventions led to a statistically significant reduction averaging 147.5 cal per day (5.74, 289.41, *I* ^2^ = 53%, *p* = 0.04, *T* ^2^ = 12926.1) compared to control (Figure 3). In three of those studies (*n* = 139; 41.7% males), where the baseline mean BMI levels were in the overweight or obesity ranges, the average reduction was higher at 238.0 cal per day (138.86, 337.22, *I* ^2^ = 0%, *p* < 0.00001, *T* ^2^ = 0.0).

A sensitivity analysis was conducted by removing the single study using an objective nutritional assessment technique (Tasali et al.), to leave the studies employing subjective techniques. This demonstrated the average reduction in daily calorie intake was no longer statistically significant across the remaining four studies (*p* = 0.25) compared to control, but remained significant in the two studies (*n* = 59) with the mean baseline BMI levels were in the overweight or obese ranges, 177.9 cal per day (10.2, 345.6, *I* ^2^ = 0%, *p* = 0.04, *T* ^2^ = 0.0), Appendix F.

Regarding change in dietary composition, only two studies were available, nevertheless there were some relevant findings, Appendix F. Using sleep interventions led to a significant decrease in sugar intake averaging 12.6 g per day (0.06, 25.1, *I* ^2^ = 0%, *p* = 0.05, *T* ^2^ = 0.0) and a significant increase in protein intake as a percentage of daily energy intake, averaging 2.9% (1.14, 4.65, *I* ^2^ = 0%, *p* = 0.001, *T* ^2^ = 0.0) compared to control. There were no significant changes for total energy expenditure (*p* = 0.31), daily intake of sodium (*p* = 0.39), carbohydrate (*p* = 0.40), fiber (*p* = 0.46), saturated (*p* = 0.21), and total fats (*p* = 0.74).

#### 3.5.4 The Effect of Sleep Interventions on Physical Activity and Sedentary Time

For the six outcomes considered, data were available within two to four studies, hence they were analyzed as all sleep interventions. There were no significant changes observed compared to control for total activity (*p* = 0.61), MVPA (*p* = 0.93), light activity (*p* = 0.19), daily step count (*p* = 0.19), sedentary behavior (*p* = 0.69), and sitting time (*p* = 0.25), Appendix G.

#### 3.5.5 Secondary Analysis on Hormonal and Inflammatory Biomarkers

Five hormones regulating appetite or potentially affecting weight were analyzed using standardized mean differences in two to five studies, typically with smaller sample sizes of 30–129 people (Figure 4). Use of a sleep interventions led to significant reductions in levels of peptide YY and insulin in two studies (both with *n* = 30), averaging 1.05 (0.27, 1.83, *I* ^2^ = 0%, *p* = 0.008, *T* ^2^ = 0.0) and 1.00 (0.22, 1.77, *I* ^2^ = 0%, *p* = 0.01, *T* ^2^ = 0.0), respectively, compared to control. There were nonsignificant decreases in levels of Ghrelin (*p* = 0.20) and cortisol (*p* = 0.08) and an increase in leptin (*p* = 0.19). For the inflammatory biomarkers examined, using sleep interventions led to a significant reduction in tumor necrosis factor alpha, averaging 0.32 (0.03, 0.61, *I* ^2^ = 2%, *p* = 0.03, *T* ^2^ = 0.0) in four studies (*n* = 190), but nonsignificant reductions in C‐reactive protein (*p* = 0.17) and interleukin‐6 (*p* = 0.35) in seven and five studies.

##### 3.5.5.1 Sensitivity Analyses

For the risk of publication bias, there was no visible asymmetry on visual inspection of funnel plots for outcomes of BMI and body weight before and after the removal of smaller studies, Appendix I. This was complemented by nonsignificant Egger's regression–based tests for both BMI and body weight (*p* = 0.157 and *p* = 0.086). After removal of smaller studies, only two studies remained for in each outcome, which was not sufficient for calculating an Egger's regression–based test, Appendix I. Overall, the risk of publication bias was considered low. Secondly, a “leave‐one‐out analysis” did not change the overall significance of results for BMI and body weight, suggesting there were no outlier studies influencing results (all *p* values > 0.05), Appendix J. Thirdly, when conducting a subgroup analysis of the meta‐analyses results according to the risk of bias categories of low risk and some concern, the test for subgroup differences was not significant for either BMI (*p* = 0.25) or body weight (*p* = 0.78) outcomes, Appendix K. Finally, removal of studies containing self‐reported weight and height measures (*n* = 1) did not change the significance for weight or BMI outcomes, Appendix L.

##### 3.5.5.2 Meta‐Regression Analysis

As heterogeneity was considered as moderate to high for some outcomes, meta‐regression models were performed. These demonstrated the type of intervention and baseline BMI category to be significant confounders for the outcomes of body weight and BMI, Table 3 and Appendix M. Specifically, studies using CBT‐I and/or sleep hygiene were found to have a larger effect size than those using sleep extension, by a mean BMI of 0.81 kg/m^2^ (0.45, 1.17, *p* < 0.001) and body weight of 4.29 kg (95% CI = 2.00, 6.50, *p* < 0.001). Furthermore, studies with a baseline BMI in the obesity category were found to have a larger effect size than those with BMI in the nonobesity category (i.e., healthy or overweight), by a postintervention mean BMI of 1.32 kg/m^2^ (0.02, 2.61, *p* = 0.046) and body weight of 5.26 kg (95% CI = 3.32, 7.20, *p* < 0.001). Similarly, when considering baseline BMI as a continuous variable, higher levels were found to have a significantly larger effect size, where the benefit of the intervention on BMI per every 1 kg/m^2^ increase at baseline, decreased the postintervention body weight by 0.40 kg (95% CI = 0.02, 0.79, *p* = 0.040). These models may suggest the impact of the interventions were largely confined to those who underwent CBT‐I and/or sleep hygiene or with higher baseline BMI levels, especially in the obesity category.

**TABLE 3:** Results of the meta‐regression analysis of the effects of sleep interventions on anthropometric markers.

	BMI (kg/m^2^)			Body weight (kg)		
Variable	No. of studies *	Coefficient (95% CI)	p	No. of studies *	Coefficient (95% CI)	No. of
Age (per decade)	13	−0.12 (−0.42, 0.18)	0.43	11	−1.15 (−2.55, 0.25)	0.11
Proportion of males (10 pp)	13	0.13 (−0.19, 0.45)	0.43	11	0.28 (−0.85, 1.40)	0.63
Geographical location (Europe vs. other)	13	−0.18 (−1.15, 0.80)	0.72	11	−1.50 (−7.16, 4.15)	0.60
Study design (RCT vs. other)	13	0.35 (−3.47, 2.77)	0.83	11	1.24 (−5.46, 7.94)	0.72
Intervention (CBT‐I/hygiene vs. extension)	13	−0.81 (−1.17, −0.45)	**< 0.001**	11	−4.29 (−6.50, −2.00)	**< 0.001**
Intervention delivery (face‐to‐face vs. remote/digital)	13	0.42 (−0.69, 1.54)	0.46	11	4.26 (2.00, 6.51)	**< 0.001**
Length of follow‐up (month)	13	−0.01 (−0.12, 0.09)	0.81	11	−0.13 (−0.63, 0.88)	0.75
Year of publication (per decade)	13	−1.59 (−3.07, −0.11)	**0.035**	12	−6.50 (−13.30, 0.31)	0.061
Baseline BMI category (obese vs. nonobese)	13	−1.32 (−2.61, −0.02)	**0.046**	11	−5.26 (−7.20, −3.32)	**< 0.001**
Baseline BMI (per 1 kg/m^2^)	13	−0.10 (−0.22, 0.02)	0.090	11	−0.40 (−0.79, −0.02)	**0.040**
T2DM personal history (yes vs. no)	8	−0.11 (−0.63, 1.16)	0.56	6	−3.85 (−18.3, 10.6)	0.60
Baseline PSQI global score	9	−0.11 (−0.41, 0.18)	0.45	7	−0.17 (−2.04, 1.69)	0.86
Insomnia Severity Index	3	−0.19 (−0.98, 0.60)	0.64	2	−0.49 (−3.44, 2.46)	0.74

*Note:* Results are from univariable meta‐regression analyses, with separate models produced for each variable. The dependent variable was the mean difference in weight and BMI outcomes between the intervention and control groups; negative values indicate a lower level in the intervention arm. For binary variables, the coefficient represents the difference in the effect size between the two subgroups of studies. For continuous variables, the coefficient represents the change in the effect size per the stated number of units increase in the factor. Bold *p* values are significant at *p* < 0.05.

* The number of studies included in the analysis, after excluding those with missing data for the variable of interest.

** The coefficient represents the change the effect size per 10 percentage point increase in the proportion of males in the study (e.g., for a studies comprising 40% vs. 30% males).

Abbreviations: 95% CI = 95% confidence intervals, BMI = body mass index, CBT‐I = cognitive behavior therapy for insomnia, kg = kilogram, *n* = number, pp = percentage point, PSQI = Pittsburgh Sleep Quality Index, RCT = randomized controlled trial, T2DM = type 2 diabetes mellitus.

In the GRADE analysis, for the outcomes of BMI, body weight, and daily calorie intake, the final levels of certainty determined were moderate, low, and moderate, respectively, Appendix N.

## 4 Discussion

This systematic review and meta‐analysis is the first to demonstrate that using nonpharmacological behavioral sleep interventions decreases anthropometric measures of obesity in adults with poor sleep health, but without OSA. Specifically, there were reductions in BMI of 1.5 kg/m^2^ in obesity, 0.63 kg/m^2^ using CBT‐I and/or sleep hygiene, and 0.64 kg/m^2^ in type 2 diabetes. There were no significant results for sleep extension. Across all sleep interventions, which improved sleep index scores on the ISI and PSQI global by 3.0 and 1.5, body weight decreased in the obesity and overweight ranges by 5.6 and 0.8 kg, respectively. However, heterogeneity was moderate to high for some, but not all outcomes, which somewhat reduces the generalizability of the findings. Through the meta‐regression analysis, the BMI category and type of sleep of intervention were found to be significant confounders for both BMI and body weight outcomes. Considering lifestyle habits, sleep interventions were associated with daily dietary changes, which favored weight loss, including a reduced energy intake of 147 cal, higher at 238 cal if the baseline BMI was above the healthy category, increased protein and decreased sugar intake. In contrast, physical activity and sedentary time did not change.

Multiple epidemiological studies associate short sleep duration with weight gain and obesity. However, we found sleep extension by itself was not associated with any change in obesity markers, despite improving TIB and sleep duration by 41 and 63 min, respectively. While this may represent a true effect, we should consider that sleep extension studies were conducted over shorter follow‐up time frames, ranging from 2 weeks to 2.7 months, suggesting longer studies may be required to see changes on anthropometric markers. Furthermore, no sleep interventions led to changes in waist circumference, a structural measure related to abdominal obesity, from which we should draw similar conclusions.

As sleep interventions have demonstrated reductions in some anthropometric measures, as well as in glucose and blood pressure, we wished to explore more about how they may be producing these effects [11, 12]. We found using a sleep intervention reduced energy intake by 147 cal per day, although higher in obesity/overweight at 238 cal per day, suggesting some of the increased daily energy intake of 204–385 cal associated with poor sleep can be meliorated [15, 16]. Secondly, previous research has demonstrated sleep restriction to less than 5.5 h can alter dietary composition by increasing daily fat intake; also, improving sleep health decreases the desire for sweet and salty foods by 62% and appetite by 14% [15, 16, 57]. Our results extend these findings by suggesting using sleep interventions can alter daily dietary composition with lower sugar and higher protein intake. Finally, there was insufficient data available to examine the impact of sleep interventions on the change of meal timings, where an evening chronotype is associated with higher weight gain [58]. Although sleep hygiene recommendations are not standardized between international committees, they can include advice to avoid main meals after 7:00 p.m. (19:00 h) or 2 h before bedtime, which may help prevent night‐time snacking/meals. One study that provided this specific advice to people with an evening chronotype, as part of sleep hygiene, demonstrated weight loss results of 7.3 kg compared to control, which was the highest of studies examined [34].

In contrast to dietary outcomes, we found no evidence that improving sleep increases physical activity of various intensities, step count, or reduces sedentary time. While this could reflect limited data available, similar null findings for most OSA studies could suggest a true effect in the context of improving sleep disorders [59, 60, 61]. Increasing physical activity, however, is considered a complex behavior change dependent on overcoming barriers such as the stage of readiness to perform more activity and fear of movement [17]. Although complementing behavioral sleep interventions with focused physical activity counseling has been proposed, trials reported no changes in activity when testing this [17, 33, 62]. This challenges the previously suggested bidirectional relationship between poor sleep and physical activity, indicating that more research is needed to clarify this association.

Poor sleep is associated with hormonal and inflammatory changes that can be associated with weight gain and cardiometabolic diseases [9]. Our analysis of hormones regulating appetite or potentially affecting weight was based on smaller numbers of people, hence although most results were not significant and had relatively wide 95% confidence intervals, the direction of changes following sleep interventions in leptin, ghrelin, cortisol, and insulin would tend to favor weight loss. A similar nonsignificant reduction in HOMA‐IR was reported in another meta‐analysis [11]. In contrast, for Peptide YY, which increases satiety, the observed decrease could favor weight gain, although this may also represent a neurohormonal adaptation after a period of weight loss. From the three markers of inflammation, there was a trend of the intervention leading to a reduction of the markers, but this was only significant for tumor necrosis factor alpha.

Overall, it remains unclear whether the reductions on CVD risk factors brought about by using a sleep intervention are due to the direct impact on hormonal/biomarker levels or the indirect effects on lifestyle changes, or a combination of these.

Comparing our results to other interventions, meta‐analyses of CPAP use in OSA demonstrate a slight increase in BMI of 0.148 kg/m^2^ (95% CI = 0.04, 0.26) [63]. Secondly, meta‐analyses of general CBT demonstrate a nonsignificant BMI reduction of 0.12 kg/m^2^ (−0.50, 0.12) and a weight loss of 1.70 kg (−2.52, −0.86) [64, 65]. Thirdly, a network meta‐analysis of weight loss pharmacotherapies demonstrated 12‐month results for liraglutide, naltrexone‐bupropion, lorcaserin, and orlistat ranging between 2.6 and 5.3 kg [66]. Although the results of the present meta‐analysis study were based on shorter time frames, a similar weight loss was achieved for those studies in the obesity range.

### 4.1 Limitations and Strengths

Firstly, some studies were of variable quality, as described in the quality assessment, which reflect the available data at the time. Secondly, we were unable to account for some important outcome measures related to cardiometabolic health because they were less commonly or not measured. This included percentage body fat, visceral fat, and also total energy expenditure for diet, possibly because they require more expensive equipment, time, and expertise for their use. The most reported outcomes in our meta‐analysis include those used for current clinical decision making in weight management practice, especially BMI [2]. Thirdly, many studies did not report on use of weight loss medications. The most efficacious weight loss medications, glucagon‐like peptide‐1 receptors agonists (GLP‐1RAs), have been mainly licensed for use in T2DM with the presence of obesity [67, 68]. Only three out of 27 included studies consisted of populations of T2DM where the mean BMI in each of these studies was below the obesity range [35, 41, 43]. Other studies excluded people on weight loss medications at screening [34]. Finally, for the subgroup analysis according to BMI categories, we used ethnic specific cut‐points according to the study's country of origin, which may be less accurate in countries with multiethnic populations such as the UK and USA, depending on the uptake to research of ethnic minority groups in those countries/studies.

The strengths of this study are the systematic approach and robust methods used to obtain our results, ensuring that they met accepted standards for meta‐analyses. We considered a wide range of sleep behaviors as causes for poor sleep, as well as measures for obesity, diet, energy intake, and activity, which were used in the comprehensive list of search terms in the search strategy. We analyzed full‐text articles and abstracts for studies in all languages, allowing us to detect as many eligible studies as possible. In addition, we contacted the lead authors of 31 studies on up to two occasions to obtain further outcome data on studies relevant to this analysis. Overall, the studies incorporated a broad range of adult age, sex, sleep behaviors, and BMI categories suggesting the results are applicable to a wide variety of people.

### 4.2 Potential Implications for Policy and Clinical Practice

Addressing poor sleep health does not routinely feature in international obesity management guidelines with suggestions there is insufficient evidence to support introducing sleep‐focused interventions into clinical weight‐management programs [69]. The present study provides some preliminary evidence for the benefits of addressing poor sleep health using CBT‐I and/or sleep hygiene, especially in the obesity range and in T2DM. Additionally, targeting poor sleep health may have other benefits. Weight‐loss interventions including dietary modification and bariatric surgery appear to be less effective in the presence of poor sleep health, as the initial weight loss initially is lower and also in those who manage to successfully lose weight initially, the weight regain is higher [70, 71, 72, 73, 74, 75, 76]. This identifies poor sleep health as a barrier towards effective weight loss and/or maintenance compared to those with adequate sleep. Similarly, physical activity interventions may be less effective or have fewer benefits in poor compared to healthy sleep [17, 77]. Secondly, two recent meta‐analyses found using behavioral sleep interventions led to clinically significant reductions of other CVD risk factors including glycaemia and blood pressure [11, 12]. This included a lowering of glycated hemoglobin, HbA1c, of 0.43% in T2DM, through using CBT‐I and/or sleep hygiene and systolic and diastolic blood pressure by 5 and 2.9 mmHg from all sleep interventions, respectively. As people living with obesity are also at risk of T2DM and CVD, it could be proposed that detecting poor sleep, followed by implementing interventions to improve their sleep, may be a cost‐effective approach to reducing risk of obesity‐related complications. Thus, optimizing sleep health could be incorporated into existing public health strategies as part of personalized care. For examples, lifestyle programs designed for weight loss or T2DM prevention could feature modules on sleep improvement through behavior sleep interventions. Poor sleep health can be assessed through using risk scores such as the ISI and PSQI, while many modern‐day smart wristwatch devices can measure sleep tracking for time‐in‐bed [20]. CBT‐I and sleep extension interventions can be delivered via a healthcare professional in person or remotely, or self‐implemented using mobile phone/internet applications. Sleep pharmacotherapies (e.g., benzodiazepine receptor agonists or melatonin agonists) may temporarily improve poor sleep health in some cases but risk side effects, dependency issues and are usually not considered a long‐term solution. Behavioral sleep interventions may be a more favorable option as they do not require taking tablets and may have a greater longer‐term efficacy than pharmacotherapies [78].

Finally, several efficacious weight loss pharmacotherapies have been developed over the last few decades, particularly GLP‐1RAs. These medications, however, can have high discontinuation rates of up to two‐thirds after 12 months, as well as issues of side effects, accessibility and economic implications for many health care systems globally [79, 80]. Furthermore, bariatric surgery, although effective for weight loss, is underutilized, often difficult to access, requires patients to be fit for anesthesia and may not be the preferable option for patients [81]. Thus, lifestyle measures have often been the focus of treatment for many people living with obesity; however, dietary modification and physical activity recommendations themselves are not always easy to achieve or maintain in the long‐term. This suggests a role for addressing poor sleep health as part of personalized lifestyle interventions.

In conclusion, use of behavioral sleep interventions led to significant reductions in BMI and body weight in adults with poor sleep health, particularly in obesity range. Future research should examine the value of using behavioral sleep interventions in adults with poor sleep health in large scale RCTs using standardized sleep protocols and with longer follow‐up periods. This will determine if addressing poor sleep health can be used as a potential target to aid current measures of preventing or treating obesity and T2DM.

## Author Contributions

**Samiul A. Mostafa:** conceptualization (lead), data curation, formal analysis (lead), investigation (equal), methodology (lead), project administration, resources, supervision (equal), validation, visualization, writing – original draft. **Pushpa Singh:** investigation (equal), formal analysis (supporting), validation, writing – review and editing (equal). **Feaz Babwah:** investigation (equal), validation, writing – review and editing (equal). **Wasim Hanif:** conceptualization (supporting), methodology (supporting), writing – review and editing (equal). **George Balanos:** conceptualization (supporting), methodology (supporting), writing – review and editing (equal). **Krishnarajah Nirantharakumar:** conceptualization (supporting), methodology (supporting), writing – review and editing (equal). **Jason G. Ellis:** conceptualization (supporting), methodology (supporting), writing – review and editing (equal). **Abd A. Tahrani:** conceptualization (supporting), methodology (supporting), supervision (equal), writing – review and editing (equal).

## Conflicts of Interest

S.M. undertook this research work through receiving a Clinical Research Network West Midlands Health and Care Research Scholarship. He has previously received research support from Novo Nordisk Research Foundation UK and Academy of Medical Sciences.

A.A.T. is currently an employee and share‐holder of Amagen Research Copenhagen. Amagen Research Copenhagen had no role in this manuscript. The views expressed in this manuscript are those of the authors and not Amagen.

The other authors declare no conflicts of interest.

## Supporting information


**Data S1:** Supplementary Information.

## Data Availability

Data used in this study (including an unpublished protocol) are retained by the research team. Some of the data accumulated for the purposes of this study was requested via contacting the lead authors of the individual studies, as the data were not publicly available. We did not seek permissions with these lead authors to share their data within the public domain or with other research teams.
