# Supplementary material for: The Effect of Behavioral Sleep Interventions on Measures of Obesity, Dietary Intake and Physical Activity in Adults With Poor Sleep Health: A Systematic Review, Meta‐Analysis and Meta‐Regression Analysis
Source: Obes Rev. 2026 Apr 27;27(11):e70139. doi: 10.1111/obr.70139 (PMC13655966; doi:10.1111/obr.70139)
Supplement: Supplementary file 1 [file OBR-27-e70139-s001.pdf]

Supplementary material:

The effect of behavioural sleep interventions on measures of obesity, dietary intake and physical activity in adults with poor sleep health: a systematic review, meta-analysis and meta-regression analysis.

Samiul A Mostafa, Pushpa Singh, Feaz Babwah, Wasim Hanif, George Balanos, Krishnarajah Nirantharakumar, Jason G. Ellis, Abd A Tahrani

All authors: University of Birmingham, Birmingham, UK, except Professor Ellis: University of Northumbria, UK.

Correspondence to Dr. Samiul A Mostafa. Email: s.a.mostafa@bham.ac.uk and samiul.mostafa@uhb.nhs.uk, Tel: +44 (0)121 371 4737; Fax: +44 (0)121 460 5837

CONTENTS:

Appendix A. Database search strategies for bibliographical databases

Appendix B. Table for study entry eligibility criteria.

Appendix C. Risk of bias assessment

Appendix D. Forrest plots: effect of sleep interventions on sleep variables

Appendix E. Forrest plots: effect of sleep interventions on (a) body weight (b) waist circumference, (c) percentage body fat and (d) fat free mass.

Appendix F. Forrest plots: effect of sleep interventions on dietary intake

Appendix G. Forrest plots: effect of sleep interventions on physical activity and sedentary behaviour

Appendix H. Sub-analysis: Forrest plots for BMI and body weight according to baseline sleep-problem phenotype.

Appendix I. Sensitivity analysis: Risk of publication bias for (a) BMI, (b) body weight and (c) daily calorie intake.

Appendix J. Sensitivity analysis: ‘leave one-out-analysis’

Appendix K. Sensitivity analysis: Forrest plots: effect of sleep interventions on (a) BMI and (b) body weight according to the risk of bias category

Appendix L. Sensitivity analysis: Forrest plots after removal of studies featuring self-measured height and weight.

Appendix M. Bubble plots: association of baseline BMI level and outcomes of post-intervention change in (a) BMI level and (b) body weight

Appendix N. GRADE assessment summary of findings

Appendix A: Database search strategies for bibliographical databases

1) MEDLINE.

Ovid MEDLINE(R) ALL <1946 to April 14, 2025>

1 Chronotype.ab. or Chronotype.ti.

2 (Circadian adj1 (rhythm or misalignment or clock*)).ab. or (Circadian adj1 (rhythm or misalignment or clock*)).ti.

3 (Morning* adj2 Evening*).ab. or (Morning* adj2 Evening*).ti.

4 "Biological Clock*".ab. or "Biological Clock*".ti.

5 "Social jetlag".ab. or "Social jetlag".ti.

6 (Sleep adj1 (duration or disorder* or "Initiation and Maintenance Disorder" or stage* or "Apn?ea Syndrome*" or "Apn?ea Obstructive" or "wake disorder*" or "disordered breathing" or DYSFUNTION or Problem*)).ab. or (Sleep adj1 (duration or disorder* or "Initiation and Maintenance Disorder" or stage* or "Apn?ea Syndrome*" or "Apn?ea Obstructive" or "wake disorder*" or "disordered breathing" or DYSFUNTION or Problem*)).ti.

7 (Nap or naps or napping).ab. or (Nap or naps or napping).ti.

8 wakefulness.ab. or wakefulness.ti.

9 "shift work*".ab. or "shift work*".ti.

10 insomnia.ab. or insomnia.ti.

11 hyposomni*.ab. or hyposomni*.ti.

12 sleepless.ab. or sleepless.ti.

13 (early adj1 (awake* or wake or wakes or waking)).ab. or (early adj1 (awake* or wake or wakes or waking)).ti.

14 ((difficult* or disturb* or inabilit* or unable* or problem* or reduced) adj3 (asleep or sleep*)).ab. or ((difficult* or disturb* or inabilit* or unable* or problem* or reduced) adj3 (asleep or sleep*)).ti.

15 exp sleep deprivation/ or exp sleep disorders, circadian rhythm/ or exp sleep disorders, intrinsic/ 89695 16 exp Circadian Rhythm/

17 exp Sleep Stages/

18 1 or 2 or 3 or 4 or 5 or 6 or 7 or 8 or 9 or 10 or 11 or 12 or 13 or 14 or 15 or 16 or 17 281369

19 "cognitive behavio?ral therap*".ab. or "cognitive behavio?ral therap*".ti.

20 "cognitive therap*".ab. or "cognitive therap*".ti.

21 (Sleep adj1 (hygiene or education or program* or advice or extension)).ab. or (Sleep adj1 (hygiene or education or program* or advice or extension)).ti.

22 "bright light".ab. or "bright light".ti.

23 "stimulus control".ab. or "stimulus control".ti.

24 relaxation.ab. or relaxation.ti.

25 meditation.ab. or meditation.ti.

26 mindfulness.ab. or mindfulness.ti.

27 non-pharmacological.ab. or non-pharmacological.ti.

28 exp Cognitive Behavioral Therapy/

29 Sleep Hygiene/

30 meditation/ or relaxation therapy/

31 Mindfulness/

32 19 or 20 or 21 or 22 or 23 or 24 or 25 or 26 or 27 or 28 or 29 or 30 or 31

33 Weight.ab. or weight.ti.

34 "body mass index".ab. or "body mass index".ti.

35 ("body fat" adj1 percentage*).ab. or ("body fat" adj1 percentage*).ti.

36 "waist to hip ratio".ab. or "waist to hip ratio".ti.

37 "waist to height ratio".ab. or "waist to height ratio".ti.

38 (anthropometric adj2 (measure* OR indicator*)).ab. OR (anthropometric ADJ2 (measure*" or indicator*)).ti.

39 "Body Shape Index".ab. or "Body Shape Index".ti.

40 "fat mass index".ab. or "fat mass index".ti.

41 (Circumference adj1 (Waist or arm or neck)).ab. or (Circumference adj1 (Waist or arm or neck)).ti. 44196 42 exp "body weights and measures"/ or body mass index/

43 "Average Density".ab. or "Average Density".ti.

44 "Body Volume Index".ab. or "Body Volume Index".ti.

45 "Body Roundness Index".ab. or "Body Roundness Index".ti.

46 ((Obesity or obese or adiposity) adj1 Marker*).ab. or ((Obesity or obese or adiposity) adj1 Marker*).ti.

47 ((energy or Macronutrient or Carbohydrate or PROTEIN or fat) adj1 (expenditure or intake or density or balance)).ab. or ((energy or Macronutrient or Carbohydrate or PROTEIN or fat) adj1 (expenditure or intake or density or balance)).ti.

48 "portion size*".ab. or "portion size*".ti.

49 "food reformulation".ab. or "food reformulation".ti.

50 (calorie* or food intake or appetite or eating).ab. or (calorie* or food intake or appetite or eating).ti.

51 ("Basal Metabolic Rate" or BMR).ab. or ("Basal Metabolic Rate" or BMR).ti.

52 exp Basal Metabolism/

53 "Resting Metabolic Rate".ab. or "Resting Metabolic Rate".ti.

54 "Diet Induced Thermogenesis".ab. or "Diet Induced Thermogenesis".ti.

55 "physical activit*".ab. or "physical activit*".ti.

56 "non-exercise activity thermogenesis".ab. or "non-exercise activity thermogenesis".ti. 81

57 (sedentary adj1 (TIME or behavio?r or lifestyle)).ab. or (sedentary adj1 (TIME or behavio?r or lifestyle)).ti.

58 Sedentary Behavior/

59 33 or 34 or 35 or 36 or 37 or 38 or 39 or 40 or 41 or 42 or 43 or 44 or 45 or 46 or 47 or 48 or 49 or 50 or 51 or 52 or 53 or 54 or 55 or 56 or 57 or 58

60 18 and 32 and 59

69 18 and 32 and 68

2. Embase

<1974 to 2024 April 14>

1 Chronotype.ab. or Chronotype.ti.

2 (Circadian adj1 (rhythm or misalignment or clock*)).ab. or (Circadian adj1 (rhythm or misalignment or clock*)).ti.

3 (Morning* adj2 Evening*).ab. or (Morning* adj2 Evening*).ti.

4 "Biological Clock*".ab. or "Biological Clock*".ti.

5 "Social jetlag".ab. or "Social jetlag".ti.

6 (Sleep adj1 (duration or disorder* or "Initiation and Maintenance Disorder" or stage* or "Apn?ea Syndrome*" or "Apn?ea Obstructive" or "wake disorder*" or "disordered breathing" or DYSFUNTION or Problem*)).ab. or (Sleep adj1 (duration or disorder* or "Initiation and Maintenance Disorder" or stage* or "Apn?ea Syndrome*" or "Apn?ea Obstructive" or "wake disorder*" or "disordered breathing" or DYSFUNTION or Problem*)).ti.

7 (Nap or naps or napping).ab. or (Nap or naps or napping).ti.

8 wakefulness.ab. or wakefulness.ti.

9 "shift work*".ab. or "shift work*".ti.

10 insomnia.ab. or insomnia.ti.

11 hyposomni*.ab. or hyposomni*.ti.

12 sleepless.ab. or sleepless.ti.

13 (early adj1 (awake* or wake or wakes or waking)).ab. or (early adj1 (awake* or wake or wakes or waking)).ti.

14 ((difficult* or disturb* or inabilit* or unable* or problem* or reduced) adj3 (asleep or sleep*)).ab. or ((difficult* or disturb* or inabilit* or unable* or problem* or reduced) adj3 (asleep or sleep*)).ti.

15 "cognitive behavio?ral therap*".ab. or "cognitive behavio?ral therap*".ti. 33513

16 "cognitive therap*".ab. or "cognitive therap*".ti.

17 (Sleep adj1 (hygiene or education or program* or advice or extension)).ab. or (Sleep adj1 (hygiene or education or program* or advice or extension)).ti.

18 "bright light".ab. or "bright light".ti.

19 "stimulus control".ab. or "stimulus control".ti.

20 relaxtion.ab. or relaxtion.ti.

21 meditation.ab. or meditation.ti.

22 mindfulness.ab. or mindfulness.ti.

23 non-pharmacological.ab. or non-pharmacological.ti.

24 Weight.ab. or weight.ti.

25 "body mass index".ab. or "body mass index".ti.

26 ("body fat" adj1 percentage*).ab. or ("body fat" adj1 percentage*).ti.

27 "waist to hip ratio".ab. or "waist to hip ratio".ti.

28 "waist to height ratio".ab. or "waist to height ratio".ti.

29 (anthropometric adj2 ("measure* OR indicator*)).ab. OR (anthropometric ADJ2 (measure*" or indicator*)).ti.

30 "Body Shape Index".ab. or "Body Shape Index".ti.

31 "fat mass index".ab. or "fat mass index".ti.

32 (Circumference adj1 (Waist or arm or neck)).ab. or (Circumference adj1 (Waist or arm or neck)).ti.

33 "Average Density".ab. or "Average Density".ti.

34 "Body Volume Index".ab. or "Body Volume Index".ti.

35 "Body Roundness Index".ab. or "Body Roundness Index".ti.

36 ((Obesity or obese or adiposity) adj1 Marker*).ab. or ((Obesity or obese or adiposity) adj1 Marker*).ti.

37 exp sleep stage/

38 exp circadian rhythm/

39 exp sleep deprivation/

40 exp sleep disorder/

41 1 or 2 or 3 or 4 or 5 or 6 or 7 or 8 or 9 or 10 or 11 or 12 or 13 or 14 or 37 or 38 or 39 or 40

42 exp cognitive therapy/

43 sleep hygiene/

44 exp meditation/

45 relaxation training/

46 exp mindfulness/

47 15 or 16 or 17 or 18 or 19 or 20 or 21 or 22 or 23 or 42 or 43 or 44 or 45 or 46

48 body mass/ or anthropometric parameters/

49 ((energy or Macronutrient or Carbohydrate or PROTEIN or fat) adj1 (expenditure or intake or density or balance)).ab. or ((energy or Macronutrient or Carbohydrate or PROTEIN or fat) adj1 (expenditure or intake or density or balance)).ti.

50 "portion size*".ab. or "portion size*".ti.

51 "food reformulation".ab. or "food reformulation".ti.

52 (calorie* or food intake or appetite or eating).ab. or (calorie* or food intake or appetite or eating).ti.

53 ("Basal Metabolic Rate" or BMR).ab. or ("Basal Metabolic Rate" or BMR).ti.

54 exp basal metabolic rate/

55 "Resting Metabolic Rate".ab. or "Resting Metabolic Rate".ti.

56 "Diet Induced Thermogenesis".ab. or "Diet Induced Thermogenesis".ti.

57 "physical activit*".ab. or "physical activit*".ti.

58 "non-exercise activity thermogenesis".ab. or "non-exercise activity thermogenesis".ti.

59 (sedentary adj1 (TIME or behavio?r or lifestyle)).ab. or (sedentary adj1 (TIME or behavio?r or lifestyle)).ti.

60 sedentary lifestyle/

61 24 or 25 or 26 or 27 or 28 or 29 or 30 or 31 or 32 or 33 or 34 or 35 or 36 or 48 or 49 or 50 or 51 or 52 or 53 or 54 or 55 or 56 or 57 or 58 or 59 or 60

62 41 and 47 and 61

3) CINAHL

#	Query
S64	S18 AND S32 AND S63
S63	S33 OR S34 OR S35 OR S36 OR S37 OR S38 OR S39 OR S40 OR S41 OR S42 OR S43 OR S44 OR S45 OR S46 OR S47 OR S48 OR S49 OR S50 OR S51 OR S52 OR S53 OR S54 OR S55 OR S56 OR S57 OR S58 OR S59 OR S60 OR S61 OR S62
S62	(MH "Sedentary Behavior")
S61	XB ((sedentary N1 (TIME or behavio#r or lifestyle)))
S60	XB "non-exercise activity thermogenesis"
S59	XB "physical activit*"
S58	XB "Diet Induced Thermogenesis"
S57	XB "Resting Metabolic Rate"
S56	(MH "Basal Metabolism")

S55	XB ("Basal Metabolic Rate" or BMR)
S54	XB (calorie* or food intake or appetite or eating)
S53	XB "food reformulation"
S52	XB "portion size*"
S51	XB (((energy or Macronutrient or Carbohydrate or PROTEIN or fat) N1 (expenditure or intake or density or balance)))
S50	XB (((Obesity or obese or adiposity) N1 Marker*).)
S49	XB "Body Roundness Index"
S48	XB "Body Volume Index"
S47	XB "Average Density"
S46	(MH "Body Weights and Measures+")
S45	TI (((Obesity or obese or adiposity) n1 Marker*)) OR AB (((Obesity or obese or adiposity) n1 Marker*))
S44	TI "Body Roundness Index" OR AB "Body Roundness Index"
S43	TI "Body Volume Index" OR AB "Body Volume Index"
S42	TI "Average Density" OR AB "Average Density"
S41	TI ((Circumference n1 (Waist or arm or neck))) OR AB ((Circumference n1 (Waist or arm or neck)))
S40	TI "fat mass index" OR AB "fat mass index"
S39	TI "Body Shape Index" OR AB "Body Shape Index"
S38	TI ((anthropometric n2 ("measure* OR indicator*))) OR AB ((anthropometric n2 ("measure* OR indicator*)))
S37	TI "waist to height ratio" OR AB "waist to height ratio"
S36	TI "waist to hip ratio" OR AB "waist to hip ratio"
S35	TI ("body fat" N1 percentage*) OR AB ("body fat" N1 percentage*)
S34	TI "body mass index" OR AB "body mass index"
S33	TI Weight OR AB Weight
S32	S19 OR S20 OR S21 OR S22 OR S23 OR S24 OR S25 OR S26 OR S27 OR S28 OR S29 OR S30 OR S31
S31	(MH "Mindfulness+")
S30	(MH "Relaxation") OR (MH "Relaxation Techniques+")
S29	(MH "Sleep Hygiene+")
S28	(MH "Cognitive Therapy+")

S27	TI non-pharmacological OR AB non-pharmacological
S26	TI mindfulness OR AB mindfulness
S25	TI meditation OR AB meditation
S24	TI relaxation OR AB relaxation
S23	TI "stimulus control" OR AB "stimulus control"
S22	TI "bright light" OR AB "bright light"
S21	TI ((Sleep N1 (hygiene or education or program* or advice or extension))) OR AB ((Sleep N1 (hygiene or education or program* or advice or extension)))
S20	TI "cognitive therap*" OR AB "cognitive therap*"
S19	TI "cognitive behavio#ral therap*" OR AB "cognitive behavio#ral therap*"
S18	S1 OR S2 OR S3 OR S4 OR S5 OR S6 OR S7 OR S8 OR S9 OR S10 OR S11 OR S12 OR S13 OR S14 OR S15 OR S16 OR S17
S17	(MH "Sleep Stages+")
S16	(MH "Circadian Rhythm+")
S15	(MH "Sleep Deprivation") OR (MH "Sleep Disorders, Circadian Rhythm+") OR (MH "Sleep Disorders, Intrinsic+")
S14	TI (((difficult* or disturb* or inabilit* or unable* or problem* or reduced) N3 (asleep or sleep*))) OR AB (((difficult* or disturb* or inabilit* or unable* or problem* or reduced) N3 (asleep or sleep*)))
S13	TI ((early N1 (awake* or wake or wakes or waking))) OR AB ((early N1 (awake* or wake or wakes or waking)))
S12	TI sleepless OR AB sleepless
S11	TI hyposomni* OR AB hyposomni*
S10	TI insomnia OR AB insomnia
S9	TI "shift work*" OR AB "shift work*"
S8	TI wakefulness OR AB wakefulness
S7	TI (Nap or naps or napping) OR AB (Nap or naps or napping)
S6	TI ((Sleep N1 (duration or disorder* or "Initiation and Maintenance Disorder" or stage* or "Apn?ea Syndrome*" or "Apn?ea Obstructive" or "wake disorder*" or "disordered breathing" or DYSFUNTION or Problem*))) OR AB ((Sleep N1 (duration or disorder* or "Initiation and Maintenance Disorder" or stage* or "Apn?ea Syndrome*" or "Apn?ea

	Obstructive" or "wake disorder*" or "disordered breathing" or DYSFUNTION or Problem*)))
S5	TI "Social jetlag" OR AB "Social jetlag"
S4	TI "Biological Clock*" OR AB "Biological Clock*"
S3	TI (Morning* N2 Evening*) OR AB (Morning* N2 Evening*)
S2	TI ((Circadian N1 (rhythm or misalignment or clock*))) OR AB ((Circadian N1 (rhythm or misalignment or clock*)))
S1	TI Chronotype OR AB Chronotype

4) Cochrane Library

ID Search

#1 Chronotype

#2 (Circadian adj1 (rhythm or misalignment or clock*))

#3 Morning* adj2 Evening*

#4 Biological NEXT clock*

#5 "Social jetlag"

#6 (Sleep adj1 (duration or disorder* or "Initiation and Maintenance Disorder" or stage* or (Apn?ea NEXT Syndrome*) or (Apn?ea NEXT Obstructive) or (wake NEXT disorder*) or "disordered breathing" or DYSFUNTION or Problem*))

#7 Nap or naps or napping

#8 wakefulness

#9 Shift NEXT work*

#10 insomnia

#11 hyposomni*

#12 sleepless

#13 (early adj1 (awake* or wake or wakes or waking))

#14 ((difficult* or disturb* or inabilit* or unable* or problem* or reduced) adj3 (asleep or sleep*))

#15 MeSH descriptor: [Sleep Deprivation] explode all trees

#16 MeSH descriptor: [Circadian Rhythm] explode all trees

#17 MeSH descriptor: [Sleep Stages] explode all trees

#18 #1 OR #2 OR #3 OR #4 OR #5 OR #6 OR #7 OR #8 OR #9 OR #10 OR #11 OR #12 OR #13 OR #14 OR #15 OR #16 OR #17

#19 cognitive NEXT behavio?ral NEXT therap*

#20 cognitive NEXT therap*

#21 (Sleep adj1 (hygiene or education or program* or advice or extension))

#22 "bright light"

#23 "stimulus control"

#24 relaxation

#25 meditation

#26 mindfulness

#27 non-pharmacological

#28 MeSH descriptor: [Cognitive Behavioral Therapy] explode all trees

#29 MeSH descriptor: [Sleep Hygiene] this term only

#30 MeSH descriptor: [Meditation] this term only

#31 MeSH descriptor: [Relaxation Therapy] this term only

#32 MeSH descriptor: [Mindfulness] this term only

#33 #19 OR #20 OR #21 OR #22 OR #23 OR #24 OR #25 OR #26 OR #27 OR #28 OR #29 OR #30 OR #31 OR #32

#34 Weight

#35 "body mass index"

#36 ("body fat" adj1 percentage*)

#37 "waist to hip ratio"

#38 "waist to height ratio"

#39 (anthropometric adj2 (measure* OR indicator*))

#40 "Body Shape Index"

#41 "fat mass index"

#42 (Circumference adj1 (Waist or arm or neck))

#43 MeSH descriptor: [Body Weights and Measures] explode all trees

#44 "Average Density"

#45 "Body Volume Index"

#46 "Body Roundness Index"

#47 ((Obesity or obese or adiposity) adj1 Marker*)

#48 ((energy or Macronutrient or Carbohydrate or PROTEIN or fat) adj1 (expenditure or intake or density or balance))

#49 Portion NEXT size*

#50 "food reformulation"

#51 (calorie* or food intake or appetite or eating)

#52 "Basal Metabolic Rate" or BMR

#53 MeSH descriptor: [Basal Metabolism] explode all trees

#54 "Resting Metabolic Rate"

#55 "Diet Induced Thermogenesis"

#56 physical NEXT activit*

#57 "non-exercise activity thermogenesis"

#58 (sedentary adj1 (TIME or behavio?r or lifestyle))

#59 MeSH descriptor: [Sedentary Behavior] this term only

#60 #34 OR #35 OR #36 OR #37 OR #38 OR #39 OR #40 OR #41 OR #42 OR #43 OR #44 OR #45 OR #46 OR #47 OR #48 OR #49 OR #50 OR #51 OR #52 OR #53 OR #54 OR #55 OR #56 OR #57 OR #58 OR #59

#61 #18 AND #33 and #60

Appendix B. Eligibility criteria for study entry

	Inclusion criteria	Exclusion criteria
Population	Adults ≥ 18 years old<br>AND<br>Poor sleep health:<br>- Insomnia<br>- Short or long sleep duration<br>- Sleep abnormality (via validated questionnaire e.g. PSQI >5)<br>- Evening chronotype (via validated questionnaire)	Prevalent Obstructive Sleep Apnoea<br>Pregnancy<br>Animal studies
Intervention (Exposure)	Behavioural sleep intervention:<br>-Sleep extension<br>-Cognitive Behavioural Therapy for Insomnia<br>-Sleep hygiene/ education/ counselling	1) No Sleep intervention<br><br>2) Interventions of:<br>-CPAP/ mandibular devices<br>-bright/ dim light exposure<br>-pharmaceutical therapies<br>- laboratory-based sleep restriction/ deprivation<br><br>3) Interventions of:<br>- weight loss pharmacotherapies<br>- bariatric surgery<br>- chrononutrition.
Control	Routine/ standard care (i.e. No intervention)<br>Lifestyle program (without a sleep component) if available to both arms	
Outcomes of interest	Measured at both baseline + follow-up:<br>- Anthropometric markers<br>- Dietary intake<br>- Physical activity / sedentary behaviour measure	Not reporting the same outcome marker at both baseline and follow-up
Study designs	Any of:<br>Randomised controlled trials<br>Non-randomised trials<br>Quasi experimental<br>Single-arm cross-over ('pre-post')	Cross-sectional studies<br>Non-intervention studies

Key: CPAP = continuous positive airway pressure, OSA = obstructive sleep apnoea.

Appendix C. Risk of bias assessment for using traffic light plots and weighted summary plots (a) randomised controlled trials and (b) non-randomised studies

(a) (i) Traffic light plot for randomised controlled trials

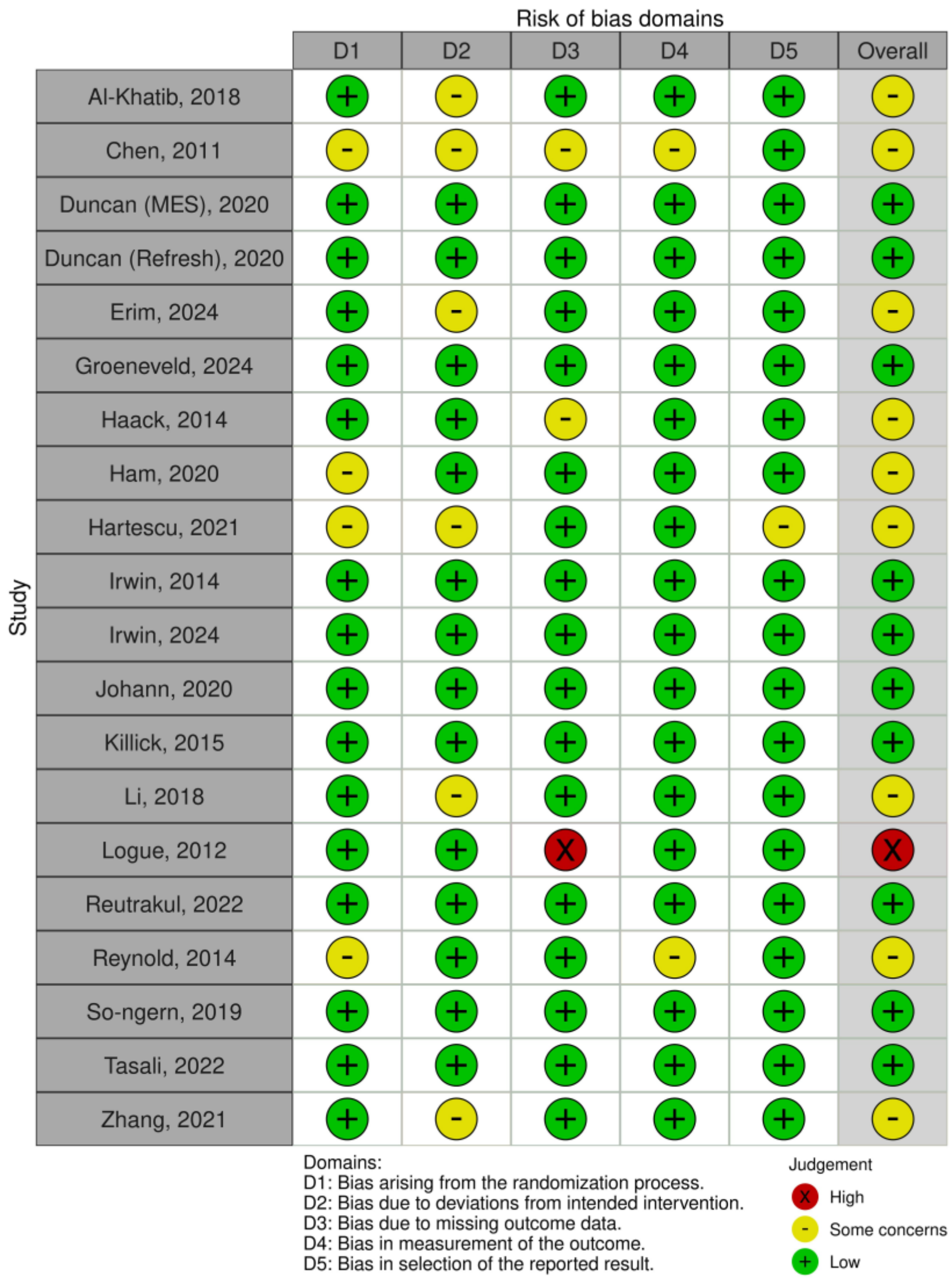

(a) (ii) Weighted summary plot for randomised controlled trials

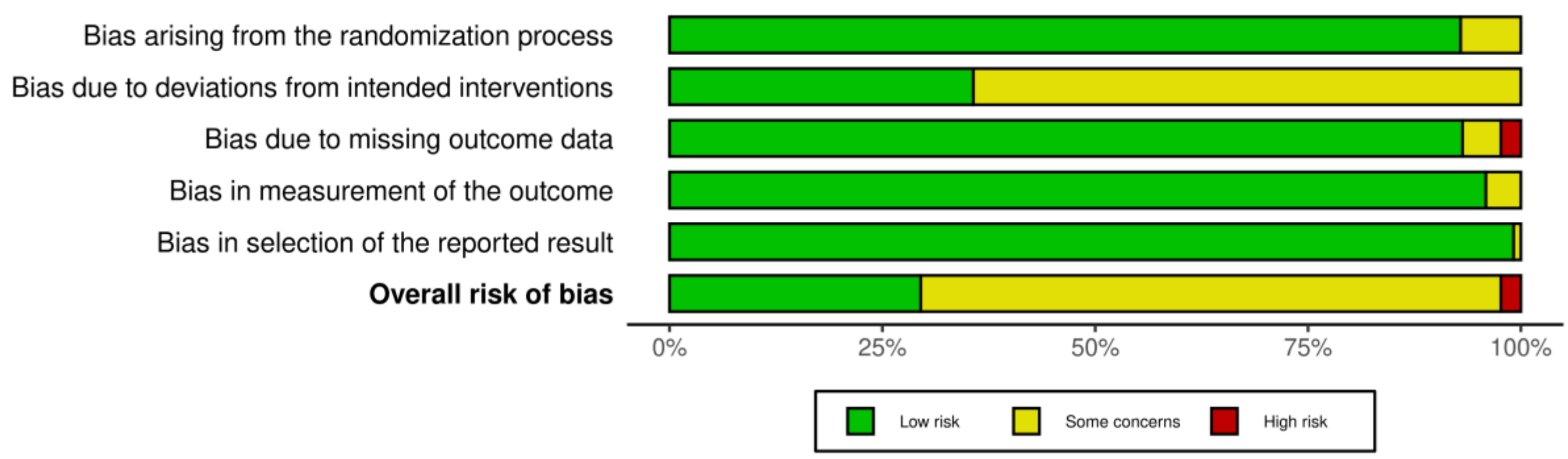

(b) (i) Traffic light plot for non-randomised studies

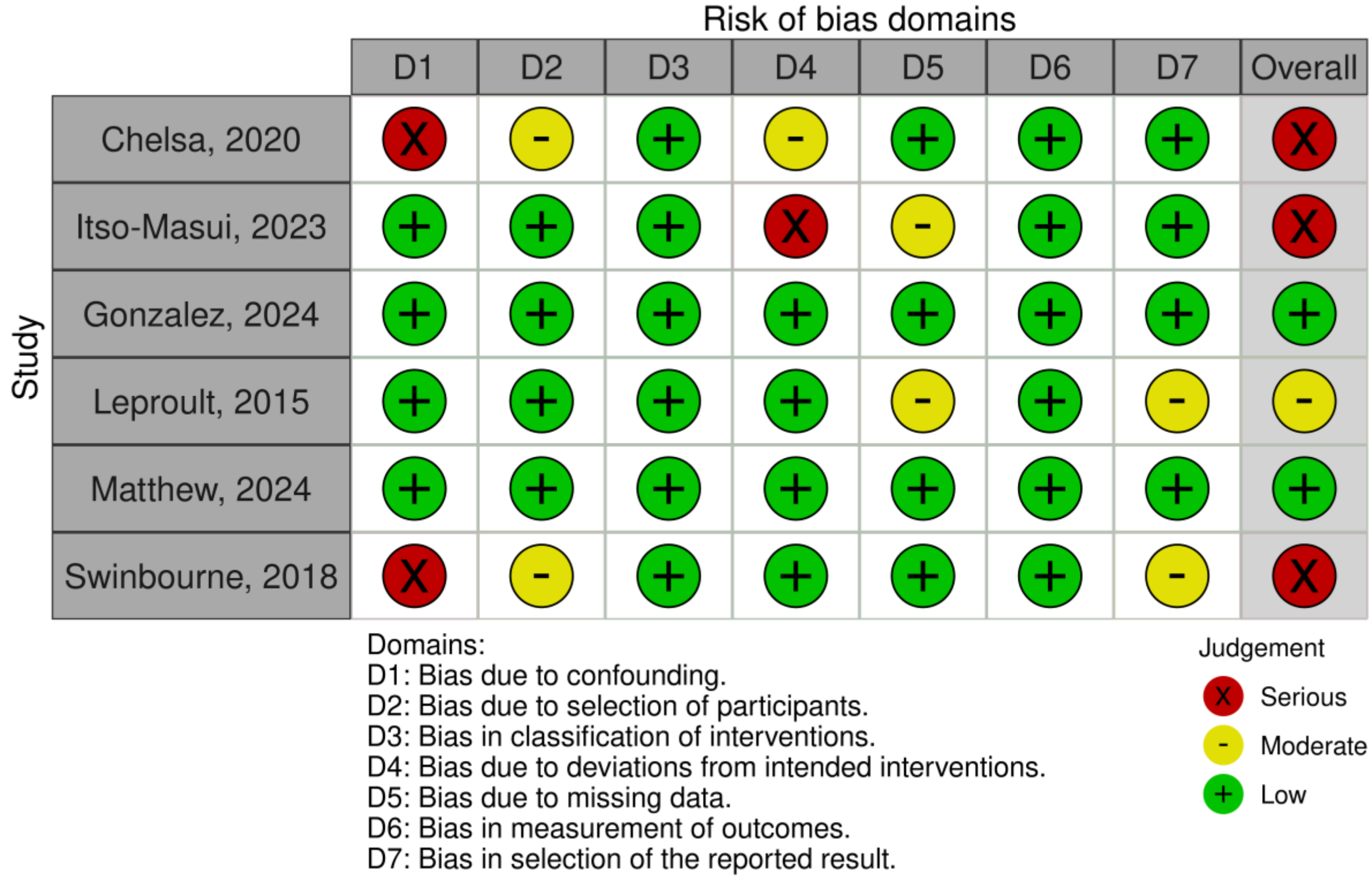

(b) (ii) Weighted summary plot for non-randomised trials

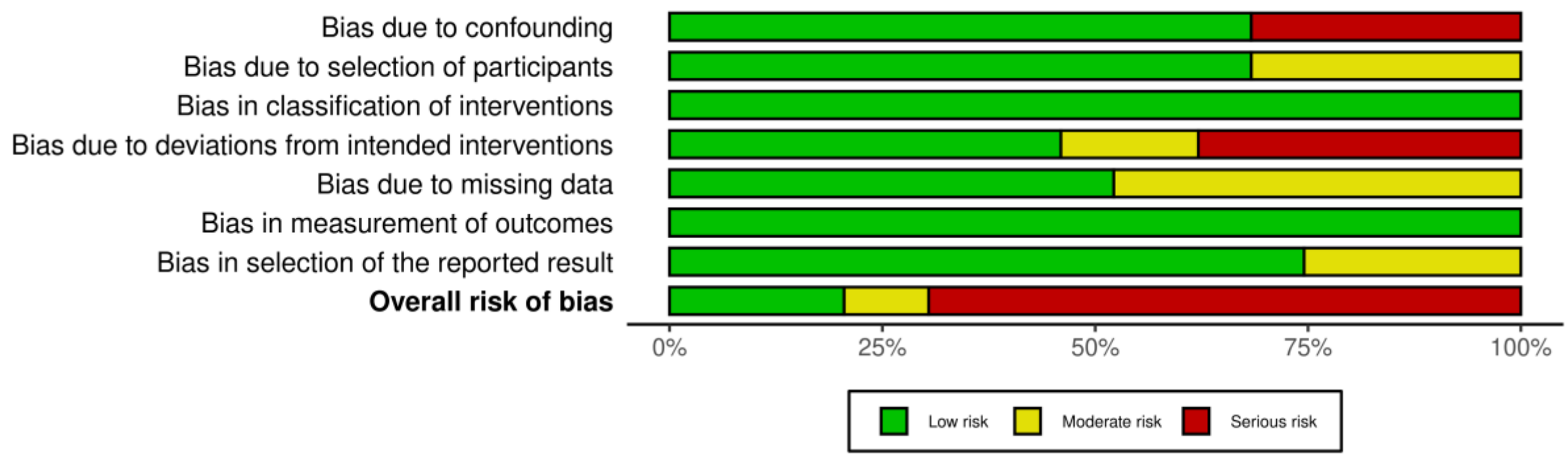

For summary plots, the weight was determined according to the number of people in each study. The risk of bias assessments were performed using Cochrane risk-of-bias tool for randomised trials version 2 (ROB-2) for randomised controlled trials and Risk-of-bias in non-randomised studies of interventions' (ROBINS-I) tool for non-randomised or single-arm ('pre-post') studies.

Appendix D: Forrest plots for the results of the meta-analysis examining the effect of sleep interventions on sleep variables: (a) Insomnia severity index and Pittsburgh Sleep Quality Index global scores and (b) sleep duration and time-in-bed.

(a) Pittsburgh Sleep Quality Index (PSQI) global score and Insomnia Severity Index (ISI) score

Study or Subgroup	Intervention Mean	Intervention SD	Intervention Total	Control Mean	Control SD	Control Total	Weight	Mean Difference IV, Random, 95% CI
**PSQI**								
AlKhatib 2018	−1.1	1.64	21	0.3	1.75	21	8.4%	−1.40 [−2.43, −0.37]
Carroll 2015	5.6	3	47	8.2	3.4	23	5.3%	−2.60 [−4.23, −0.97]
Chen 2011	9.9	3.7	37	11.6	3.6	35	5.1%	−1.70 [−3.39, −0.01]
Duncan (MES) 2020	5.31	2.25	14	5.34	3.27	23	4.8%	−0.03 [−1.81, 1.75]
Duncan (Refresh) 2020	7.23	3.61	74	9.91	3.99	44	6.1%	−2.68 [−4.12, −1.24]
Erim 2024	2.74	1.73	19	7.26	3.63	19	4.7%	−4.52 [−6.33, −2.71]
Ham 2020	8.3	3.4	24	11.11	2.32	20	5.1%	−2.81 [−4.51, −1.11]
Hartescu 2021	−5.55	2.92	10	−2.56	2.62	8	2.8%	−2.99 [−5.55, −0.43]
Ito-Masui 2023	7.84	2.46	61	9.1	4.1	61	7.4%	−1.26 [−2.46, −0.06]
Li 2018	−1.7	0.9	16	−0.4	0.7	15	11.3%	−1.30 [−1.87, −0.73]
Reutrakul 2022	−2.8	3.2	9	−2	4.2	5	1.2%	−0.80 [−5.03, 3.43]
Reynold 2014	4.75	1.75	8	3.67	1.75	6	4.5%	1.08 [−0.77, 2.93]
So-ngern 2019	4.33	0.42	21	4.81	0.42	21	12.9%	−0.48 [−0.73, −0.23]
Swinbourne 2018	3.5	1	25	5.4	2.7	25	7.8%	−1.90 [−3.03, −0.77]
Zhang 2021	9.01	2.67	506	9.91	2.34	527	12.7%	−0.90 [−1.21, −0.59]
**Subtotal (95% CI)**			**892**			**853**	**100.0%**	**−1.47 [−1.95, −0.99]**
Heterogeneity: $Tau^2 = 0.46$; $Chi^2 = 55.67$, df = 14 (P < 0.00001); $I^2 = 75\%$								
Test for overall effect: Z = 5.94 (P < 0.00001)								
**ISI**								
Duncan (MES) 2020	5.19	3.69	14	5.86	3.78	23	25.8%	−0.67 [−3.14, 1.80]
Groeneveld 2024	12.8	5.3	29	13.2	4.8	28	25.3%	−0.40 [−3.02, 2.22]
Ham 2020	9.78	4.09	24	14.39	6.26	20	23.2%	−4.61 [−7.80, −1.42]
Johann 2020	6.5	3.8	23	13	4.7	23	25.8%	−6.50 [−8.97, −4.03]
**Subtotal (95% CI)**			**90**			**94**	**100.0%**	**−3.02 [−6.09, 0.05]**
Heterogeneity: $Tau^2 = 7.92$; $Chi^2 = 15.86$, df = 3 (P = 0.001); $I^2 = 81\%$								
Test for overall effect: Z = 1.93 (P = 0.05)								

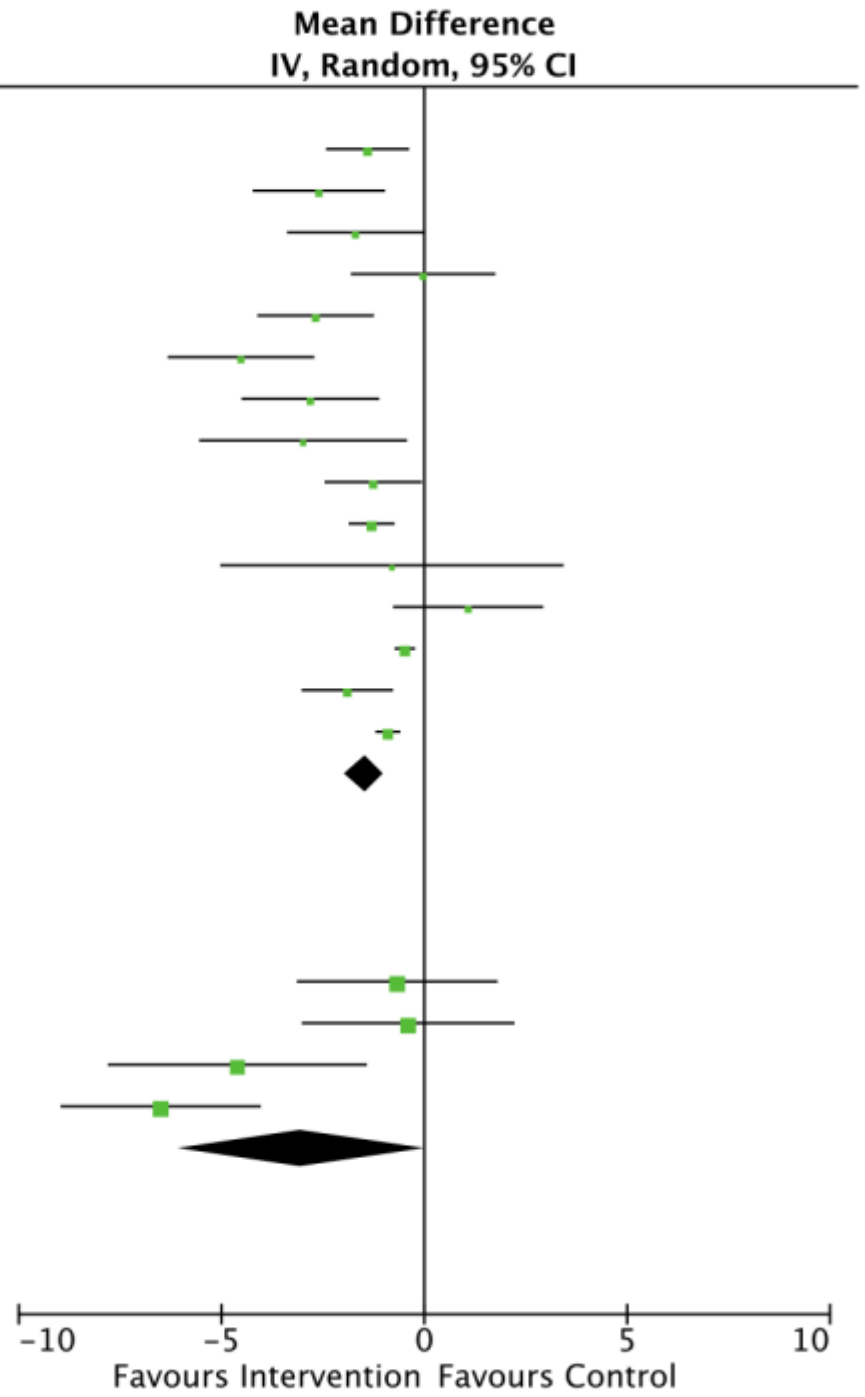

(b) Sleep duration and Time-in-bed (both measured in minutes)

Study or Subgroup	Intervention Mean	Intervention SD	Intervention Total	Control Mean	Control SD	Control Total	Weight	Mean Difference IV, Random, 95% CI
**Sleep duration**								
AlKhatib 2018	21	35.07	21	−11	35.07	21	11.6%	32.00 [10.79, 53.21]
Chen 2011	5.8	1.5	37	5.7	1.6	35	12.6%	0.10 [−0.62, 0.82]
Gonzalez 2024	549	36	22	405	45	22	11.3%	144.00 [119.92, 168.08]
Groeneveld 2024	414	102	29	444	72	28	9.0%	−30.00 [−75.71, 15.71]
Haack 2013	35	9	13	4	9	9	12.4%	31.00 [23.35, 38.65]
Hartescu 2021	78.48	15.45	10	6.28	15.46	8	12.1%	72.20 [57.83, 86.57]
Ito-Masui 2023	363.6	78	61	332.4	81.6	61	10.9%	31.20 [2.87, 59.53]
Matthew 2023	456	51	12	415.8	51	12	9.6%	40.20 [−0.61, 81.01]
Reutrakul 2022	26.9	42.5	9	−9.1	20.4	5	10.4%	36.00 [2.97, 69.03]
**Subtotal (95% CI)**			**214**			**201**	**100.0%**	**40.94 [15.01, 66.88]**
Heterogeneity: $Tau^2 = 1393.59$; $Chi^2 = 316.64$, df = 8 (P < 0.00001); $I^2 = 97\%$								
Test for overall effect: Z = 3.09 (P = 0.002)								
**Time-in-bed**								
AlKhatib 2018	21	35.07	21	−11	35.07	21	14.7%	32.00 [10.79, 53.21]
Chelsa 2020	438	36	12	468	30	14	14.6%	−30.00 [−55.73, −4.27]
Gonzalez 2024	633	41	22	468	52	22	14.5%	165.00 [137.33, 192.67]
Groeneveld 2024	498	72	29	522	60	28	14.2%	−24.00 [−58.36, 10.36]
Hartescu 2021	100.01	40.59	10	3.61	41.02	8	14.0%	96.40 [58.44, 134.36]
Reynold 2014	603	58	8	456	34	5	13.3%	147.00 [96.97, 197.03]
Swinbourne 2018	548.7	44.8	25	488	41	25	14.6%	60.70 [36.89, 84.51]
**Subtotal (95% CI)**			**127**			**123**	**100.0%**	**62.86 [8.98, 116.75]**
Heterogeneity: $Tau^2 = 5014.18$; $Chi^2 = 144.92$, df = 6 (P < 0.00001); $I^2 = 96\%$								
Test for overall effect: Z = 2.29 (P = 0.02)								

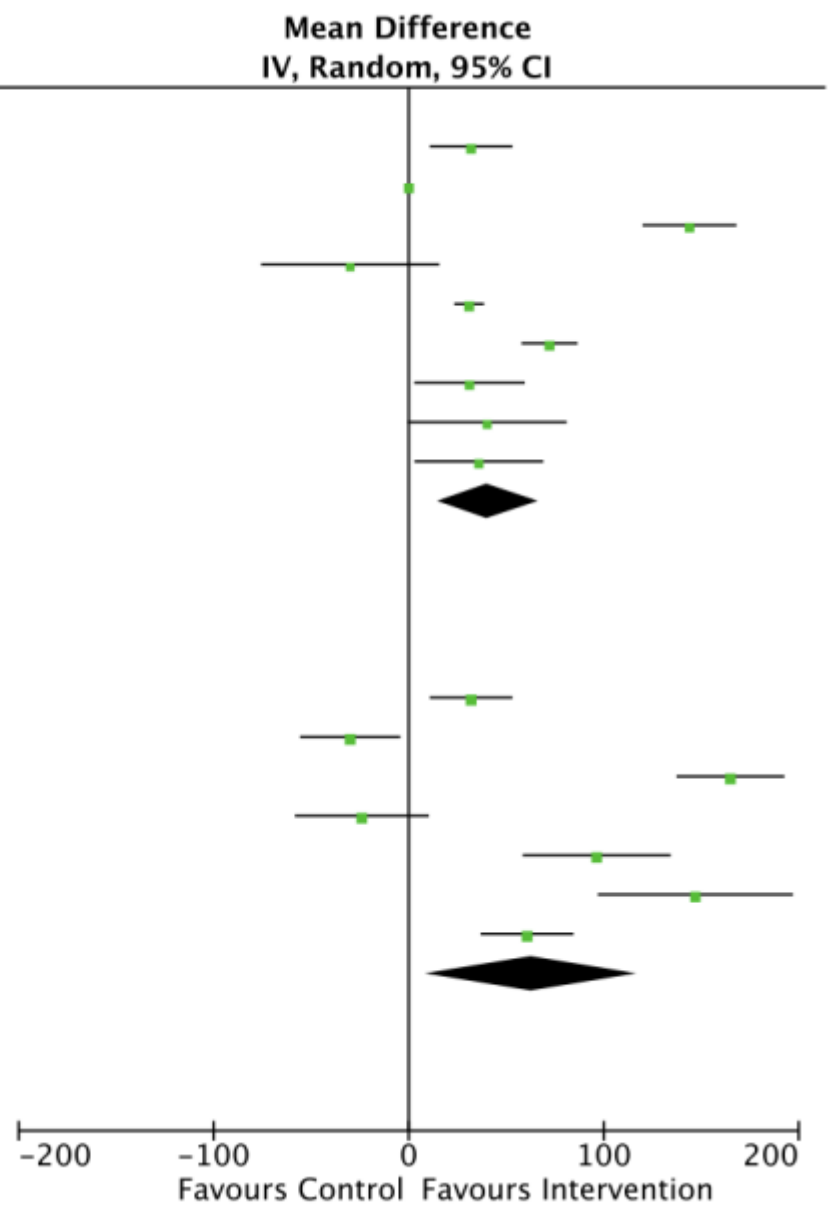

Key: CI= confidence interval, CBT-I = Cognitive Behavioural Therapy for Insomnia, I^2= heterogeneity test, IV = inverse variance, SD = standard deviation.

Appendix E. Forrest plots for the results of the meta-analysis assessing the effect of sleep interventions on (a) body weight (b) waist circumference, (c) percentage body fat and (d) fat free mass.

(a) i) Body weight (kg) for all studies (RCTs and non-RCTs combined)

Study or Subgroup	Intervention Mean	Intervention SD	Intervention Total	Control Mean	Control SD	Control Total	Weight	Mean Difference IV, Random, 95% CI
**All interventions**								
AlKhatib 2018	0.3	1.05	21	0.004	1.17	21	23.7%	0.30 [−0.38, 0.97]
Chelsa 2020	−4.4	13.83	11	−4.5	13.88	9	2.0%	0.10 [−12.11, 12.31]
Duncan (MES) 2020	87.92	14.95	14	82.63	13.86	23	3.0%	5.29 [−4.37, 14.95]
Duncan (Refresh) 2020	79.92	19.54	74	81.01	14.11	44	6.4%	−1.09 [−7.19, 5.01]
Erim 2024	−9.38	2.8	19	−3.69	1.53	19	21.2%	−5.69 [−7.12, −4.26]
Groeneveld 2024	1.24	26.75	25	−1.49	14.89	27	2.1%	2.73 [−9.17, 14.63]
Hartescu 2021	94	6.7	10	92.5	10	8	4.1%	1.50 [−6.58, 9.58]
Leproult 2015	60	9.6	16	60	9.7	16	5.6%	0.00 [−6.69, 6.69]
Reutrakul 2022	85.44	15.49	9	84.02	12.4	5	1.4%	1.42 [−13.43, 16.27]
So−ngern 2019	56.6	10.2	21	56.2	9.63	21	6.5%	0.40 [−5.60, 6.40]
Tasali 2022	−0.48	1.19	40	0.39	1.19	40	24.0%	−0.87 [−1.39, −0.35]
**Subtotal (95% CI)**			**260**			**233**	**100.0%**	**−1.09 [−2.88, 0.70]**
Heterogeneity: $Tau^2 = 3.40$; $Chi^2 = 57.40$, df = 10 (P < 0.00001); $I^2 = 83\%$								
Test for overall effect: Z = 1.19 (P = 0.23)								
**CBT−I +/− Sleep hygiene**								
Chelsa 2020	−4.4	13.83	11	−4.5	13.88	9	10.7%	0.10 [−12.11, 12.31]
Duncan (MES) 2020	87.92	14.95	14	82.63	13.86	23	14.8%	5.29 [−4.37, 14.95]
Duncan (Refresh) 2020	79.92	19.54	74	81.01	14.11	44	24.0%	−1.09 [−7.19, 5.01]
Erim 2024	−9.38	2.8	19	−3.69	1.53	19	39.3%	−5.69 [−7.12, −4.26]
Groeneveld 2024	1.24	26.75	25	−1.49	14.89	27	11.1%	2.73 [−9.17, 14.63]
**Subtotal (95% CI)**			**143**			**122**	**100.0%**	**−1.40 [−6.06, 3.26]**
Heterogeneity: $Tau^2 = 13.84$; $Chi^2 = 9.11$, df = 4 (P = 0.06); $I^2 = 56\%$								
Test for overall effect: Z = 0.59 (P = 0.56)								
**Sleep Extension**								
AlKhatib 2018	0.3	1.05	21	0.004	1.17	21	44.5%	0.30 [−0.38, 0.97]
Hartescu 2021	94	6.7	10	92.5	10	8	0.9%	1.50 [−6.58, 9.58]
Leproult 2015	60	9.6	16	60	9.7	16	1.3%	0.00 [−6.69, 6.69]
Reutrakul 2022	85.44	15.49	9	84.02	12.4	5	0.3%	1.42 [−13.43, 16.27]
So−ngern 2019	56.6	10.2	21	56.2	9.63	21	1.6%	0.40 [−5.60, 6.40]
Tasali 2022	−0.48	1.19	40	0.39	1.19	40	51.4%	−0.87 [−1.39, −0.35]
**Subtotal (95% CI)**			**117**			**111**	**100.0%**	**−0.29 [−1.07, 0.48]**
Heterogeneity: $Tau^2 = 0.23$; $Chi^2 = 7.58$, df = 5 (P = 0.18); $I^2 = 34\%$								
Test for overall effect: Z = 0.74 (P = 0.46)								

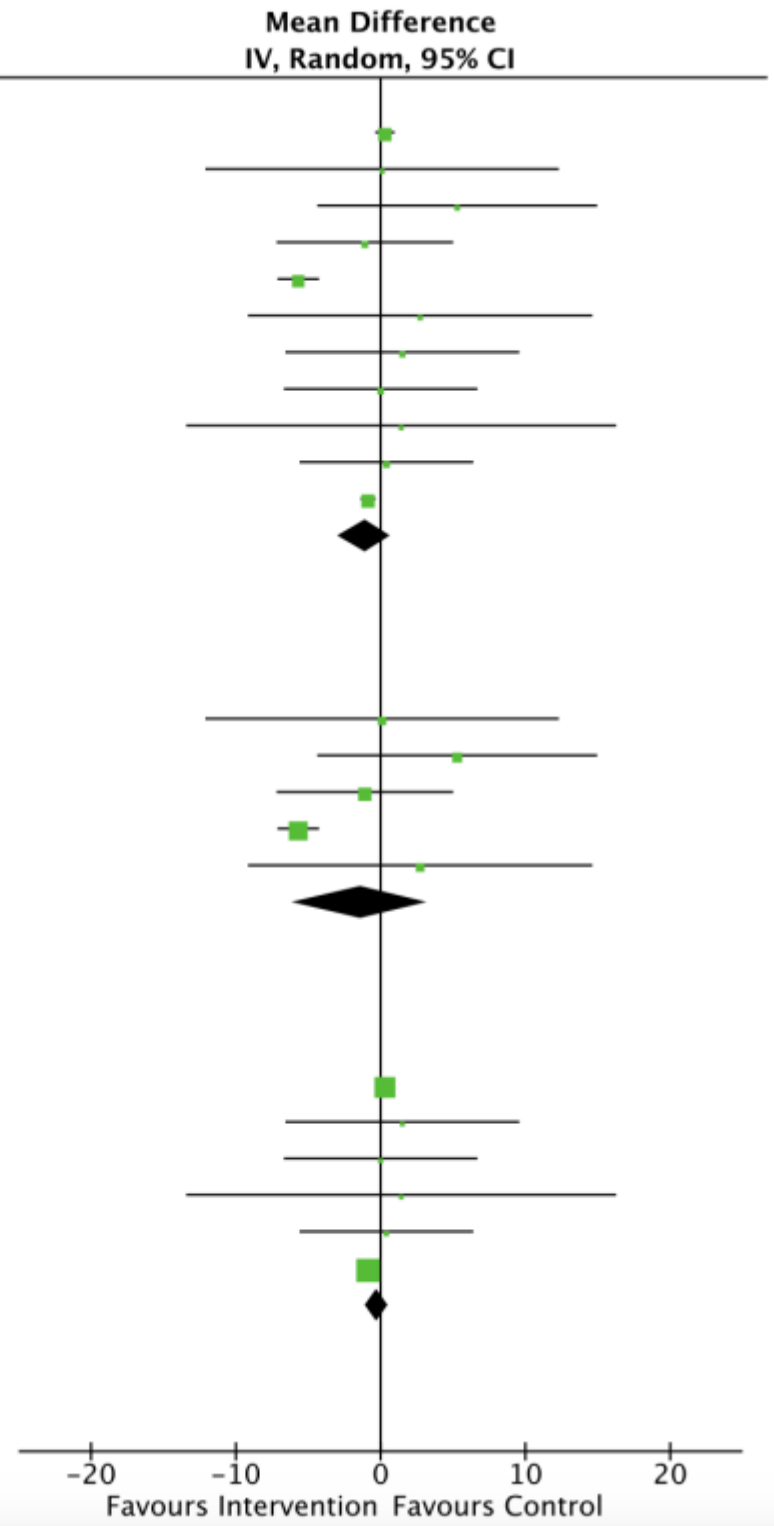

(a) ii) Body weight (kg) for RCTs only

Study or Subgroup	Intervention Mean	Intervention SD	Intervention Total	Control Mean	Control SD	Control Total	Weight	Mean Difference IV, Random, 95% CI
**All interventions**								
AlKhatib 2018	0.3	1.05	21	0.004	1.17	21	25.4%	0.30 [−0.38, 0.97]
Duncan (MES) 2020	87.92	14.95	14	82.63	13.86	23	3.4%	5.29 [−4.37, 14.95]
Duncan (Refresh) 2020	79.92	19.54	74	81.01	14.11	44	7.1%	−1.09 [−7.19, 5.01]
Erim 2024	−9.38	2.8	19	−3.69	1.53	19	22.8%	−5.69 [−7.12, −4.26]
Groeneveld 2024	1.24	26.75	25	−1.49	14.89	27	2.3%	2.73 [−9.17, 14.63]
Hartescu 2021	94	6.7	10	92.5	10	8	4.6%	1.50 [−6.58, 9.58]
Reutrakul 2022	85.44	15.49	9	84.02	12.4	5	1.5%	1.42 [−13.43, 16.27]
So−ngern 2019	56.6	10.2	21	56.2	9.63	21	7.3%	0.40 [−5.60, 6.40]
Tasali 2022	−0.48	1.19	40	0.39	1.19	40	25.7%	−0.87 [−1.39, −0.35]
**Subtotal (95% CI)**			**233**			**208**	**100.0%**	**−1.16 [−3.06, 0.74]**
Heterogeneity: $Tau^2 = 3.58$; $Chi^2 = 57.32$, df = 8 (P < 0.00001); $I^2 = 86\%$								
Test for overall effect: Z = 1.20 (P = 0.23)								
**CBT−I +/− Sleep hygiene**								
Duncan (MES) 2020	87.92	14.95	14	82.63	13.86	23	17.6%	5.29 [−4.37, 14.95]
Duncan (Refresh) 2020	79.92	19.54	74	81.01	14.11	44	27.3%	−1.09 [−7.19, 5.01]
Erim 2024	−9.38	2.8	19	−3.69	1.53	19	41.7%	−5.69 [−7.12, −4.26]
Groeneveld 2024	1.24	26.75	25	−1.49	14.89	27	13.5%	2.73 [−9.17, 14.63]
**Subtotal (95% CI)**			**132**			**113**	**100.0%**	**−1.37 [−6.64, 3.90]**
Heterogeneity: $Tau^2 = 16.80$; $Chi^2 = 8.41$, df = 3 (P = 0.04); $I^2 = 64\%$								
Test for overall effect: Z = 0.51 (P = 0.61)								
**Sleep Extension**								
AlKhatib 2018	0.3	1.05	21	0.004	1.17	21	45.5%	0.30 [−0.38, 0.97]
Hartescu 2021	94	6.7	10	92.5	10	8	1.2%	1.50 [−6.58, 9.58]
Reutrakul 2022	85.44	15.49	9	84.02	12.4	5	0.4%	1.42 [−13.43, 16.27]
So−ngern 2019	56.6	10.2	21	56.2	9.63	21	2.1%	0.40 [−5.60, 6.40]
Tasali 2022	−0.48	1.19	40	0.39	1.19	40	50.9%	−0.87 [−1.39, −0.35]
**Subtotal (95% CI)**			**101**			**95**	**100.0%**	**−0.28 [−1.16, 0.61]**
Heterogeneity: $Tau^2 = 0.33$; $Chi^2 = 7.56$, df = 4 (P = 0.11); $I^2 = 47\%$								
Test for overall effect: Z = 0.62 (P = 0.54)								

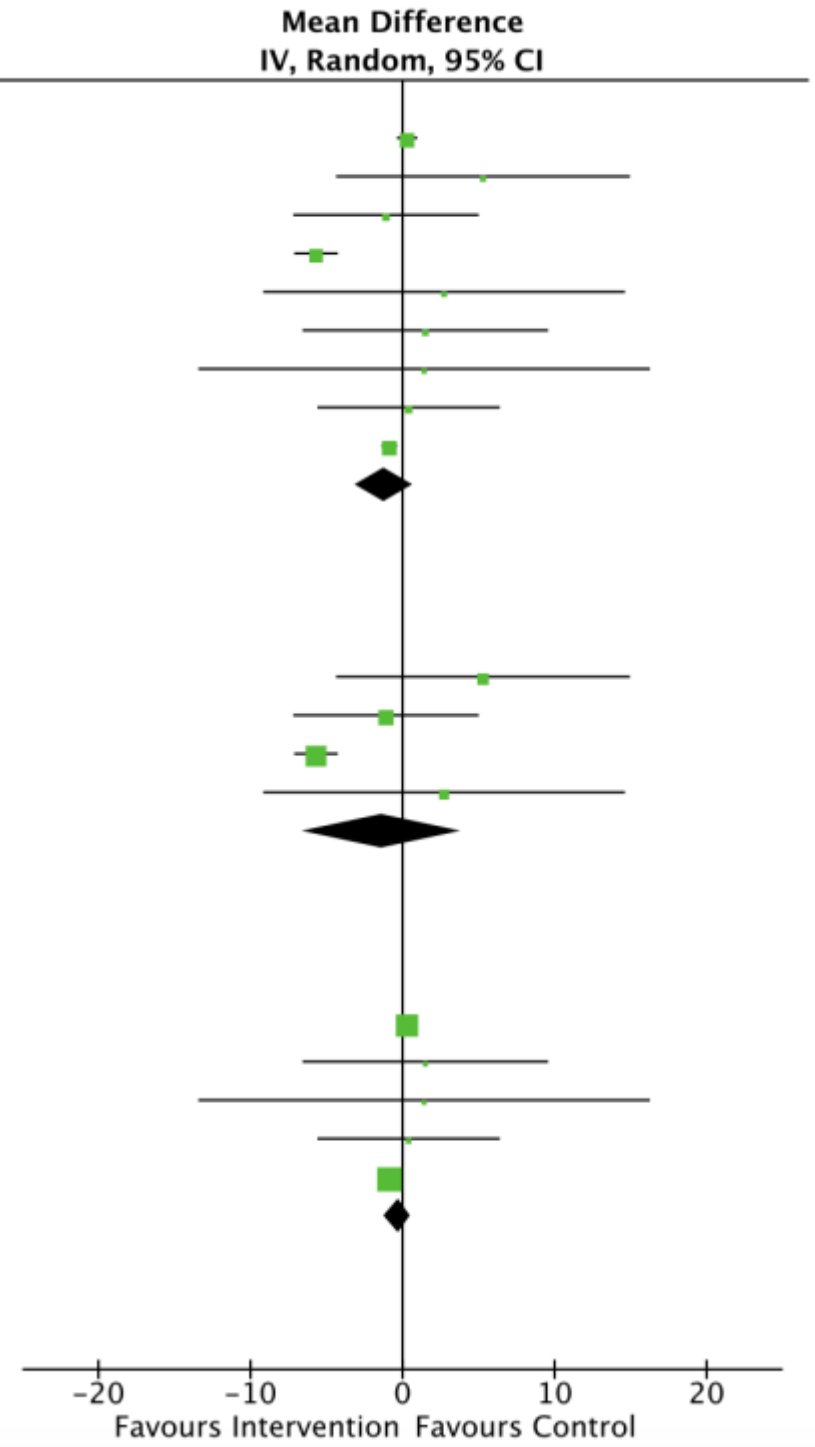

(b) Waist circumference (cm)

Study or Subgroup	Intervention Mean	Intervention SD	Intervention Total	Control Mean	Control SD	Control Total	Weight	Mean Difference IV, Random, 95% CI
**All interventions**								
AlKhatib 2018	−0.2	3.51	21	0.2	3.51	21	19.8%	−0.40 [−2.52, 1.72]
Chelsa 2020	97	10.4	12	99.8	12.4	14	8.7%	−2.80 [−11.56, 5.96]
Duncan 2020	96.17	5.6	14	95.4	7.06	23	16.2%	0.77 [−3.34, 4.88]
Erim 2024	−9.89	5.15	19	−2.58	1.26	19	19.4%	−7.31 [−9.69, −4.93]
Groeneveld 2024	105.1	19.2	29	101.4	11.1	28	9.5%	3.70 [−4.41, 11.81]
Ham 2020	84.07	8.53	24	83.62	8.13	20	14.7%	0.45 [−4.48, 5.38]
Hartescu 2021	105.34	6.17	10	104.1	7.79	8	11.7%	1.24 [−5.38, 7.86]
**Subtotal (95% CI)**			**129**			**133**	**100.0%**	**−1.05 [−4.41, 2.31]**
Heterogeneity: Tau² = 13.68; Chi² = 26.59, df = 6 (P = 0.0002); I² = 77%								
Test for overall effect: Z = 0.61 (P = 0.54)								
**CBT−I +/− Sleep hygiene**								
Chelsa 2020	97	10.4	12	99.8	12.4	14	14.3%	−2.80 [−11.56, 5.96]
Duncan 2020	96.17	5.6	14	95.4	7.06	23	23.0%	0.77 [−3.34, 4.88]
Erim 2024	−9.89	5.15	19	−2.58	1.26	19	25.9%	−7.31 [−9.69, −4.93]
Groeneveld 2024	105.1	19.2	29	101.4	11.1	28	15.4%	3.70 [−4.41, 11.81]
Ham 2020	84.07	8.53	24	83.62	8.13	20	21.4%	0.45 [−4.48, 5.38]
**Subtotal (95% CI)**			**98**			**104**	**100.0%**	**−1.45 [−6.23, 3.33]**
Heterogeneity: Tau² = 21.51; Chi² = 19.23, df = 4 (P = 0.0007); I² = 79%								
Test for overall effect: Z = 0.59 (P = 0.55)								
**Sleep Extension**								
AlKhatib 2018	−0.2	3.51	21	0.2	3.51	21	90.7%	−0.40 [−2.52, 1.72]
Hartescu 2021	105.34	6.17	10	104.1	7.79	8	9.3%	1.24 [−5.38, 7.86]
**Subtotal (95% CI)**			**31**			**29**	**100.0%**	**−0.25 [−2.27, 1.77]**
Heterogeneity: Tau² = 0.00; Chi² = 0.21, df = 1 (P = 0.64); I² = 0%								
Test for overall effect: Z = 0.24 (P = 0.81)								

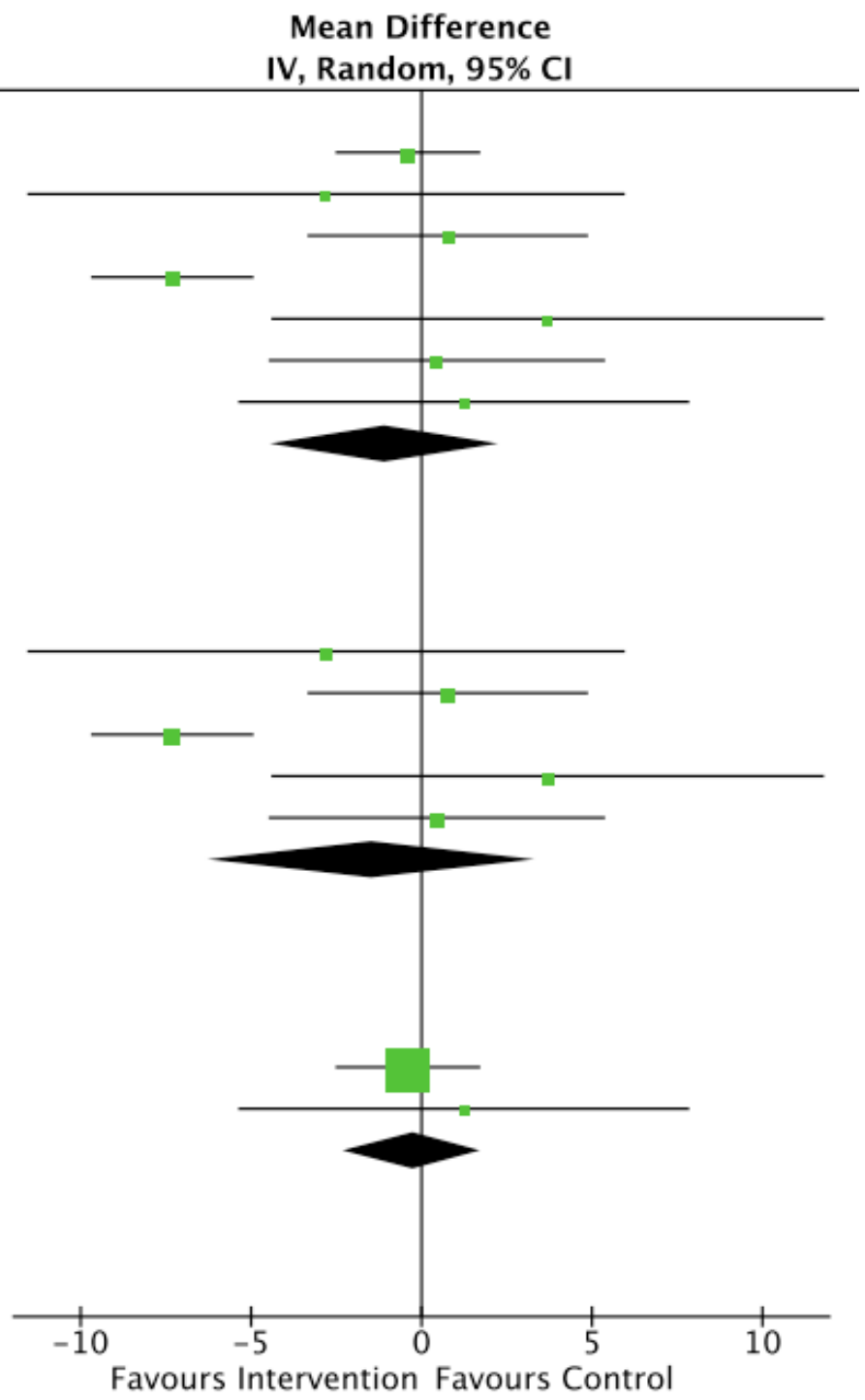

(d) Percentage body fat

Study or Subgroup	Intervention Mean	Intervention SD	Intervention Total	Control Mean	Control SD	Control Total	Weight	Mean Difference IV, Random, 95% CI
AlKhatib 2018	0.03	2.11	21	−0.1	2.22	21	50.0%	0.13 [−1.18, 1.44]
Haack 2013	24	1.5	13	30.2	1.6	9	50.0%	−6.20 [−7.53, −4.87]
**Total (95% CI)**			**34**			**30**	**100.0%**	**−3.03 [−9.24, 3.17]**
Heterogeneity: Tau² = 19.58; Chi² = 44.31, df = 1 (P < 0.00001); I² = 98%								
Test for overall effect: Z = 0.96 (P = 0.34)								

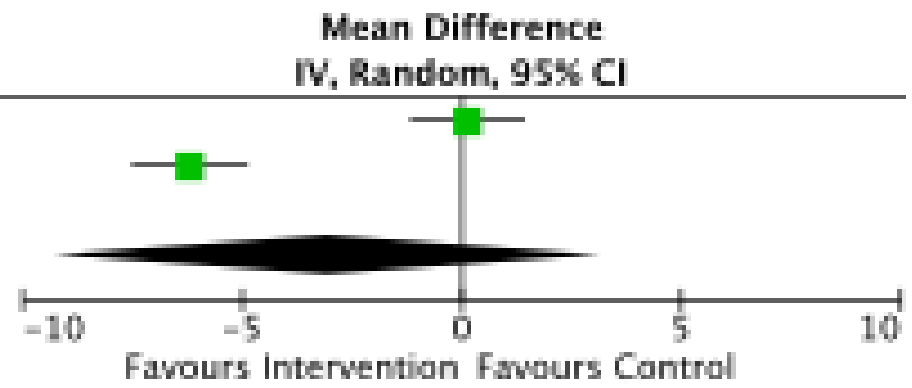

(e) Fat free mass (kg)

Study or Subgroup	Intervention Mean	Intervention SD	Intervention Total	Control Mean	Control SD	Control Total	Weight	Mean Difference IV, Random, 95% CI
AlKhatib 2018	0.2	1.64	21	0.05	1.75	21	30.3%	0.15 [−0.88, 1.18]
Tasali 2022	−0.36	0.9	40	0.26	0.89	40	69.7%	−0.62 [−1.01, −0.23]
**Total (95% CI)**			**61**			**61**	**100.0%**	**−0.39 [−1.08, 0.31]**
Heterogeneity: Tau² = 0.14; Chi² = 1.89, df = 1 (P = 0.17); I² = 47%								
Test for overall effect: Z = 1.09 (P = 0.27)								

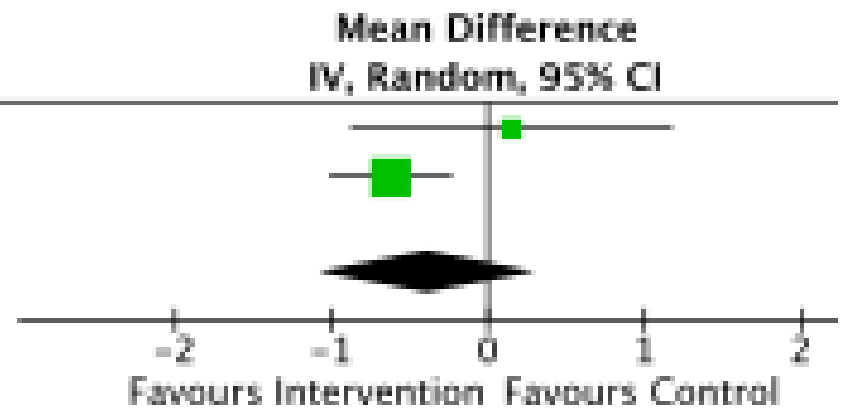

Key: CI= confidence interval, CBT-I = Cognitive Behavioural Therapy for Insomnia, cm = centimetre, I^2= heterogeneity test, IV = inverse variance, SD = standard deviation.

Appendix F. Forrest plots for the results of the meta-analysis assessing the effect of sleep interventions on dietary intake

a) Daily calorie intake outcome: types of nutritional assessment techniques used in individual studies assessing daily energy intake in the meta-analysis

**Study**	**Study type**	**Assessment technique**	**Measurement category**
Al-khatib et al., 2018	RCT	7-day food diary	Subjective
Duncan et al., (MES), 2020	RCT	Recall via an eating survey	Subjective
Haack et al., 2013	RCT	Food diary	Subjective
So-ngern et al., 2019	RCT	Food diary (3 days per week)	Subjective
Tasali et al., 2022	RCT	Doubly labelled water method	Objective

b) **Forrest plot for the results of the meta-analysis examining the effect of sleep interventions on daily calorie intake for subjective nutritional assessment methods only: (i) all studies and (ii) studies with a raised body mass index at baseline.**

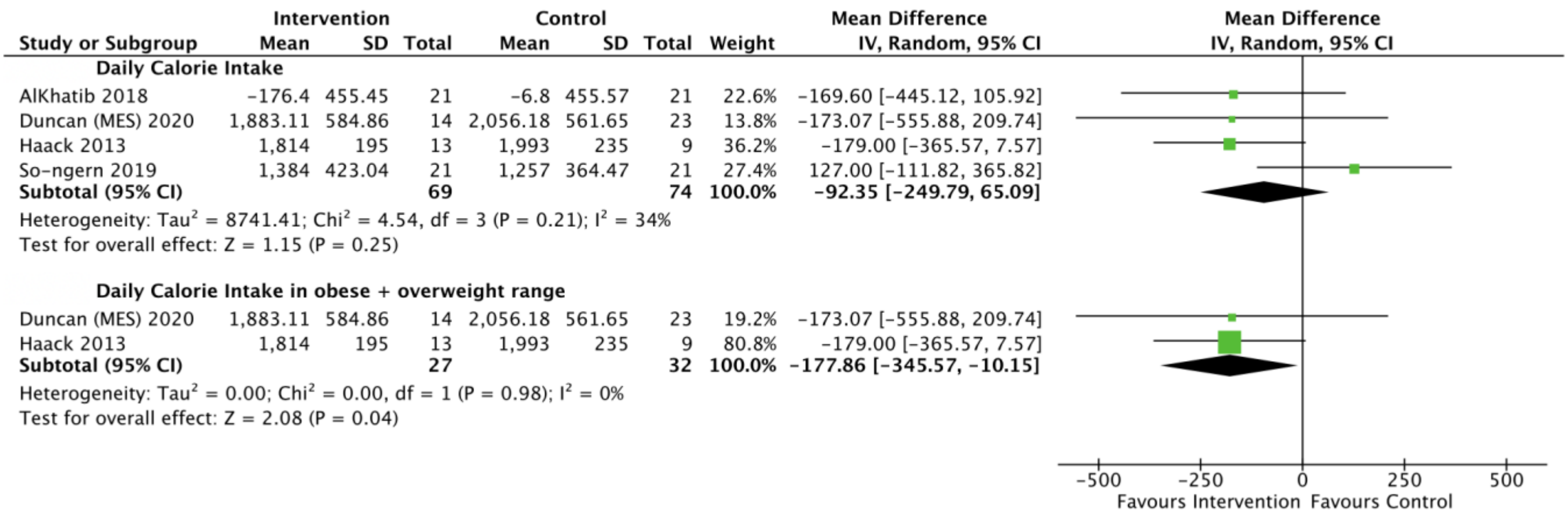

In the above sensitivity analysis, Tasali et al. was removed from the analysis, leaving just the subjective assessments methods. Even after this exclusion, the average reduction in daily calorie intake was significant in people with a raised BMI (either overweight or obese), (see Figure 3, main text). Key: CI= confidence interval, I^2= heterogeneity test, IV = inverse variance, SD = standard deviation.

(c) Forrest plots for the results of the meta-analysis assessing the effect of sleep interventions on daily quantities of food consumption

(1) Sugar intake (grams/ day)

Study or Subgroup	Intervention Mean	Intervention SD	Intervention Total	Control Mean	Control SD	Control Total	Weight	Mean Difference IV, Random, 95% CI
AlKhatib 2018	−14.3	24.9	21	−2.8	24.78	21	69.6%	−11.50 [−26.52, 3.52]
Duncan 2020	99.5	36.54	14	114.6	30.09	23	30.4%	−15.10 [−37.85, 7.65]
**Total (95% CI)**			**35**			**44**	**100.0%**	**−12.59 [−25.13, −0.06]**

Heterogeneity: Tau² = 0.00; Chi² = 0.07, df = 1 (P = 0.80); I² = 0%
Test for overall effect: Z = 1.97 (P = 0.05)

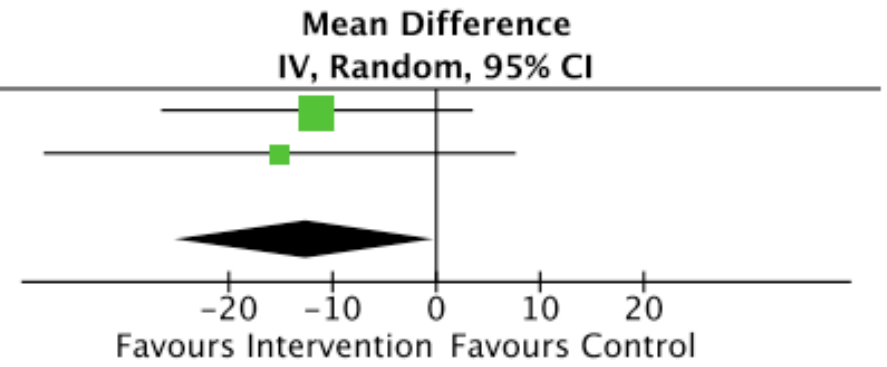

(2) Fibre (grams/ day)

Study or Subgroup	Intervention Mean	Intervention SD	Intervention Total	Control Mean	Control SD	Control Total	Weight	Mean Difference IV, Random, 95% CI
AlKhatib 2018	−3.6	7.01	21	−1.3	7.01	21	60.8%	−2.30 [−6.54, 1.94]
Duncan 2020	27.8	8.61	14	27.4	6.71	23	39.2%	0.40 [−4.88, 5.68]
**Total (95% CI)**			**35**			**44**	**100.0%**	**−1.24 [−4.55, 2.06]**

Heterogeneity: Tau² = 0.00; Chi² = 0.61, df = 1 (P = 0.43); I² = 0%
Test for overall effect: Z = 0.74 (P = 0.46)

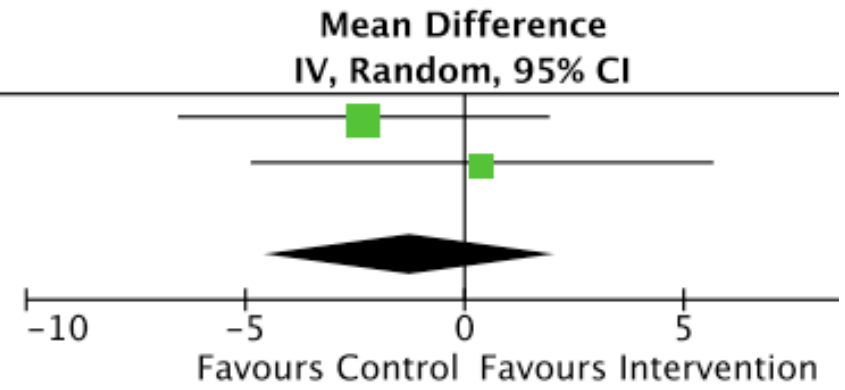

(3) Sodium (milligrams/ day)

Study or Subgroup	Intervention Mean	Intervention SD	Intervention Total	Control Mean	Control SD	Control Total	Weight	Mean Difference IV, Random, 95% CI
Duncan 2020	1,814.7	517.47	14	2,077.5	624.42	23	36.7%	−262.80 [−635.08, 109.48]
Haack 2013	2,610	302	13	2,621	332	9	63.3%	−11.00 [−283.02, 261.02]
**Total (95% CI)**			**27**			**32**	**100.0%**	**−103.51 [−341.43, 134.41]**

Heterogeneity: Tau² = 4030.72; Chi² = 1.15, df = 1 (P = 0.28); I² = 13%
Test for overall effect: Z = 0.85 (P = 0.39)

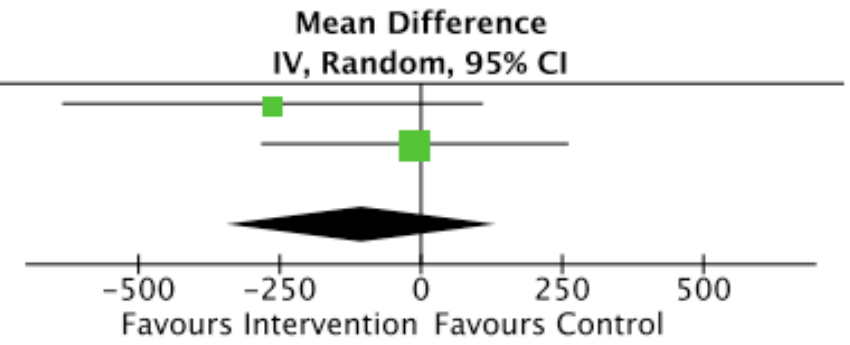

(4) Dietary composition (% of total daily energy intake)

Study or Subgroup	Intervention Mean	Intervention SD	Intervention Total	Control Mean	Control SD	Control Total	Weight	Mean Difference IV, Random, 95% CI
**Protein**								
AlKhatib 2018	1.6	4.33	21	−1.9	4.29	21	45.3%	3.50 [0.89, 6.11]
Duncan 2020	21.3	4.12	14	18.9	2.4	23	54.7%	2.40 [0.03, 4.77]
**Subtotal (95% CI)**			**35**			**44**	**100.0%**	**2.90 [1.14, 4.65]**
Heterogeneity: Tau² = 0.00; Chi² = 0.37, df = 1 (P = 0.54); I² = 0%								
Test for overall effect: Z = 3.24 (P = 0.001)								
**Carbohydrate**								
AlKhatib 2018	0.2	4.91	21	0.4	5.03	21	67.1%	−0.20 [−3.21, 2.81]
Duncan 2020	40.5	7.86	14	43.6	4.32	23	32.9%	−3.10 [−7.58, 1.38]
**Subtotal (95% CI)**			**35**			**44**	**100.0%**	**−1.15 [−3.83, 1.52]**
Heterogeneity: Tau² = 0.42; Chi² = 1.11, df = 1 (P = 0.29); I² = 10%								
Test for overall effect: Z = 0.85 (P = 0.40)								
**Saturated fat**								
AlKhatib 2018	−0.7	3.27	21	0.2	3.27	21	37.4%	−0.90 [−2.88, 1.08]
Duncan 2020	12.6	2.24	14	13.3	2.4	23	62.6%	−0.70 [−2.23, 0.83]
**Subtotal (95% CI)**			**35**			**44**	**100.0%**	**−0.77 [−1.98, 0.44]**
Heterogeneity: Tau² = 0.00; Chi² = 0.02, df = 1 (P = 0.88); I² = 0%								
Test for overall effect: Z = 1.26 (P = 0.21)								
**Total Fat**								
AlKhatib 2018	−2.1	5.03	21	1	5.03	21	51.7%	−3.10 [−6.14, −0.06]
Duncan 2020	33.3	5.99	14	31.6	3.84	23	48.3%	1.70 [−1.81, 5.21]
**Subtotal (95% CI)**			**35**			**44**	**100.0%**	**−0.78 [−5.48, 3.92]**
Heterogeneity: Tau² = 8.71; Chi² = 4.10, df = 1 (P = 0.04); I² = 76%								
Test for overall effect: Z = 0.33 (P = 0.74)								

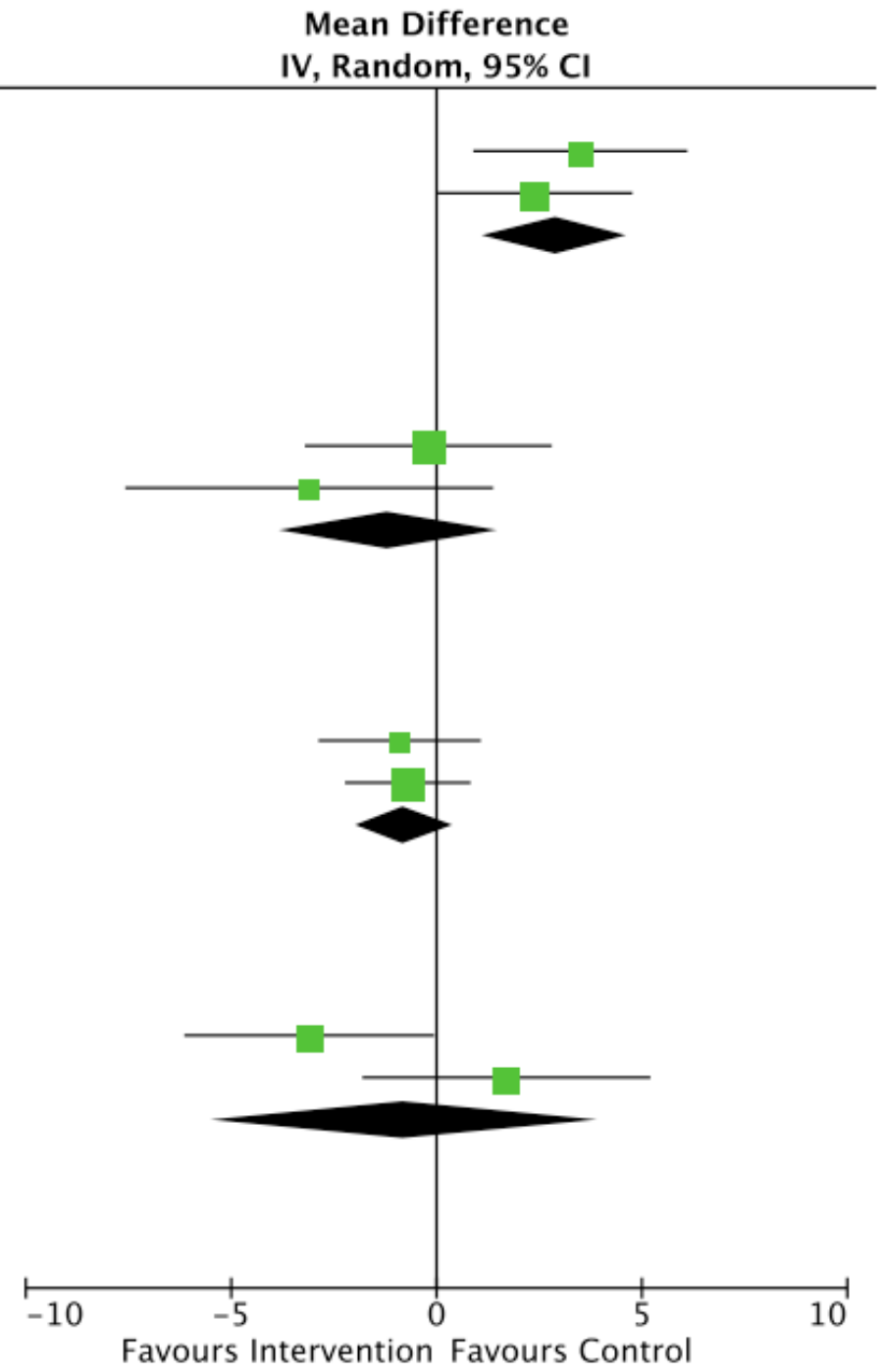

Key: CI= confidence interval, I^2 = heterogeneity test, IV = inverse variance, SD = standard deviation.

Appendix G. Forrest plots for the results of the meta-analysis assessing the effect of sleep interventions on physical activity and sedentary behaviour

(a) Total activity (Metabolic equivalents / week)

Study or Subgroup	Intervention Mean	Intervention SD	Intervention Total	Control Mean	Control SD	Control Total	Weight	Mean Difference IV, Random, 95% CI
Chelsa 2020	1,940	1,532	12	1,403	1,210	14	46.1%	537.00 [-536.81, 1610.81]
Hartescu 2021	-99.28	1,840.03	10	788.52	1,190.7	8	41.6%	-887.80 [-2295.42, 519.82]
Reutrakul 2022	1,847	3,200	9	-3,188	4,341	4	12.3%	5035.00 [294.95, 9775.05]
**Total (95% CI)**			**31**			**26**	**100.0%**	**497.74 [-1394.90, 2390.37]**

Heterogeneity: Tau² = 1724293.62; Chi² = 6.66, df = 2 (P = 0.04); I² = 70%
Test for overall effect: Z = 0.52 (P = 0.61)

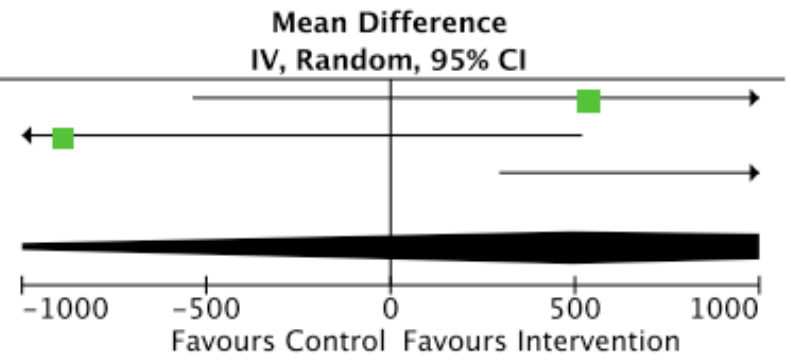

(b) Light and MVPA (minutes per day)

Study or Subgroup	Intervention Mean	Intervention SD	Intervention Total	Control Mean	Control SD	Control Total	Weight	Mean Difference IV, Random, 95% CI
**Light**								
Duncan 2020	208.14	62.63	14	222.45	184.93	23	50.2%	-14.31 [-96.70, 68.08]
Gonzalez 2024	753	135.56	22	860	145.93	22	49.8%	-107.00 [-190.23, -23.77]
**Subtotal (95% CI)**			**36**			**45**	**100.0%**	**-60.46 [-151.29, 30.37]**
Heterogeneity: Tau² = 2510.53; Chi² = 2.41, df = 1 (P = 0.12); I² = 58%								
Test for overall effect: Z = 1.30 (P = 0.19)								
**Moderate and Vigorous**								
Duncan 2020	70.43	35.78	14	76.66	45.35	23	13.5%	-6.23 [-32.59, 20.13]
Gonzalez 2024	24	31.85	22	22.33	25.93	22	31.9%	1.67 [-15.49, 18.83]
Matthew 2023	59.23	27.39	11	67.39	29.99	11	16.3%	-8.16 [-32.16, 15.84]
Refresh Duncan 2020	41.67	45.5	74	36.19	39.68	44	38.3%	5.48 [-10.17, 21.13]
**Subtotal (95% CI)**			**121**			**100**	**100.0%**	**0.46 [-9.23, 10.15]**
Heterogeneity: Tau² = 0.00; Chi² = 1.16, df = 3 (P = 0.76); I² = 0%								
Test for overall effect: Z = 0.09 (P = 0.93)								

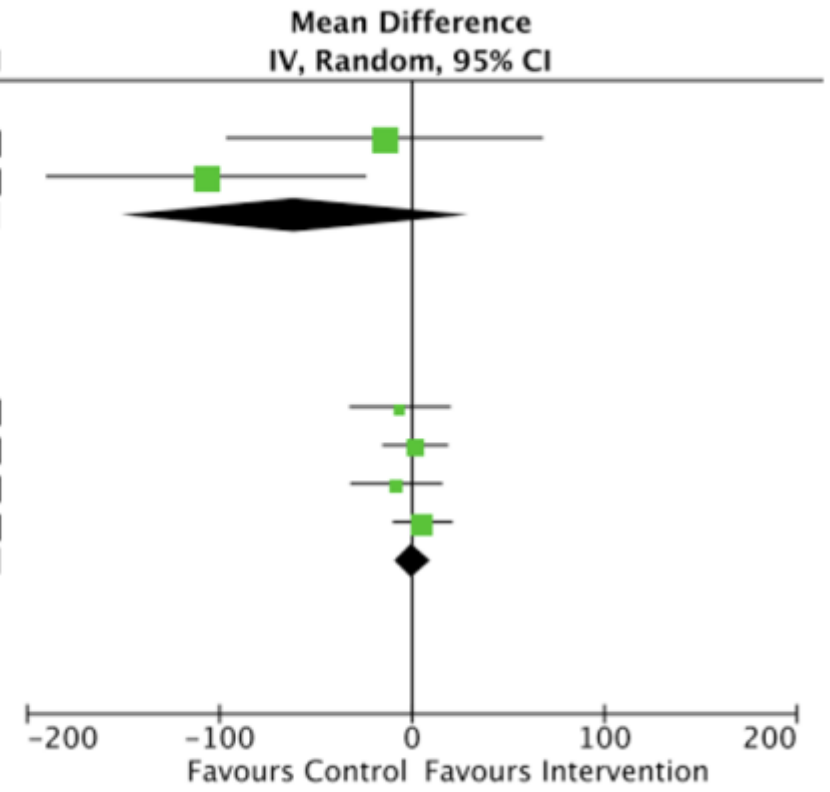

(c) Sedentary behaviour and sitting time (minutes per day)

Study or Subgroup	Intervention Mean	Intervention SD	Intervention Total	Control Mean	Control SD	Control Total	Weight	Mean Difference IV, Random, 95% CI
**Sitting time**								
Duncan 2020	618.45	167.79	14	549.21	150.55	23	32.1%	69.24 [-38.05, 176.53]
Refresh Duncan 2020	614.51	187.39	74	595.21	203.29	44	67.9%	19.30 [-54.39, 92.99]
**Subtotal (95% CI)**			**88**			**67**	**100.0%**	**35.31 [-25.44, 96.05]**
Heterogeneity: Tau² = 0.00; Chi² = 0.57, df = 1 (P = 0.45); I² = 0%								
Test for overall effect: Z = 1.14 (P = 0.25)								
**Sedentary behaviour**								
Duncan 2020	699.66	122.59	14	594	108.24	23	48.3%	105.66 [27.68, 183.64]
Matthew 2023	634.1	60.66	11	676.31	74.39	11	51.7%	-42.21 [-98.93, 14.51]
**Subtotal (95% CI)**			**25**			**34**	**100.0%**	**29.21 [-115.62, 174.03]**
Heterogeneity: Tau² = 9722.55; Chi² = 9.03, df = 1 (P = 0.003); I² = 89%								
Test for overall effect: Z = 0.40 (P = 0.69)								

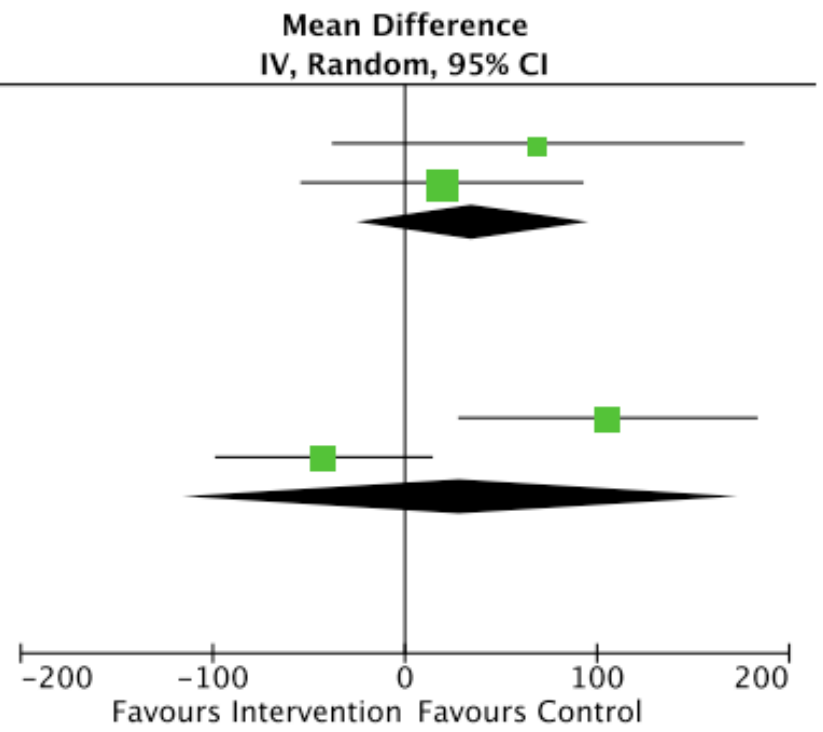

(d) Daily step count

Study or Subgroup	Intervention Mean	Intervention SD	Intervention Total	Control Mean	Control SD	Control Total	Weight	Mean Difference IV, Random, 95% CI
Chelsa 2020	7,277	2,280	12	8,134	3,480	14	12.9%	-857.00 [-3090.18, 1376.18]
Gonzalez 2024	6,304	3,925	22	6,198	3,249	22	14.2%	106.00 [-2023.13, 2235.13]
Ito-Masui 2023	7,471	3,058	61	7,678	2,922	61	57.2%	-207.00 [-1268.41, 854.41]
Reynold 2014	7,413.02	2,281.76	8	6,246.17	1,583.66	6	15.7%	1166.85 [-859.42, 3193.12]
**Total (95% CI)**			**103**			**103**	**100.0%**	**-30.92 [-833.54, 771.70]**

Heterogeneity: Tau² = 0.00; Chi² = 1.99, df = 3 (P = 0.57); I² = 0%
Test for overall effect: Z = 0.08 (P = 0.94)

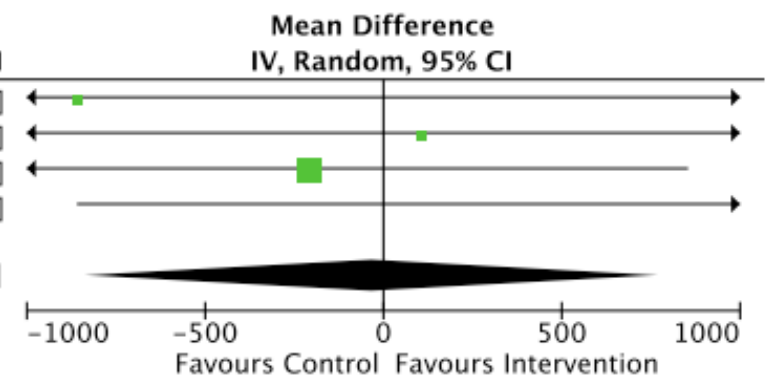

Key: CI= confidence interval, I^2 = heterogeneity test, IV = inverse variance, MVPA = moderate to vigorous physical activity, SD = standard deviation.

Appendix H. Sub-analysis according to baseline sleep-problem phenotype for the main meta-analysis outcomes for BMI and body weight.

A) Table summarising classification of studies within the systematic review according to the baseline poor sleep health problem (sleep phenotype).

Sleep problem phenotype	Criteria	Studies
**Insomnia**	Insomnia severity index score > 10 or fulfilling criteria for International Classification of Sleep Disorders.	Duncan (Refresh), Groeneveld, Ham, Irwin 2014, Irwin 2024, Johann
**Short sleep**	Objectively measured sleep duration < 6.5 hours (or time-in-bed/ total sleep time <7 hours) $	AL-Khatib, Chen, Chesla, Cizza *, Duncan (MES), Gonzalez, Haack, Hartescu, Ito-Masui, Killick, Leproult, Li, Mathew, Reutrakul, Reynold, So-ngern, Tasali
**Circadian misalignment**	Evening chronotype detected on a validated questionnaire	Erim
**Clinically significant sleep disorder**	A sleep problem detected (e.g. PSQI global score >5) without further qualification of the exact problem.	Logue *, Swinbourne, Zhang

Each study was allocated to one category only based on the available data in individual studies; however, in theory as sleep problems tend to overlap, it is possible some studies could have fitted into more than one category, if all sleep variables would have been measured. Sleep extension studies focused more on measuring sleep duration/ time-in-bed and less on measuring PSQI and insomnia scores and vice-versa for CBT-I and/ or sleep hygiene studies. *Cizza and Logue were not included in meta-analyses, $ definitions were made for the purposes of this study (other definitions exist, but those employed here were the most appropriate definitions given the data available). Key: PSQI = Pittsburgh Sleep Quality Index.

B) Forrest plots for the results of the meta-analysis examining the effect of sleep interventions according to baseline sleep-problem phenotype comparing short sleep and insomnia for outcomes of (i) body mass index and (ii)body weight

i) Body mass index (kg/m^2)

Study or Subgroup	Intervention Mean	Intervention SD	Intervention Total	Control Mean	Control SD	Control Total	Weight	Mean Difference IV, Random, 95% CI
**Short sleep**								
AlKhatib 2018	0.1	0.4	21	−0.004	0.47	21	33.0%	0.10 [−0.16, 0.37]
Chelsa 2020	29.7	3.9	12	30.3	3.7	14	3.5%	−0.60 [−3.54, 2.34]
Duncan (MES) 2020	30.81	3.83	14	29.29	3.9	23	4.4%	1.52 [−1.04, 4.08]
Haack 2013	26.8	1.2	13	26	1.3	9	15.9%	0.80 [−0.27, 1.87]
Hartescu 2021	29.2	2.22	10	29.19	3.93	8	3.2%	0.01 [−3.04, 3.06]
Li 2018	−0.8	0.5	16	−0.1	0.4	15	32.0%	−0.70 [−1.02, −0.38]
Reutrakul 2022	32.7	5.8	9	32	4.04	5	1.2%	0.70 [−4.49, 5.89]
So-ngern 2019	22.5	3.37	21	22.3	3.21	21	6.7%	0.20 [−1.79, 2.19]
**Subtotal (95% CI)**			**116**			**116**	**100.0%**	**0.01 [−0.57, 0.58]**
Heterogeneity: Tau² = 0.24; Chi² = 19.98, df = 7 (P = 0.006); I² = 65%								
Test for overall effect: Z = 0.02 (P = 0.98)								
**Insomnia**								
Duncan (Refresh) 2020	28.2	5.51	74	28.56	4.07	44	42.8%	−0.36 [−2.10, 1.38]
Groeneveld 2024	30.5	7.6	29	29.4	4.4	28	12.6%	1.10 [−2.11, 4.31]
Ham 2020	24.27	2.93	24	24.47	2.82	20	44.6%	−0.20 [−1.90, 1.50]
**Subtotal (95% CI)**			**127**			**92**	**100.0%**	**−0.11 [−1.24, 1.03]**
Heterogeneity: Tau² = 0.00; Chi² = 0.64, df = 2 (P = 0.73); I² = 0%								
Test for overall effect: Z = 0.18 (P = 0.86)								

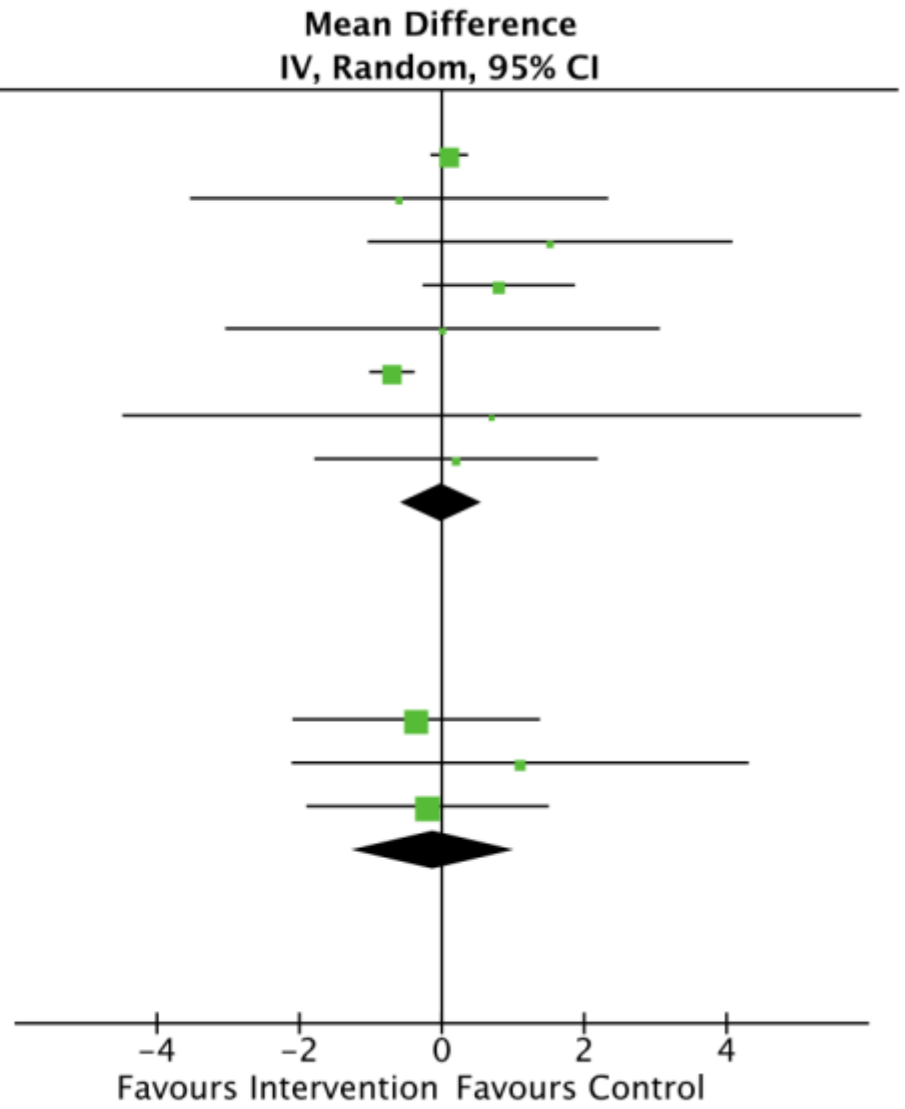

Test for subgroup differences: Chi² = 0.03, df = 1 (P = 0.86), I² = 0%

ii) Body weight (kg)

Study or Subgroup	Intervention Mean	Intervention SD	Intervention Total	Control Mean	Control SD	Control Total	Weight	Mean Difference IV, Random, 95% CI
**Short sleep**								
AlKhatib 2018	0.3	1.05	21	0.004	1.17	21	43.7%	0.30 [−0.38, 0.97]
Chelsa 2020	−4.4	13.83	11	−4.5	13.88	9	0.3%	0.10 [−12.11, 12.31]
Duncan (MES) 2020	87.92	14.95	14	82.63	13.86	23	0.5%	5.29 [−4.37, 14.95]
Hartescu 2021	94	6.7	10	92.5	10	8	0.7%	1.50 [−6.58, 9.58]
Leproult 2015	60	9.6	16	60	9.7	16	1.1%	0.00 [−6.69, 6.69]
Reutrakul 2022	85.44	15.49	9	84.02	12.4	5	0.2%	1.42 [−13.43, 16.27]
So-ngern 2019	56.6	10.2	21	56.2	9.63	21	1.3%	0.40 [−5.60, 6.40]
Tasali 2022	−0.48	1.19	40	0.39	1.19	40	52.1%	−0.87 [−1.39, −0.35]
**Subtotal (95% CI)**			**142**			**143**	**100.0%**	**−0.28 [−0.98, 0.42]**
Heterogeneity: Tau² = 0.17; Chi² = 8.92, df = 7 (P = 0.26); I² = 22%								
Test for overall effect: Z = 0.78 (P = 0.44)								
**Insomnia**								
Duncan (Refresh) 2020	79.92	19.54	74	81.01	14.11	44	79.2%	−1.09 [−7.19, 5.01]
Groeneveld 2024	1.24	26.75	25	−1.49	14.89	27	20.8%	2.73 [−9.17, 14.63]
**Subtotal (95% CI)**			**99**			**71**	**100.0%**	**−0.29 [−5.72, 5.13]**
Heterogeneity: Tau² = 0.00; Chi² = 0.31, df = 1 (P = 0.58); I² = 0%								
Test for overall effect: Z = 0.11 (P = 0.92)								

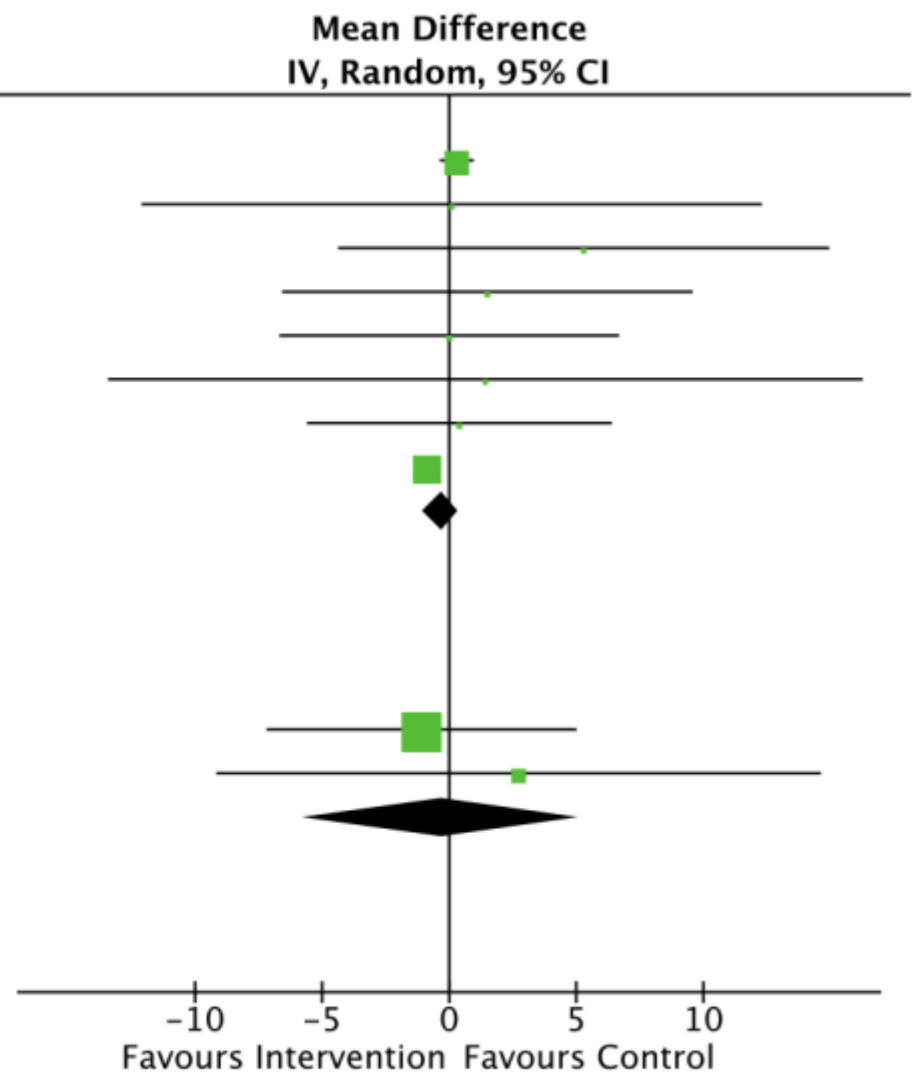

Test for subgroup differences: Chi² = 0.00, df = 1 (P = 0.99), I² = 0%

Key: CI= confidence interval, I^2= heterogeneity test, IV = inverse variance, SD = standard deviation. The test for sub-group differences was not significant for either outcome of BMI or body weight.

Appendix I. Risk of publication bias analysis using funnel plots for outcomes of (a) BMI, (b) body weight and (c) daily calorie intake before and after removal of smaller studies and (d) results of Egger's regression-based tests.

(A) (i) Funnel plot for BMI outcome for all studies (n=13)

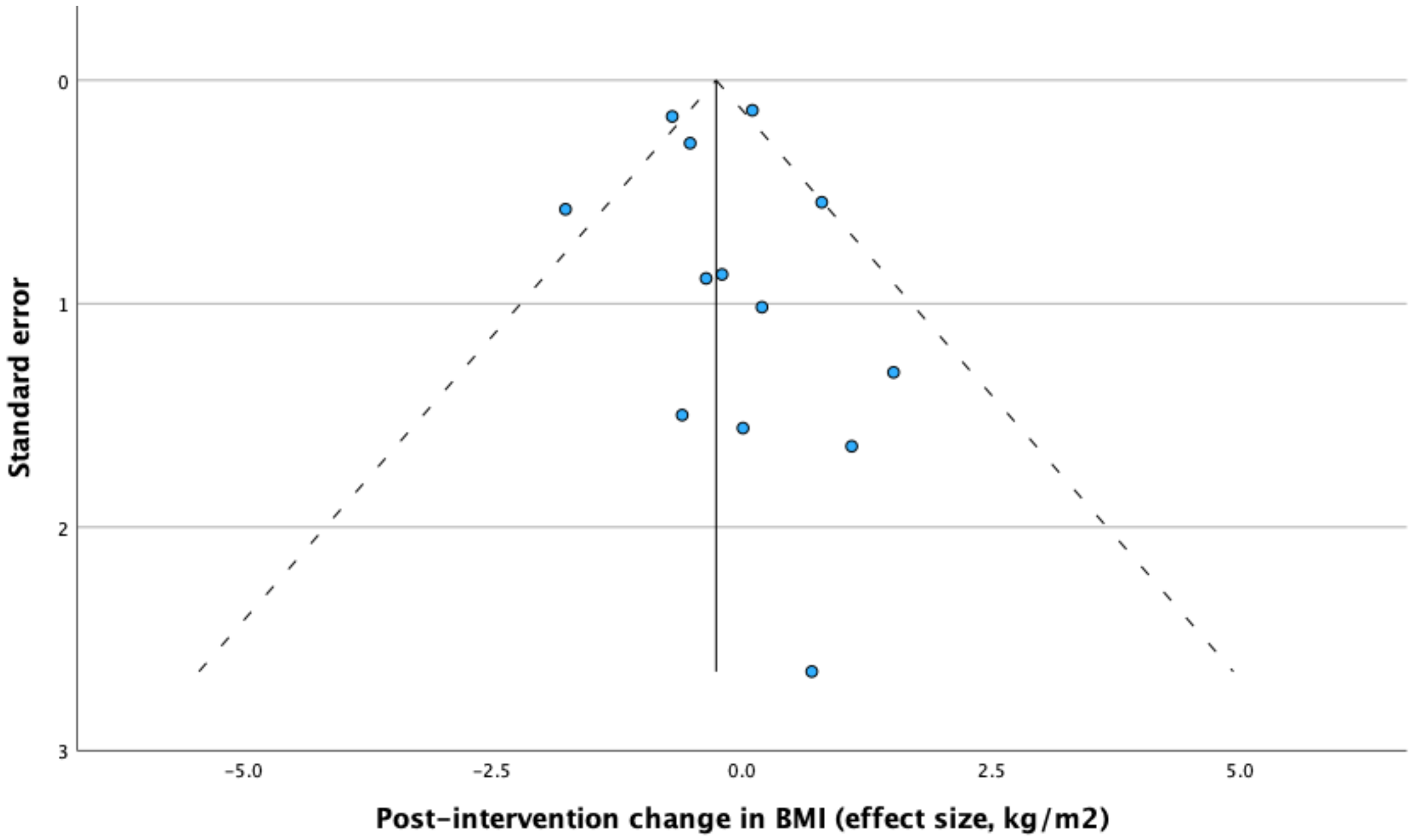

(A) (ii) Funnel plot for BMI after removal of smaller studies (remaining n=2)

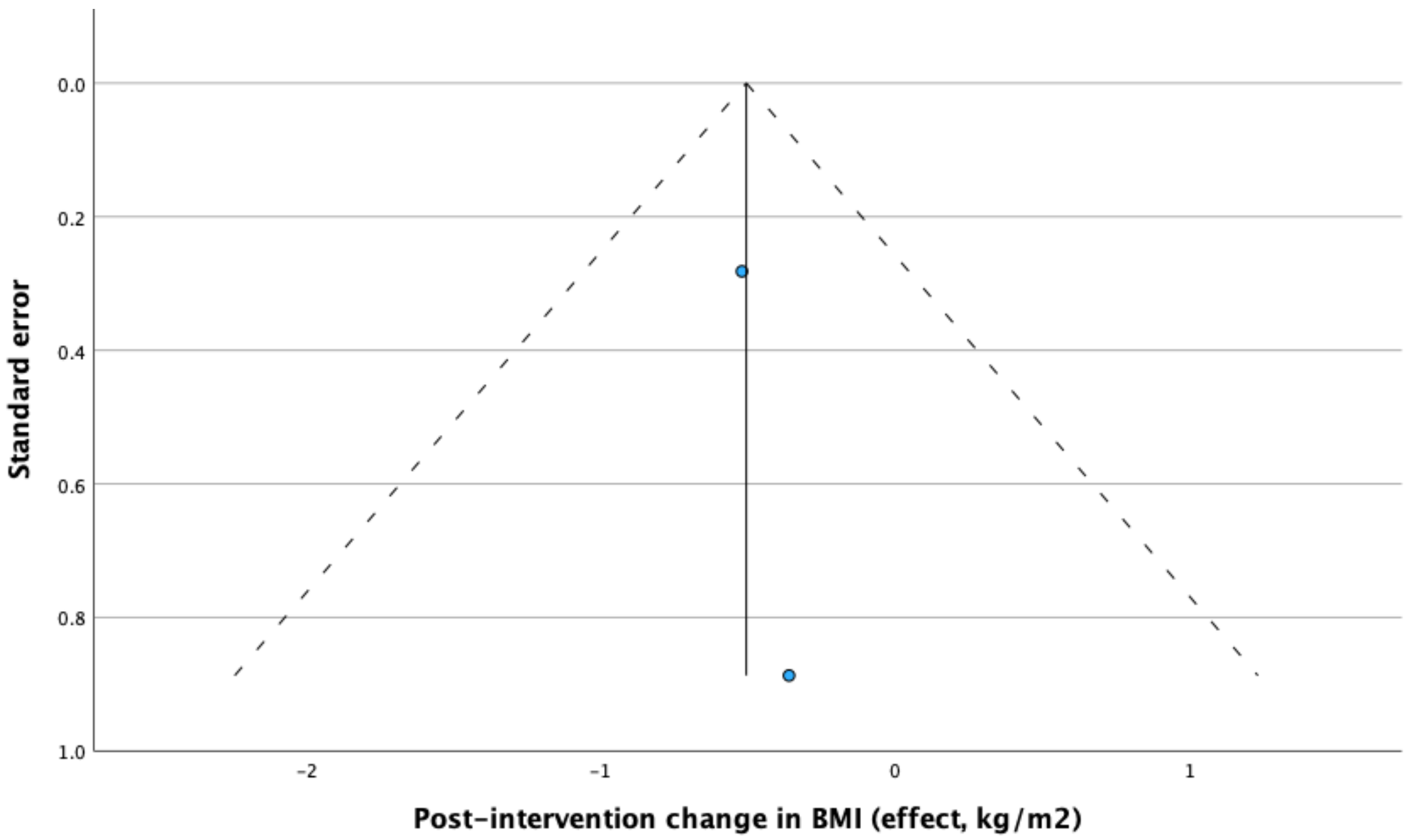

(B) (i) Funnel plot for body weight for all studies (n=13)

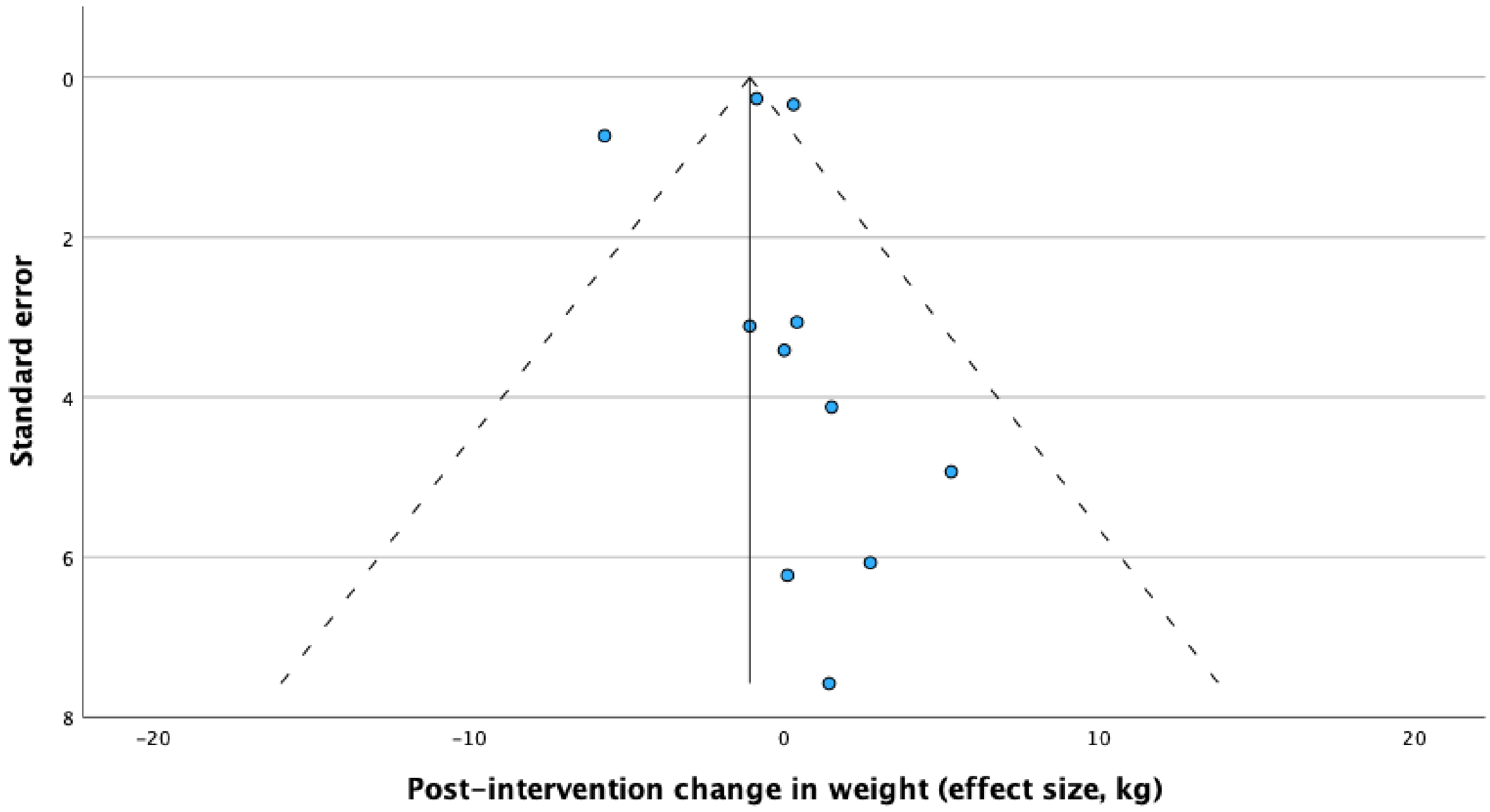

(B) (ii) Funnel plot for body weight after removal of smaller studies (remaining n=2)

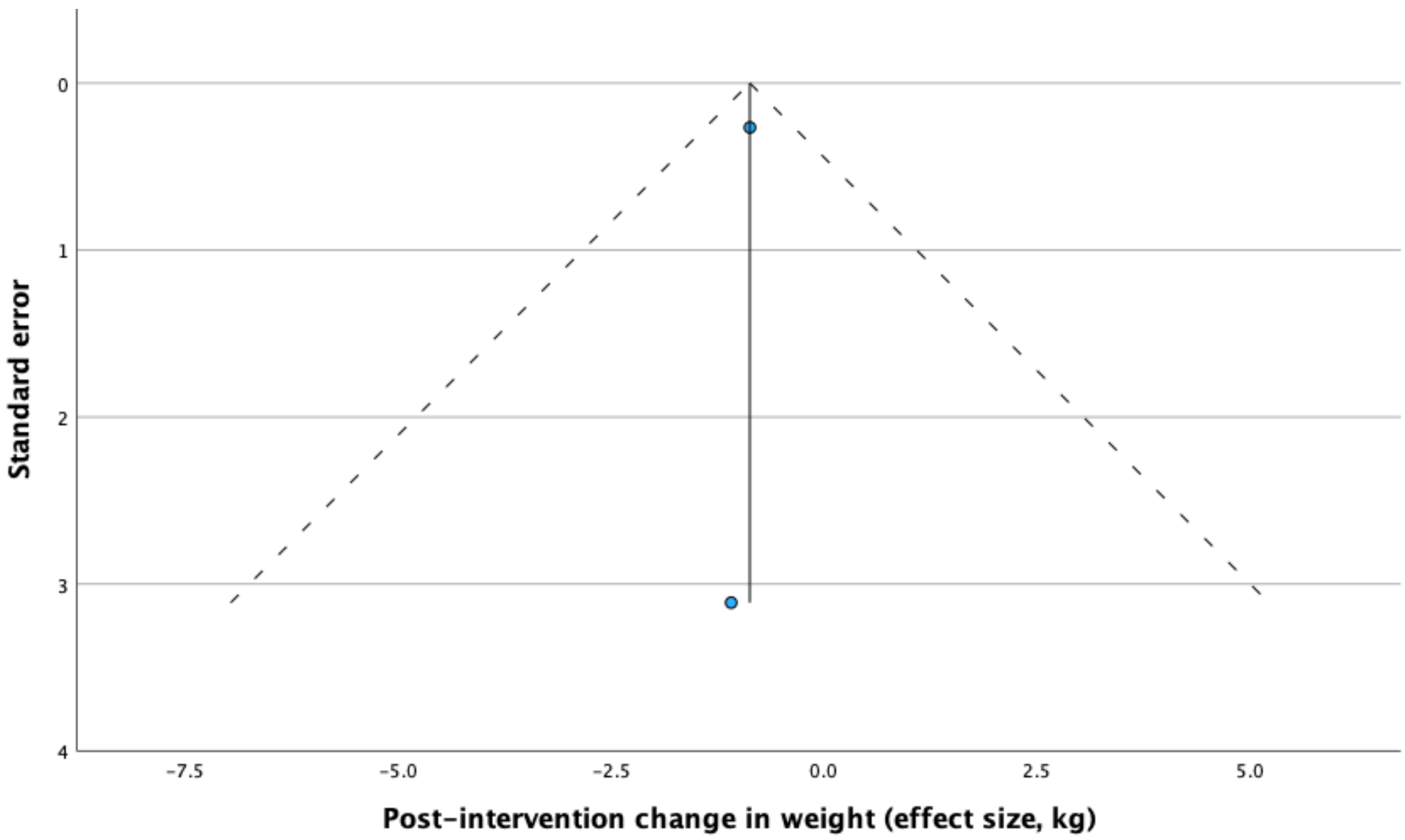

(C) (i) Funnel plot for daily calorie intake for all studies (5 studies, 223 people) *

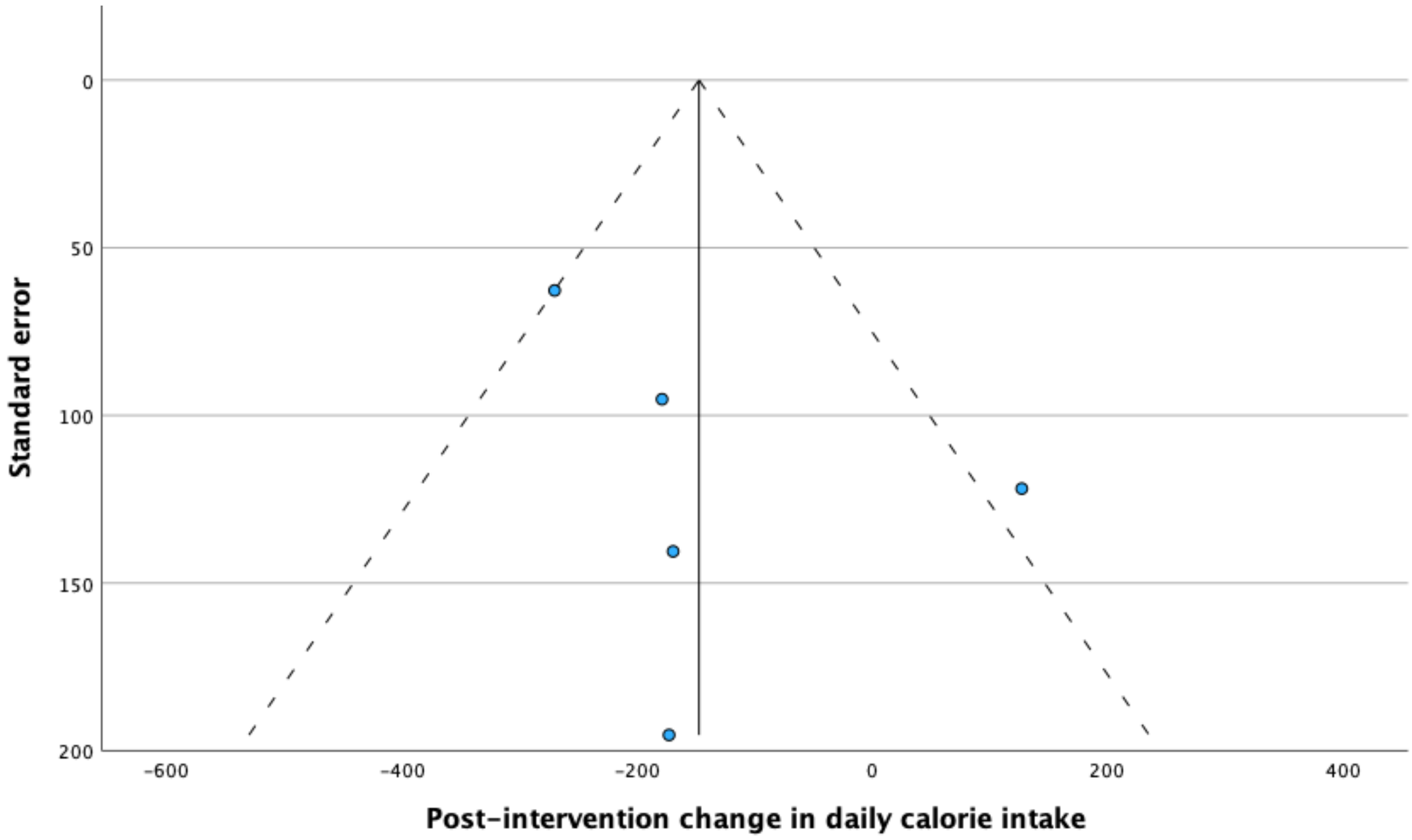

* As 4 of the 5 studies assessing daily calorie intake were deemed smaller studies, a separate analysis after removal of smaller studies was not performed, as only one study was left.

Within each graph, a single dot represents a primary study, the horizontal continuous line represents the overall effect size for the outcome between all studies and the diagonal sloping dashed lines represent the 95% confidence intervals for the effect size.

D) Table for the results of Egger's regression-based tests to assess for the risk of publication bias before and after removal of smaller studies for outcomes of BMI, bodyweight and daily calorie intake.

Outcome	Smaller studies	Studies analysed	Intercept Co-efficient (95% CI)	P-value
BMI	included	13	-0.49 (-1.17, 0.19)	0.157
BMI	removed	2	n/a *	-
Body weight	included	11	-2.119 (-4.54, 0.30)	0.086
Body weight	removed	2	n/a *	-
Daily calorie intake	included	5	-292.8 (-684.3, 98.6)	0.143

Daily calorie intake	removed	1	n/a *	-

Smaller studies were defined as having less than 60 participants (Reference: Teare et al.,. 2014: doi: 10.1186/1745-6215-15-264). * Egger's regression test could not be calculated when there were two studies or less. Overall, the results demonstrate a low risk of publication bias and also, that throughout the three outcomes the majority of studies were small-to-medium sized. **Key:** BMI = body mass index, CI = confidence interval, kg = kilogram, m = metre, N = number.

Appendix J. Results of the meta-analysis employing a 'leave one-out-analysis' for main outcomes of BMI and body weight

a) BMI (kg/m^2)

Study omitted	N	Post-intervention change (95% CI), kg/m^2	P-value	I^2 (%)
Total cohort (no omissions)	1575	-0.30 (-0.75, 0.16)	0.20	60
AlKhatib 2018	1480	-0.40 (-0.84, 0.04)	0.08	31
Chesla 2020	1496	-0.25 (-0.70, 0.20)	0.28	62
Duncan (MES) 2020	1485	-0.31 (-0.75, 0.13)	0.17	60
Duncan (Refresh) 2020	1404	-0.25 (-0.71, 0.21)	0.29	62
Erim 2024	1484	-0.14 (-0.55, 0.27)	0.50	50
Groeneveld 2024	1465	-0.28 (-0.73, 0.16)	0.21	61
Haack 2013	1500	-0.38 (-0.82, 0.07)	0.10	56
Ham 2020	1478	-0.26 (-0.72, 0.20)	0.27	62
Hartescu 2021	1504	-0.26 (-0.71, 0.19)	0.25	62
Li 2018	1491	-0.15 (-0.61, 0.31)	0.53	38
Reutrakul 2022	1508	-0.26 (-0.71, 0.18)	0.25	62
So-ngern 2019	1480	-0.28 (-0.73, 0.18)	0.23	62
Zhang 2021	489	-0.20 (-0.71, 0.32)	0.45	61

b) Body weight (kg)

Study omitted	N	Post-intervention change (95% CI), kg	P-value	I^2 (%)
Total cohort (no omissions)	546	-1.32 (-3.12, 0.49)	0.15	82
AlKhatib 2018	415	-0.94 (-3.62, 1.73)	0.49	78
Chesla 2020	473	-1.11 (-2.93, 0.72)	0.24	84
Duncan (MES) 2020	456	-1.29 (-3.10, 0.52)	0.16	84
Duncan (Refresh) 2020	375	-1.08 (-2.95, 0.80)	0.26	84
Erim 2024	455	-0.38 (-0.83, 0.08)	0.10	3
Groeneveld 2024	441	-1.17 (-2.99, 0.66)	0.21	84
Hartescu 2021	475	-1.19 (-3.04, 0.65)	0.20	84
Leproult 2015	461	-1.14 (-3.01, 0.72)	0.23	84
Reutrakul 2022	479	-1.12 (-2.93, 0.70)	0.23	84
So-ngern 2019	451	-1.18 (-3.06, 0.69)	0.22	84
Tasali 2022	413	-0.42 (-3.51, 2.67)	0.79	84

Key: BMI = body mass index, CI = confidence intervals, I^2 = I^2 for heterogeneity, kg = kilogram, N = number of participants. Removal of single studies one at a time did not change the significance of results for either BMI or weight outcomes (all p-values >0.05) and thus, demonstrates that there was no individual 'outlier' study influencing the overall results.

Appendix K: Forrest plots for the results of the meta-analysis examining the effect of sleep interventions according to the risk of bias category on (a) BMI and (b) body weight.

(a) BMI (kg/m²)

Study or Subgroup	Intervention Mean	Intervention SD	Intervention Total	Control Mean	Control SD	Control Total	Weight	Mean Difference IV, Random, 95% CI
**Low risk**								
Duncan (MES) 2020	30.81	3.83	14	29.29	3.9	23	17.5%	1.52 [−1.04, 4.08]
Duncan (Refresh) 2020	28.2	5.51	74	28.56	4.07	44	38.0%	−0.36 [−2.10, 1.38]
Groeneveld 2024	30.5	7.6	29	29.4	4.4	28	11.2%	1.10 [−2.11, 4.31]
Reutrakul 2022	32.7	5.8	9	32	4.04	5	4.3%	0.70 [−4.49, 5.89]
So−ngern 2019	22.5	3.37	21	22.3	3.21	21	29.0%	0.20 [−1.79, 2.19]
**Subtotal (95% CI)**			**147**			**121**	**100.0%**	**0.34 [−0.73, 1.41]**
Heterogeneity: Tau² = 0.00; Chi² = 1.69, df = 4 (P = 0.79); I² = 0%								
Test for overall effect: Z = 0.62 (P = 0.53)								
**Some concerns**								
AlKhatib 2018	0.1	0.4	21	−0.004	0.47	21	23.9%	0.10 [−0.16, 0.37]
Erim 2024	29.03	1.91	19	30.8	1.64	19	11.5%	−1.77 [−2.90, −0.64]
Haack 2013	26.8	1.2	13	26	1.3	9	12.2%	0.80 [−0.27, 1.87]
Ham 2020	24.27	2.93	24	24.47	2.82	20	6.8%	−0.20 [−1.90, 1.50]
Hartescu 2021	29.2	2.22	10	29.19	3.93	8	2.6%	0.01 [−3.04, 3.06]
Li 2018	−0.8	0.5	16	−0.1	0.4	15	23.2%	−0.70 [−1.02, −0.38]
Zhang 2021	24.76	4	506	25.28	5.02	527	19.8%	−0.52 [−1.07, 0.03]
**Subtotal (95% CI)**			**609**			**619**	**100.0%**	**−0.36 [−0.88, 0.16]**
Heterogeneity: Tau² = 0.28; Chi² = 26.15, df = 6 (P = 0.0002); I² = 77%								
Test for overall effect: Z = 1.36 (P = 0.17)								

Test for subgroup differences: Chi² = 1.33, df = 1 (P = 0.25), I² = 24.5%

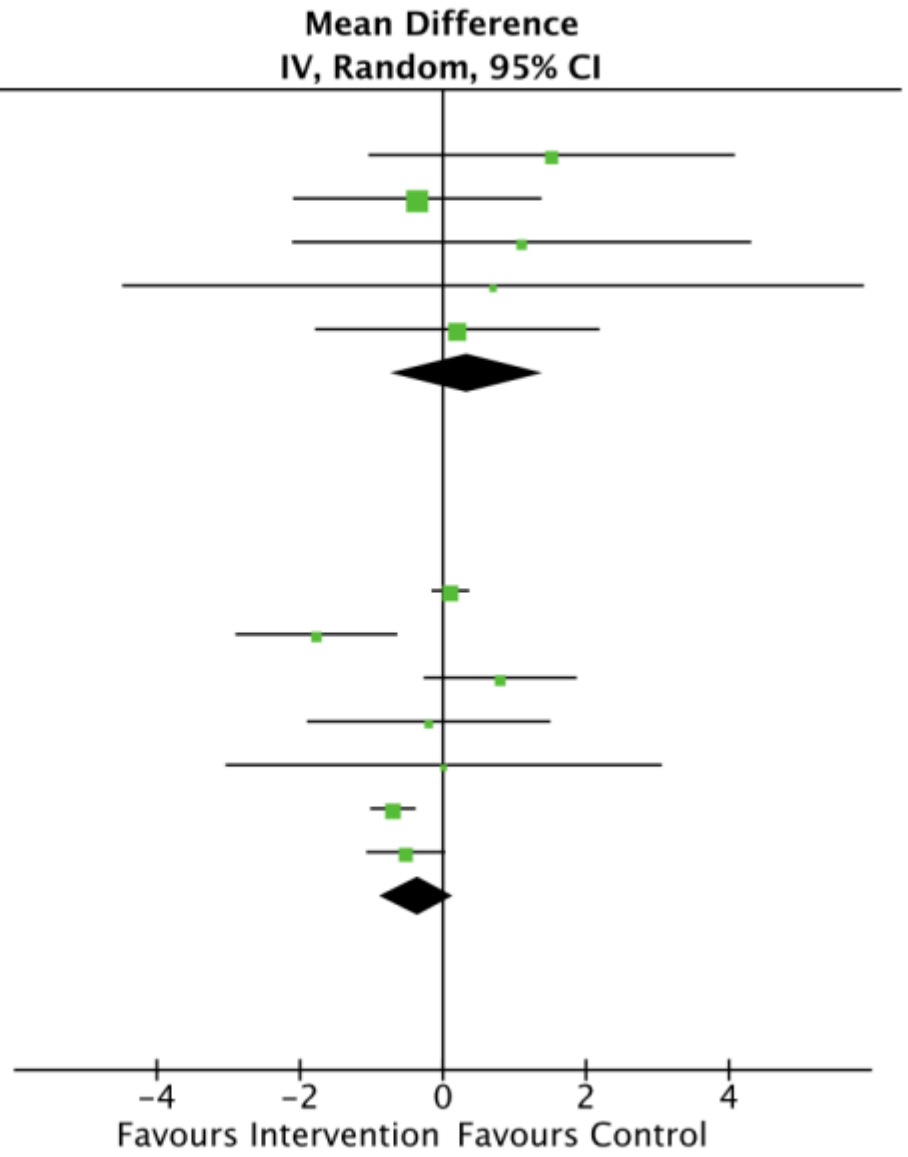

(b) Body weight (kg)

Study or Subgroup	Intervention Mean	Intervention SD	Intervention Total	Control Mean	Control SD	Control Total	Weight	Mean Difference IV, Random, 95% CI
**Low Risk**								
Duncan (MES) 2020	87.92	14.95	14	82.63	13.86	23	0.3%	5.29 [−4.37, 14.95]
Duncan (Refresh) 2020	79.92	19.54	74	81.01	14.11	44	0.7%	−1.09 [−7.19, 5.01]
Groeneveld 2024	1.24	26.75	25	−1.49	14.89	27	0.2%	2.73 [−9.17, 14.63]
Reutrakul 2022	85.44	15.49	9	84.02	12.4	5	0.1%	1.42 [−13.43, 16.27]
So−ngern 2019	56.6	10.2	21	56.2	9.63	21	0.7%	0.40 [−5.60, 6.40]
Tasali 2022	−0.48	1.19	40	0.39	1.19	40	97.9%	−0.87 [−1.39, −0.35]
**Subtotal (95% CI)**			**183**			**160**	**100.0%**	**−0.84 [−1.35, −0.32]**
Heterogeneity: Tau² = 0.00; Chi² = 2.16, df = 5 (P = 0.83); I² = 0%								
Test for overall effect: Z = 3.17 (P = 0.002)								
**Some concerns**								
AlKhatib 2018	0.3	1.05	21	0.004	1.17	21	32.9%	0.30 [−0.38, 0.97]
Erim 2024	−9.38	2.8	19	−3.69	1.53	19	32.1%	−5.69 [−7.12, −4.26]
Hartescu 2021	94	6.7	10	92.5	10	8	16.0%	1.50 [−6.58, 9.58]
Leproult 2015	60	9.6	16	60	9.7	16	19.1%	0.00 [−6.69, 6.69]
**Subtotal (95% CI)**			**66**			**64**	**100.0%**	**−1.49 [−5.97, 3.00]**
Heterogeneity: Tau² = 15.79; Chi² = 55.18, df = 3 (P < 0.00001); I² = 95%								
Test for overall effect: Z = 0.65 (P = 0.52)								

Test for subgroup differences: Chi² = 0.08, df = 1 (P = 0.78), I² = 0%

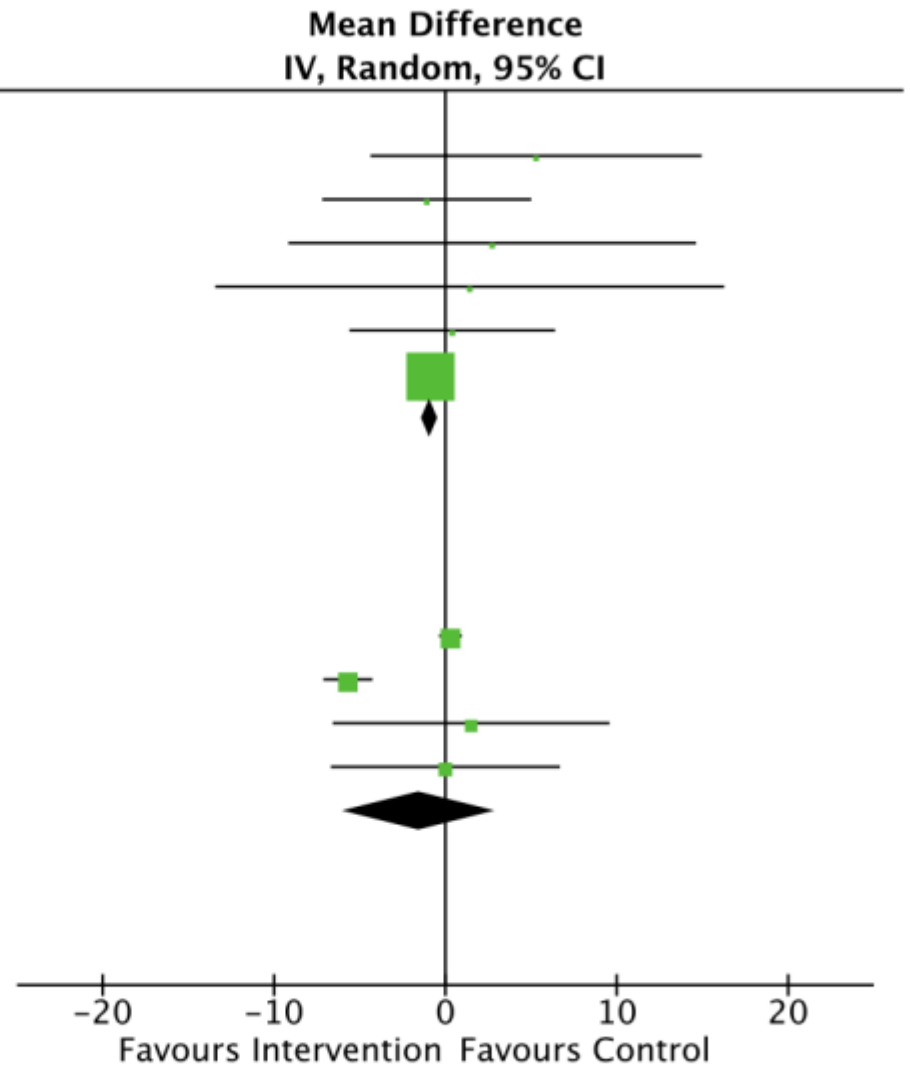

Risk of bias categories were taken from Traffic-light plots in Appendix B. The categories of 'moderate' and 'serious' concerns from ROBINS-I assessments were converted to 'some concerns' and 'high risk' on ROB-2 assessments for overall comparison. Only risk of bias

categories that contained three or more studies were used in the above analysis, hence the category 'high risk' did not feature. Overall, as the test for sub-group differences was not significant for either BMI (p=0.25) or body weight (p=0.78), this suggests the risk of bias category was not an influence on the overall meta-analysis results. Key: BMI = body mass index, CI= confidence interval, I^2= heterogeneity test, IV = inverse variance, kg = kilogram, m = metre, SD = standard deviation.

Appendix L. Forrest plots for the results of the meta-analysis assessing the effect of sleep interventions on anthropometric measures after removal of studies featuring self-measured height and weight.

(a) **Removal of studies featuring self-measured height and weight.**

(i) BMI (kg/m^2)

	Intervention			Control				Mean Difference
Study or Subgroup	Mean	SD	Total	Mean	SD	Total	Weight	IV, Random, 95% CI
AlKhatib 2018	0.1	0.4	21	−0.004	0.47	21	21.7%	0.10 [−0.16, 0.37]
Chelsa 2020	29.7	3.9	12	30.3	3.7	14	2.2%	−0.60 [−3.54, 2.34]
Duncan (MES) 2020	30.81	3.83	14	29.29	3.9	23	2.8%	1.52 [−1.04, 4.08]
Erim 2024	29.03	1.91	19	30.8	1.64	19	9.7%	−1.77 [−2.90, −0.64]
Groeneveld 2024	30.5	7.6	29	29.4	4.4	28	1.9%	1.10 [−2.11, 4.31]
Haack 2013	26.8	1.2	13	26	1.3	9	10.3%	0.80 [−0.27, 1.87]
Ham 2020	24.27	2.93	24	24.47	2.82	20	5.6%	−0.20 [−1.90, 1.50]
Hartescu 2021	29.2	2.22	10	29.19	3.93	8	2.1%	0.01 [−3.04, 3.06]
Li 2018	−0.8	0.5	16	−0.1	0.4	15	21.0%	−0.70 [−1.02, −0.38]
Reutrakul 2022	32.7	5.8	9	32	4.04	5	0.8%	0.70 [−4.49, 5.89]
So-ngern 2019	22.5	3.37	21	22.3	3.21	21	4.4%	0.20 [−1.79, 2.19]
Zhang 2021	24.76	4	506	25.28	5.02	527	17.5%	−0.52 [−1.07, 0.03]
**Total (95% CI)**			**694**			**710**	**100.0%**	**−0.25 [−0.71, 0.21]**

Heterogeneity: Tau^2 = 0.24; Chi^2 = 29.07, df = 11 (P = 0.002); I^2 = 62%
Test for overall effect: Z = 1.07 (P = 0.29)

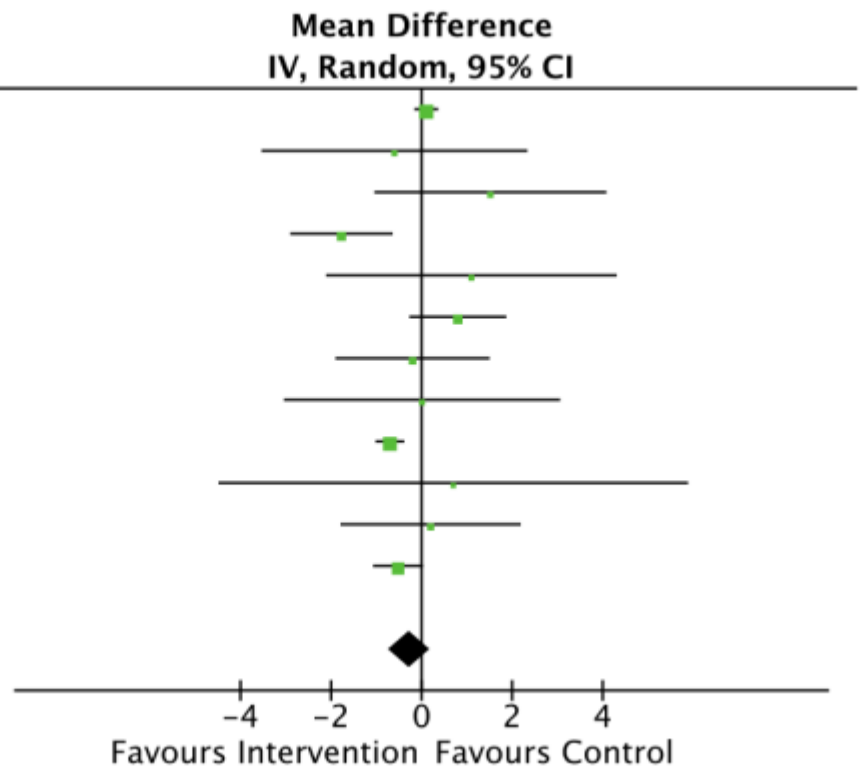

(ii) Body weight (kg)

	Intervention			Control				Mean Difference
Study or Subgroup	Mean	SD	Total	Mean	SD	Total	Weight	IV, Random, 95% CI
AlKhatib 2018	0.3	1.05	21	0.004	1.17	21	25.2%	0.30 [−0.38, 0.97]
Chelsa 2020	−4.4	13.83	11	−4.5	13.88	9	2.2%	0.10 [−12.11, 12.31]
Duncan (MES) 2020	87.92	14.95	14	82.63	13.86	23	3.3%	5.29 [−4.37, 14.95]
Erim 2024	−9.38	2.8	19	−3.69	1.53	19	22.6%	−5.69 [−7.12, −4.26]
Groeneveld 2024	1.24	26.75	25	−1.49	14.89	27	2.3%	2.73 [−9.17, 14.63]
Hartescu 2021	94	6.7	10	92.5	10	8	4.5%	1.50 [−6.58, 9.58]
Leproult 2015	60	9.6	16	60	9.7	16	6.0%	0.00 [−6.69, 6.69]
Reutrakul 2022	85.44	15.49	9	84.02	12.4	5	1.5%	1.42 [−13.43, 16.27]
So-ngern 2019	56.6	10.2	21	56.2	9.63	21	7.1%	0.40 [−5.60, 6.40]
Tasali 2022	−0.48	1.19	40	0.39	1.19	40	25.5%	−0.87 [−1.39, −0.35]
**Total (95% CI)**			**186**			**189**	**100.0%**	**−1.08 [−2.95, 0.80]**

Heterogeneity: Tau^2 = 3.51; Chi^2 = 57.39, df = 9 (P < 0.00001); I^2 = 84%
Test for overall effect: Z = 1.13 (P = 0.26)

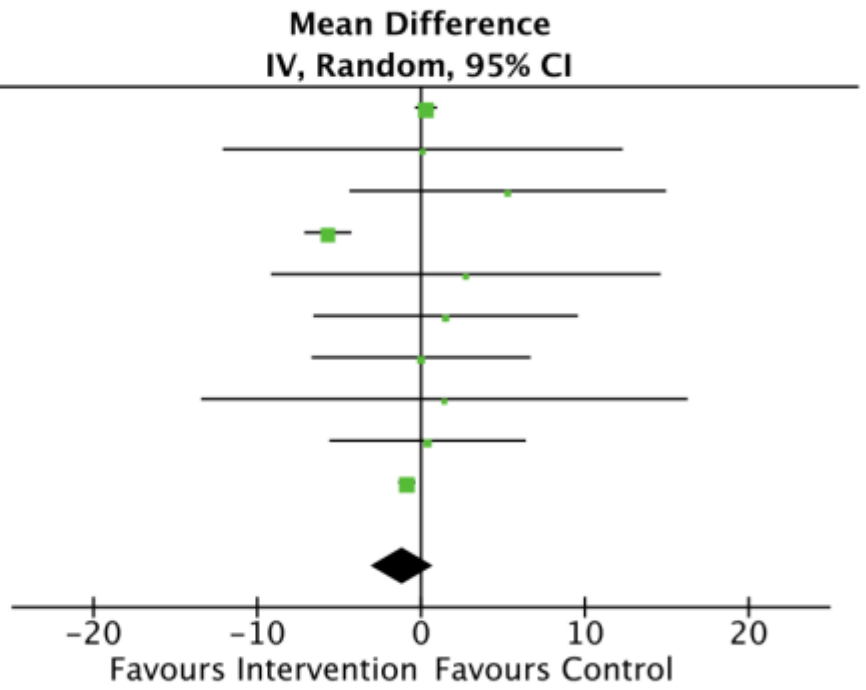

Body weight is measured in kilograms, BMI in kg/m^2. Key: BMI = body mass index, CI= confidence interval, CBT-I = Cognitive Behavioural Therapy for Insomnia, I^2= heterogeneity test, IV = inverse variance, kg = kilogram, SD = standard deviation. Only one study was removed (Duncan et al 2020 REFRESH)

Appendix M. Bubble plots for the results of meta-regression models for the association of baseline BMI level in each study and outcomes of post-intervention change in (a) BMI level and (b) body weight

(a) BMI as the outcome measure

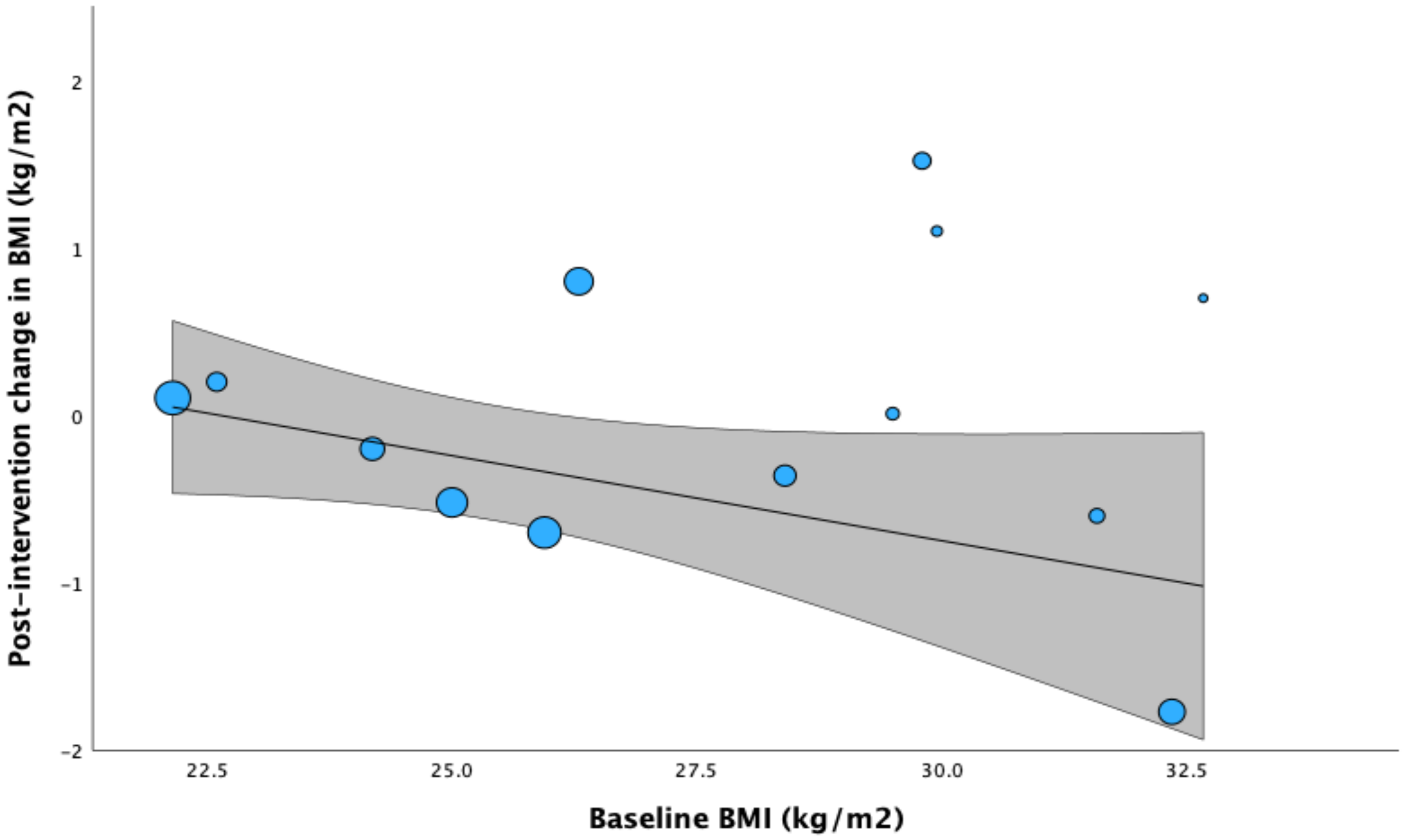

(b) Body weight as the outcome measure

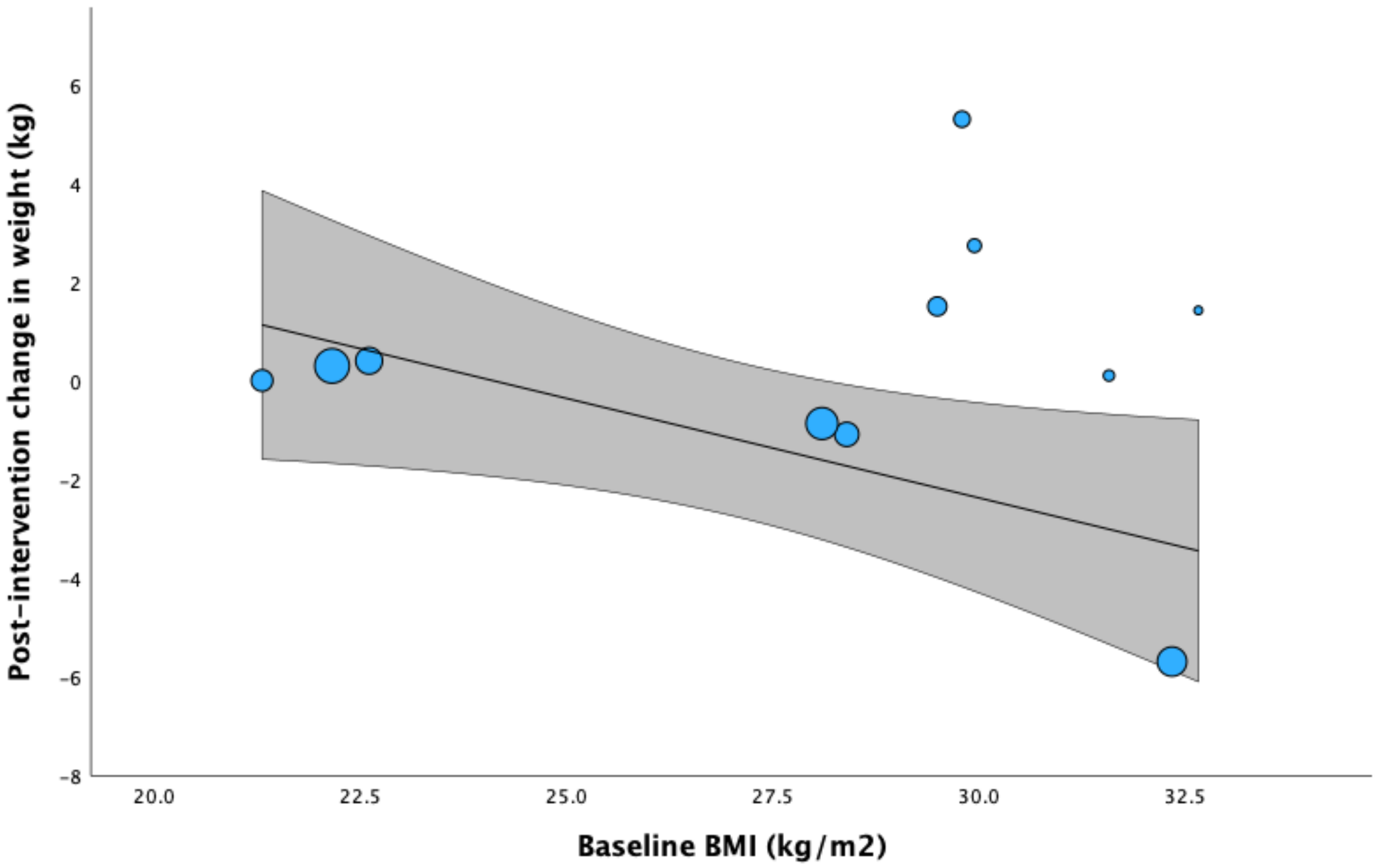

The univariate meta-regression models were formed using random effects function for each model. Each study, represented by a dot, was assessed for the change in weight or BMI (effect size), where the larger sized dot represents a stronger weighted study, which in turn

have a stronger influence on the meta-regression prediction line. The grey zone represents the 95% confidence intervals.

Appendix N. GRADE assessment summary of findings

Assessment	Risk of Bias	Inconsistency of results	Indirectness	Imprecision	Publication bias	Final certainty
Body mass index	Some concerns	Consistent	Low concern	Some	Low	Moderate
Body weight	Some concerns	Some inconsistency	Low concern	Some	Low	Low
Daily calorie intake	Some concerns	Consistent	Low concern	Some	Low	Moderate

Key: GRADE = Grading of recommendations, assessments, development and evaluation.
